# Gene set enrichment analysis of curated monogenic loci highlights key pathways and multisystem involvement in male infertility

**DOI:** 10.1186/s12610-026-00316-2

**Published:** 2026-06-10

**Authors:** Matthew Yang, Katreya Lovrenert, Nannan Thirumavalavan, Michael L. Eisenberg, Fredrick R. Schumacher, Chen-Han Wilfred Wu

**Affiliations:** 1 Department of Genetics, Heersink School of Medicine, Hugh Kaul Precision Medicine Institute, University of Alabama, Birmingham, AL USA; 2 Department of Urology, Heersink School of Medicine, Hugh Kaul Precision Medicine Institute, University of Alabama, Birmingham, AL USA; 3 Department of Genetics and Genome Sciences, Case Western Reserve University School of Medicine and University Hospitals, Cleveland, OH USA; 4 Department of Urology, Case Western Reserve University School of Medicine and University Hospitals, Cleveland, OH USA; 5 Department of Urology, School of Medicine, Stanford University, Stanford, CA USA; 6 Department of Population and Quantitative Health Sciences, Case Western Reserve University School of Medicine, Cleveland, OH USA

**Keywords:** Male infertility, Genetics, Gene set enrichment analysis, Gene ontology, Overall health

## Abstract

**Background:**

Male infertility affects approximately 10% of men globally, yet approximately 70% of cases lack a definitive genetic diagnosis. Next-generation sequencing has identified numerous monogenic causes, many of which are linked to broader systemic conditions. We curated a list of 596 candidate genes associated with male infertility from the literature and clinical genetics panels. Gene set enrichment analyses were performed on the strong-evidence genes using g: Profiler, REVIGO, and PANTHER to identify overrepresented Gene Ontology (GO) terms, and Reactome pathways. Gene–tissue expression profiles were assessed using GTExv8.

Among the 178 strong-evidence genes, enrichment analyses revealed significant overrepresentation of terms related to ciliary and flagellar function (e.g., cilium movement, dynein complex), endocrine signaling (e.g., hormone receptor binding, steroid biosynthesis), and DNA repair mechanisms, particularly the Fanconi anemia nuclear complex. Reactome pathway analysis identified overrepresentation of androgen, glucocorticoid, and mineralocorticoid biosynthesis, BBSome-mediated ciliary transport, and meiosis. Tissue expression clustering identified extra-gonadal expression patterns, implicating adrenal, hypothalamic, and systemic involvement in genetic forms of male infertility.

**Conclusions:**

Our gene set enrichment and tissue expression analyses suggest that monogenic causes of male infertility are enriched in pathways in ciliary motility, endocrine regulation, and DNA repair which also have roles in extra-gonadal organ systems. These findings are consistent with epidemiological observations linking male infertility to broader health conditions and support multidisciplinary evaluation and care for affected individuals.

**Supplementary Information:**

The online version contains supplementary material available at 10.1186/s12610-026-00316-2.

## Résumé

**Contexte:**

L’infertilité masculine touche environ 10 % des hommes dans le Monde, pourtant environ 70 % des cas ne présentent pas de diagnostic génétique définitif. Le séquençage de nouvelle génération a identifié de nombreuses causes monogéniques, dont beaucoup sont liées à des conditions systémiques plus larges. Nous avons sélectionné une liste de 596 gènes candidats associés à l’infertilité masculine à partir de la littérature et des panels de génétique clinique. Des analyses d’enrichissement de l’ensemble génétique ont été réalisées sur les gènes à forte preuve en utilisant Profiler, REVIGO et PANTHER pour identifier les termes surreprésentés de l’ontologie génique et les voies réactomes. Les profils d’expression gène-tissu ont été évalués à l’aide de GTExv8.

**Résultats:**

Parmi les 178 gènes à preuve solide, les analyses d’enrichissement ont révélé une surreprésentation significative de termes liés à la fonction ciliaire et flagellaire (par exemple, mouvement cilié, complexe dynéinique), à la signalisation endocrinienne (par exemple, liaison des récepteurs hormonaux, biosynthèse des stéroïdes) et aux mécanismes de réparation de l’ADN, en particulier le complexe nucléaire de l’anémie de Fanconi. L’analyse des voies réactomes a identifié une surreprésentation de la biosynthèse androgène, glucocorticoïde et minéralocorticoïde, du transport ciliaire médié par le syndrome de Bardet-Biedl, et de la méiose. Le regroupement d’expression tissulaire a identifié des schémas d’expression extra-gonadiques, comportant une implication surrénalienne, hypothalamique et systémique dans les formes génétiques d’infertilité masculine.

**Conclusions:**

Nos analyses d’enrichissement des ensembles génétiques et d’expression tissulaire suggèrent que les causes monogéniques de l’infertilité masculine sont enrichies dans les voies de la motilité ciliaire, de la régulation endocrinienne et de la réparation de l’ADN, qui jouent également un rôle dans les systèmes d’organes extra-gonadiques. Ces résultats sont cohérents avec des observations épidémiologiques liant l’infertilité masculine à des conditions de santé plus larges et soutiennent une évaluation et des soins multidisciplinaires pour les personnes concernées.

**Mots-clés:**

Infertilité masculine, Génétique, Analyse d’Enrichissement des Ensembles géniques, Ontologie génique, Santé générale

## Introduction

Male infertility is a heterogeneous condition that affects approximately 10% [1] of men worldwide. Despite its prevalence, the underlying causes often remain elusive, with most infertile men (about 70%) lacking a clear genetic diagnosis [2]. The genetic contributions to male infertility are complex and multifaceted, involving various genes and chromosomal regions crucial for reproductive function [3]. Male infertility has also been increasingly linked to broader systemic health issues. Infertile men often present with comorbid conditions, indicating that infertility may be a marker of general health status [4].

The genetic component of male infertility has long been recognized and researched. The earliest discoveries involved chromosomal abnormalities and chromosomal microdeletions [5]. One well-studied genetic factor is the Azoospermia Factor (AZF) region on the long arm of the Y chromosome (Yq) [1]. This region contains genes essential for spermatogenesis. Yq microdeletions within the AZF region have been traditionally associated with male infertility, particularly in cases of non-obstructive azoospermia and severe oligospermia. Earlier estimates suggested that Yq microdeletions are present in 8–12% of males with non-obstructive azoospermia and 3–7% with severe oligospermia [6].

However, recent studies have challenged the previously held significance of Yq microdeletions. A meta-analysis assessing the frequency of Yq microdeletions in severely oligospermic males in North America and Europe found that these deletions were present in only 0.8% of males with sperm concentrations between 1 and 5 million sperm/mL [7]. This significant lower prevalence suggests that, although Y chromosome microdeletions remain a recognized factor in male infertility, their relative contribution has diminished as understanding of other genetic causes has expanded. The increasing application of next-generation sequencing (NGS) studies to male infertility has identified numerous monogenic causes of male infertility (2), which form the basis of the present study.

Here, we aim to leverage the growing number of monogenic loci associated with male infertility to identify biological pathways that may warrant clinical evaluation or monitoring for associated systemic conditions. To achieve this, we will systematically review and curate a set of monogenic loci linked to male infertility (Supplementary Table 1). Utilizing gene ontology [8], Reactome pathways [9], and genotype-tissue expression analysis [10], we will identify overrepresented biological processes, cellular components, molecular functions, and enzymatic pathways among the genes. This approach aims to elucidate broader pathophysiological mechanisms implicated in genetic male infertility and to inform clinical care regarding overall health and potential comorbidities in affected individuals.

## Methods

### Identification of monogenic causes of male infertility

**Fig. 1:**
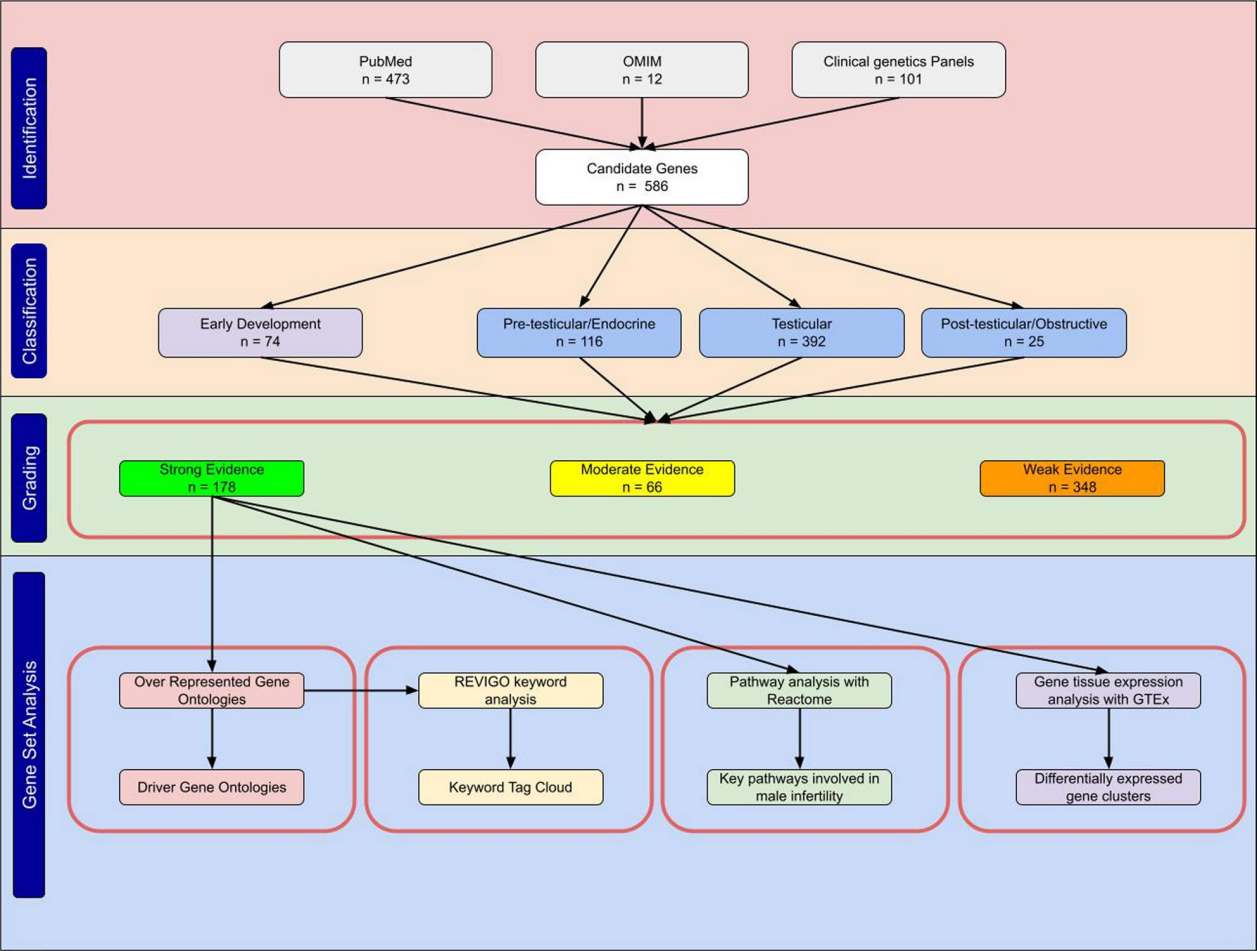
Workflow for gene identification, classification, and analysis in male infertility

We performed a literature search with the PubMed search engine (https://www.ncbi.nlm.nih.gov/pubmed), a phenotype search on OMIM (https://www.omim.org), and reviewed clinical genetics panels from Prevention Genetics (Marshfield, WI), Fulgent (Temple City, CA), and The University of Chicago Genetic Services (Chicago, IL) to identify candidate genes for male infertility (Fig. 1).

Keywords for searching include: Male infertility, genetics of male infertility.

### Classification of genes based on pathophysiology

Based on the function and phenotype described in the peer reviewed literature, the genes were categorized into Early Development, Pre-testicular/Endocrine, Testicular, and Post-testicular/Obstructive. Pre-testicular/Endocrine, Testicular, and Post-testicular/Obstructive represent previously recognized etiological categories that mirror the clinical approach to male infertility [11]. Early Development refers to infertility that originated from defects in organogenesis or embryological development. Pre-testicular/Endocrine encompasses infertility caused by dysfunction of the endocrine regulation of spermatogenesis, including abnormalities related to the hypothalamic-pituitary axis and the testis [11]. Testicular represents infertility due to disorders related to spermatogenesis itself [11]. Post-testicular/Obstructive includes infertility secondary to ductal obstruction at any site in the male reproductive tract [11]. The Early Development category was created to capture genes associated with defects in organogenesis or embryological development, in which infertility is unlikely to respond to treatment in adulthood because the underlying abnormality precedes normal gonadal or reproductive tract formation. Examples include disorders of sex development and cryptorchidism. Differentiating among these etiological categories helps guide clinical decision making, optimizing patient counseling, and directing research efforts because the available medical, surgical, and assisted reproductive therapies differ depending on the pathophysiology [12].

### Grading of genes based on the genetic evidence

We reviewed the strength of evidence as stated in the literature or as stated by OMIM phenotype in order to classify genes by strong, moderate, or weak evidence (Fig. 1). Genes that were classified as having strong supporting evidence were confirmed by a definitive OMIM phenotype or by definitive classification by Houston et al. [2] (Fig. 1 - bright green box). Moderate evidence includes genes for which OMIM described variants of unknown significance or associations pending confirmation. Moderate evidence also encompasses genes that were either graded as moderate evidence by Houston et al. or as limited, no evidence, or unable to confirm with new associations in sequencing studies in the interim [2] (Fig. 1 - bright yellow box). Weak evidence encompasses genes that have been implicated in male infertility by only one human study, only by animal studies (and no human genetics studies), or only by pathophysiological reasoning (Fig. 1 - bright orange box).

### Gene ontology analysis

Gene Ontology (GO) [8] provides a structured framework for describing the relationships between genes based on their roles at different biological levels, including Molecular Function (MF), Biological Processes (BP), and Cellular Components (CC). We employed g: GOSt version e112_eg59_p19_25aa4782 (https://biit.cs.ut.ee/gprofiler/gost) [13], which is an algorithm to characterize genes with statistical enrichment analysis from a number of functional evidence data sources. We leverage it to identify overrepresented Gene Ontologies underlying the strong-evidence genes and to highlight driver terms, which are GO terms that are most enriched and non-redundant in the dataset [13]. The enrichment analysis was performed using an adjusted significance threshold of p-adjusted < 0.05 (p-adj < 0.05).

We utilized REVIGO version 1.8.1 [14], a tool that summarizes lists of GO terms by removing redundant entries based on semantic similarity, to illustrate a tag cloud of keywords that underline the Gene Ontologies which were overrepresented among the strong-evidence genes [14]. This provides a visualization of the semantic concepts that are key to the genetics of male infertility (Supplementary Fig. 1).

### Pathway analysis

Reactome pathways describe the relationships between genes based on the biochemical reactions and processes in which they participate, including metabolic pathways, signaling cascades, and other cellular mechanisms [9]. PANTHER (Protein ANalysis THrough Evolutionary Relationships) version 19.0 (https://pantherdb.org) [15], was utilized to perform pathway enrichment analysis, which identifies overrepresented Reactome pathways by comparing the selected genes to Homo sapiens reference gene set. This approach identifies pathways relevant to genetic causes of male infertility. Fisher’s exact test and Bonferroni correction for multiple testing were conducted. Significance was determined using a p-value threshold of < 0.05.

### Gene tissue expression analysis

We further utilized the GTExv8 Multi Gene Query (https://www.gtexportal.org/home/*)* [10] to examine tissue-specific gene expression using RNA sequencing data. The strong-evidence genes were analyzed within pathophysiological categories. The Post-testicular/Obstructive category was not included in this analysis as it only included two strong-evidence genes. We illustrated the results as gene-tissue heat maps, which provide complementary insights into the pathophysiology underlying genetic male infertility pathways.

## Results

### Characteristics of genes associated with male infertility

Following a review of the literature, we identified 596 candidate genes with strong, moderate, or weak evidence of association with male infertility. Of these, 178 genes were classified as having strong-evidence, representing a 71% increase compared to the 104 genes identified in 2020. Supplementary Table 1 contains a list of the genes with strong-evidence as highlighted in green. Supplementary Table 1 also contains a full list of candidate genes, including details of their categorization, function, phenotype, and relevant references to the literature.

### Enrichment analysis of strong-evidence candidate genes

**Fig. 2:**
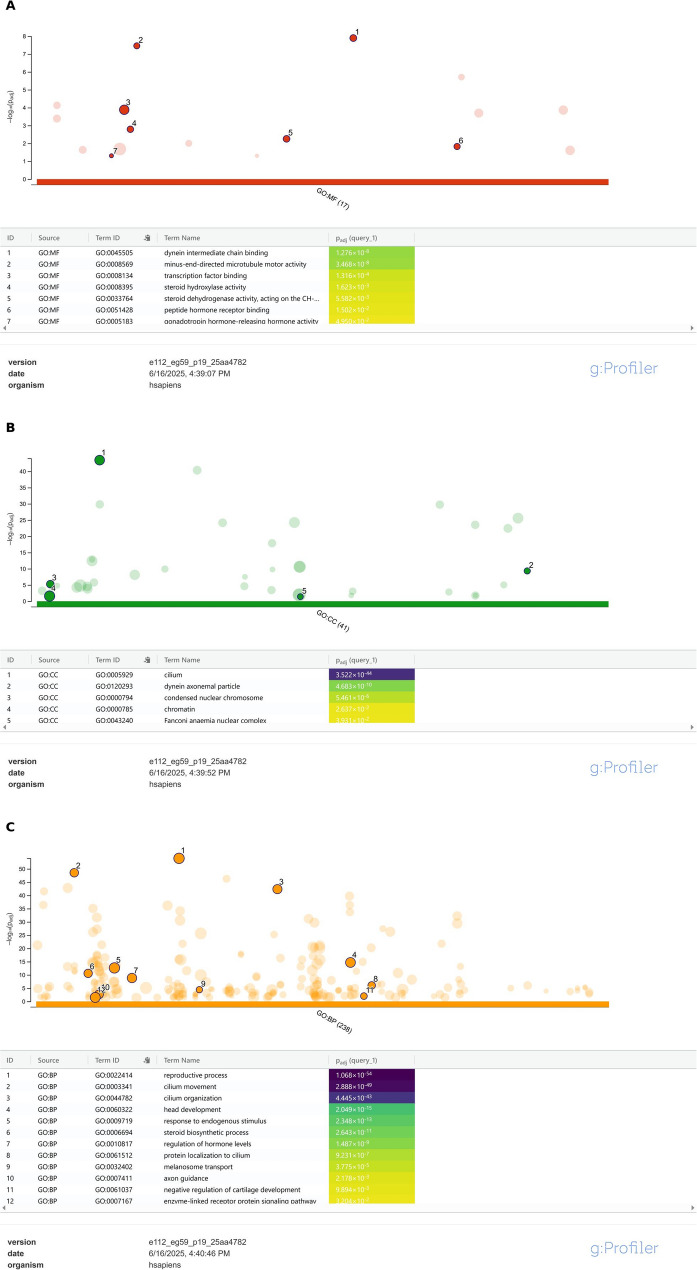
Overrepresented Gene Ontology terms among strong-evidence male infertility genes

We performed gene ontology overrepresentation analysis of candidate genes with strong-evidence (Supplementary Table 1), examining their associated molecular function (GO-MF), cellular component (GO-CC), and biological process (GO-BP). GO-MF analysis is presented as a Manhattan-like plot in Fig. 2A. The most significant driver terms were related to microtubule/ciliary function. Other significant terms were related to the metabolism and action of steroids and hormones. GO-CC analysis (Fig. 2B) revealed significant driver terms related to cilia/flagella, nuclear and chromosomal organization, and DNA repair (Fig. 2B). GO-BP analysis (Fig. 2C) identified driver terms related to ciliary/flagellar functions, steroid synthesis and metabolism, and neuronal development.

### Pathway analysis

Reactome pathway analysis via PANTHER revealed overrepresentation among the strong-evidence gene set of biological themes relating to hormonal action/regulation, ciliary function, and cell division.

### Gene tissue expression analysis of strong-evidence candidate genes

**Fig. 3:**
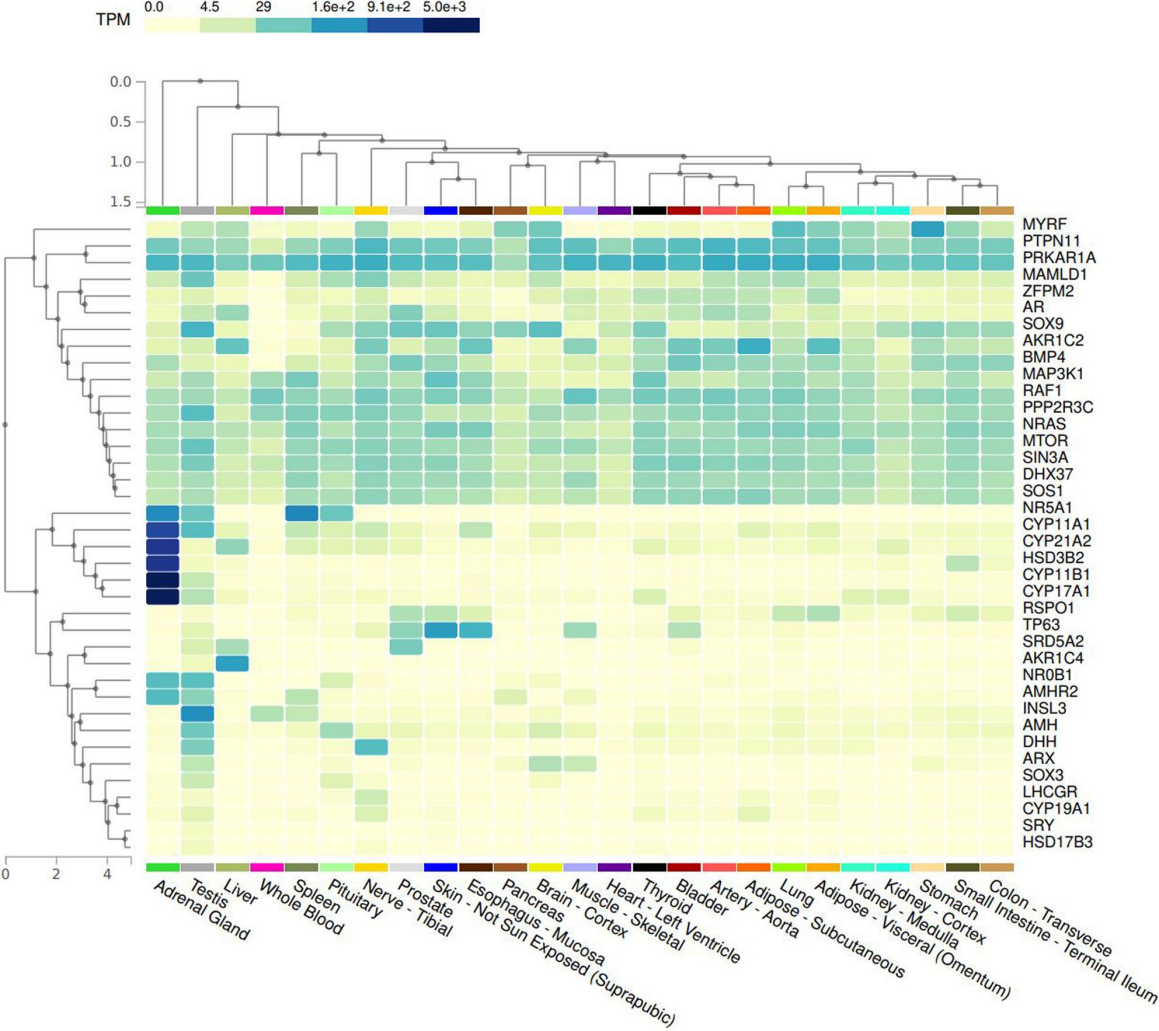
Early development gene-tissue expression heat map

Gene tissue expression of the strong-evidence genes (Supplementary Table 1) within each etiological category revealed several subgroups of tissue expression. The heat map in Fig. 3, which depicts the expression of strong-evidence genes within the developmental category, revealed diverse expression patterns, including a group strongly expressed in the adrenal gland (e.g. *CYP11A1*, *CYP21A2*, and *CYP17A1*), a group highly expressed across multiple tissues (e.g. *PTPN11* and *RAF1*), and a group moderately expressed across multiple tissues (e.g. *MTOR* and *DHX37*).

**Fig. 4:**
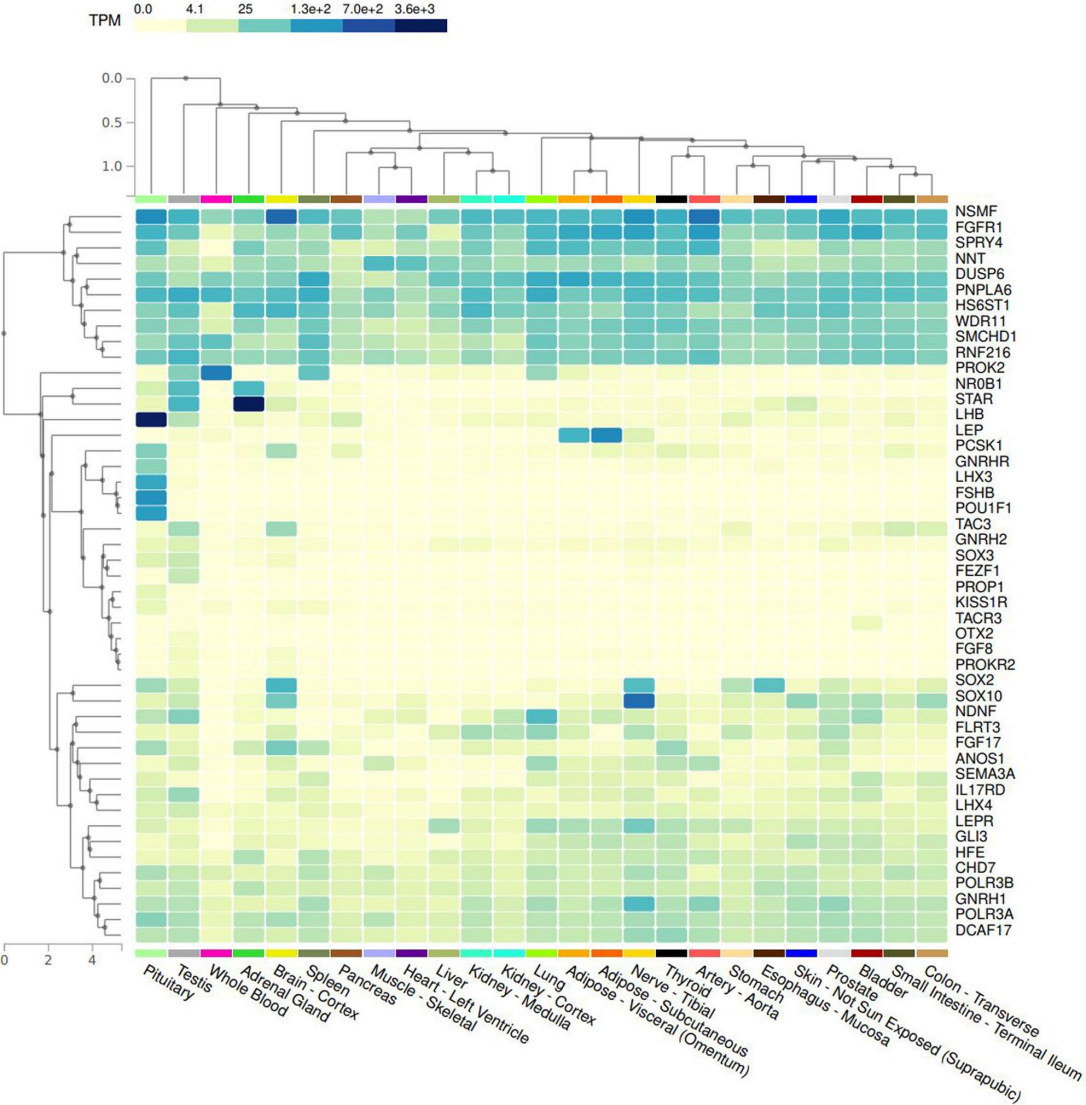
Pre-testicular/endocrine gene-tissue expression heat map

The next heat map in Fig. 4, which outlined the expression of strong-evidence genes within the endocrine category, demonstrated that most of these genes were expressed in the pituitary gland and hypothalamus. Cluster analysis revealed distinct expression patterns, with a group highly expressed across multiple tissues (e.g. *NSMF*, *FGFR1*, and *PNPLA6*), a group moderately expressed across multiple tissues (e.g. *POLR3A* and *CHD7*), and a group expressed in the testis and hypothalamus (e.g. *TAC3* and *SOX3*).

**Fig. 5:**
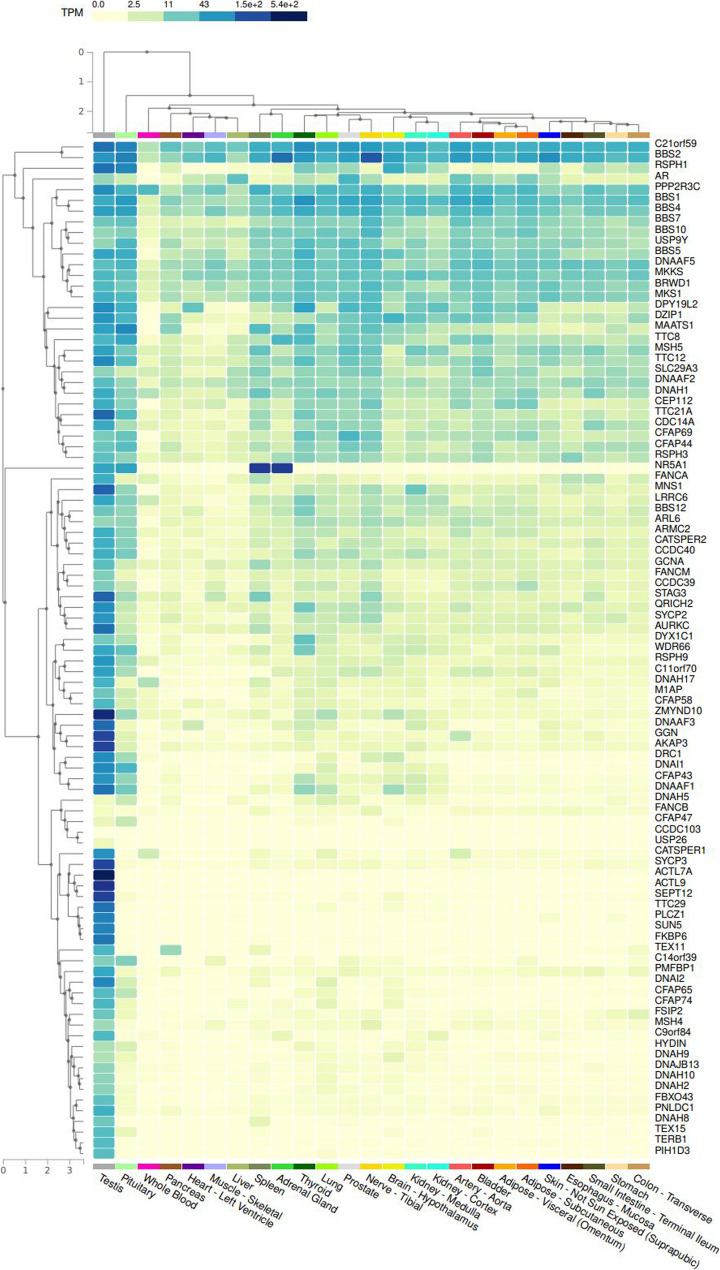
Testicular gene-tissue expression heat map

The heat map in Fig. 5, which summarizes the expression of strong-evidence genes in the testicular category, revealed that nearly all of the genes were expressed in the testis. Several patterns of tissue expression were identified by cluster analysis, with one group highly expressed across multiple tissues (e.g. *BBS1*, *USP9Y*, and *DNAAF5*), one group moderately expressed across multiple tissues (e.g. *DNAAF2*, *DNAH1*, and *CFAP44*), another group expressed only in the testis (e.g. *TEX15*, *TERB1*, and *ACTL9*) (Fig. 5).

## Discussion

### Systemic manifestations of male infertility

Next generation sequencing has led to the ongoing discovery of monogenic causes of male infertility [2, 16]. By using gene set analysis, pathway analysis, and gene-tissue expression analysis, we identified multisystem pathways that are overrepresented among the genes associated with male infertility. While the involvement of individual pathways such as ciliary function, endocrine regulation, and DNA repair in male infertility has been recognized in the context of specific genes or syndromes, our analysis provides a formal, quantitative demonstration of their overrepresentation across the full landscape of strong-evidence male infertility associated genes. This integrated perspective reveals the extent to which male infertility genes map to shared biological processes with systemic relevance. Our findings align with epidemiological data suggesting a link between male infertility and overall health status. Eisenberg et al. reported that 44% of men with infertility had at least one medical diagnosis unrelated to reproductive health [4] and higher Charlson Comorbidity Index (CCI) scores have been associated with lower semen parameters [4]. Specific comorbidities including endocrine, metabolic, cardiovascular, and cerebrovascular conditions have been reported in association with abnormal semen parameters [4, 17, 18]. Infertile men are also at increased risk for malignancies, autoimmune disorders, and mortality [19, 20]. Our pathway-level findings may provide a biological framework for understanding these clinical observations.

### Ciliopathies

Ciliary and flagellar biology was the most prominent theme across all three GO domains in our analyses: cilium in GO-CC (Fig. 2B), cilium movement and organization in GO-BP(Fig. 2C), and dynein intermediate chain binding and minus-end-directed microtubule motor activity in GO-MF(Fig. 2A). The Reactome analysis also reflected this pattern, with BBSome-mediated cargo-targeting to cilium among the most significantly overrepresented pathways (Table 1). The degree and consistency of enrichment across these analytical approaches underscore the central role of ciliary biology among the curated set of male infertility genes.

**Table 1:** Overrepresented reactome terms among the strong evidence genes bonferroni count: 2344

Reactome pathways	All Gene #	Male infertility Gene #	Expected #	Fold Enrichment	*P*-value
Transcriptional regulation of testis differentiation	11	6	0.1	61.01	4.91E-07
Mineralocorticoid biosynthesis	6	3	0.05	55.92	3.23E-02
Hormone ligand-binding receptors	13	6	0.12	51.62	1.80E-06
Androgen biosynthesis	11	5	0.1	50.84	5.61E-05
BBSome-mediated cargo-targeting to cilium	23	10	0.21	48.63	6.19E-12
Glucocorticoid biosynthesis	10	4	0.09	44.74	2.92E-03
Metabolism of steroid hormones	36	11	0.32	34.18	2.50E-11
Cargo trafficking to the periciliary membrane	51	10	0.46	21.93	5.57E-08
Metabolic disorders of biological oxidation enzymes )	34	5	0.3	16.45	2.86E-02
Metabolism of steroids	153	13	1.37	9.5	2.88E-06
Meiosis	85	7	0.76	9.21	2.62E-02
Reproduction	123	10	1.1	9.09	3.75E-04
Cilium Assembly	200	11	1.79	6.15	4.55E-03

The heatmap of the Testicular category of genes in Fig. 5 illustrates the dual role of cilia in both spermatogenesis and extra-gonadal physiology. The BBS genes (*BBS1*, *BBS2*, *BBS4*, *BBS5*, *BBS7*, *BBS10*, *BBS12*, *MKKS*) show the broadest expression (Fig. 5), with consistent expression across multiple tissues — a pattern consistent with the multisystem phenotype of Bardet-Biedl syndrome, including renal, retinal, metabolic, and neurological involvement [21, 22]. Several other ciliary genes, including *DNAAF5*, *LRRC6*, and *MKS1*, also demonstrate expression beyond the testis (Fig. 5). Taken together, these expression patterns suggest that ciliary gene variants associated with male infertility have broad systemic implications and that the specific gene involved may inform the scope of clinical evaluation.

Beyond canonical ciliary terms, melanosome transport also emerged as an enriched driver term in the GO-BP analysis (Fig. 2C). Melanosome transport is a microtubule-dependent process that shares motor protein machinery with ciliary transport, and its enrichment suggests that the role of microtubule biology in male infertility extends beyond ciliary structures alone.

### Fanconi anemia nuclear complex and DNA repair

Fanconi anemia (FA) nuclear complex (p-adj 3.931 × 10 − 2) was another overrepresented GO (Fig. 2B). The FA pathway is critical for DNA interstrand crosslink repair and genomic stability [23], and mouse studies have linked pathogenic variants in FA genes to impaired spermatogenesis through disruption of spermatogonial stem cell maintenance and meiotic progression [24–28]. Importantly, FA is a multisystem disorder associated with bone marrow failure, predisposition to multiple malignancies (hematologic, head and neck, skin, gynecologic, and gastrointestinal), and congenital anomalies including VACTERL-H (vertebral, anal, cardiac, tracheoesophageal fistula, esophageal/duodenal atresia, renal, limb, hydrocephalus) association [23, 29, 30]. The enrichment of this complex among male infertility genes underscores the need for evaluation beyond the genitourinary tract in men whose infertility is related to DNA repair pathway defects.

### Endocrine processes

Endocrine-related terms were consistently enriched across GO-MF, GO-BP, and Reactome analyses (Fig. 2A, 2C, Table 1), spanning steroid biosynthesis, hormone receptor binding, and gonadotropin-releasing hormone activity. Notably, Reactome analysis identified overrepresentation of mineralocorticoid and glucocorticoid biosynthesis alongside the more expected androgen biosynthesis pathway (Table 1), indicating that the endocrine basis of genetic male infertility extends beyond the hypothalamic-pituitary-gonadal axis to include adrenal steroidogenesis. The co-enrichment of these pathways is driven in part by genes such as *CYP21A2* and *CYP11B1*, which participate in multiple steroidogenic branches [33]. Defects in these enzymes can cause male infertility through the development of testicular adrenal rest tumors [34, 35], while simultaneously producing extra-genitourinary manifestations such as electrolyte disturbances, blood pressure abnormalities, and advanced bone age [34–37], reinforcing the need for multidisciplinary evaluation in men with genetic male infertility involving adrenal steroidogenic pathways.

Idiopathic hypogonadotropic hypogonadism (IHH) is a rare but clinically important and treatable cause of male infertility [32]. The prominence of this etiology in our gene set was supported by the inclusion of genes such as *KAL1*, *FGFR1*, *PROK2*, and *CHD7* in the strong-evidence gene list (Supplementary Table 1) and by the overrepresentation of related terms including peptide hormone receptor binding, gonadotropin hormone-releasing hormone activity, and axon guidance (Fig. 2A, 2C, Table 1). Many of these genes are also associated with non-reproductive features beyond infertility, including anosmia, cleft lip or palate, hearing impairment, renal agenesis, and skeletal anomalies, reflecting the developmental roles of the affected signaling pathways [31].

### Notable absences in enrichment results

Several biological processes with established roles in male fertility were not enriched among the strong-evidence gene set. No terms related to sperm capacitation reached significance in the GO-MF analysis. Interestingly, no terms relating to the acrosome, apoptosis, and oxidative stress were enriched despite their well-documented roles in spermatogenesis and sperm function [32–34]. Apoptosis is a key mechanism of germ cell quality control during spermatogenesis [32], and oxidative stress is a well-established contributor to sperm DNA damage and motility impairment [33]. These absences highlight the value of formal statistical enrichment testing in distinguishing pathways that are overrepresented within the known genetic architecture from those that may be assumed to be central based on prior knowledge alone.

### Limitations of the study

Several limitations of this study should be acknowledged. First, the gene set was curated from published literature, OMIM, and clinical genetics panels, which inherently favors well-studied and clinically recognized pathways. Consequently, genes that are associated with less characterized pathways, or recently discovered, may be underrepresented. As novel genes are identified and the evidence base expands, future analyses may reveal enrichment of different pathways.

Second, gene set enrichment analysis identifies statistical overrepresentation of pathway annotations but does not establish causal or clinical relationships. The observation that multisystem pathways are enriched in a gene set may guide the evaluation of individuals who are affected by variants in those genes, but does not confirm that affected individuals will manifest a corresponding systemic phenotype.

Third, the etiological classification of genes into categories (Early Development, Pre-testicular/Endocrine, Testicular, Post-testicular/Obstructive) relies on current understanding of gene function and previously documented phenotypes. These categories were designed to align with established clinical diagnostic frameworks and may evolve as new evidence emerges.

Finally, tissue expression data from GTEx represents population-level averages in adults without disease. Although this data is useful for inferring which organ systems may be affected when a gene is disrupted, the expression patterns do not capture developmental stages relevant to early-onset conditions and may not directly reflect gene expression in the tissues of men with infertility.

## Conclusion

By leveraging gene ontology, Reactome pathway analysis, and gene-tissue expression analysis to a curated set of 178 male infertility genes, we identified overrepresentation of ciliary/flagellar, endocrine, and DNA repair pathways, all of which have known roles in extra-gonadal organ systems.

These findings are consistent with epidemiological evidence linking male infertility to broader systemic health and support the implementation of multidisciplinary evaluation for men with infertility integrating expertise from urology, endocrinology, pulmonology, oncology, and medical genetics.

As genomic sequencing technologies continue to uncover novel loci, functional validation of candidate genes and further exploration of their extragenital effects are required. Expanding the genetic landscape of male infertility will enhance our understanding of reproductive pathophysiology and advance the potential for personalized diagnostics, therapies, and holistic patient care.

## Supplementary Information

Supplementary Material 1.

Supplementary Material 2.

## Data Availability

All data analyzed in this study are derived from published literature and publicly available bioinformatics resources. The curated list of male infertility associated genes, including evidence grading, etiologic categorization, functional notes, phenotypes, and supporting references, is provided in Supplementary Table 1. Summary results of the enrichment and expression analyses are presented in the main manuscript (tables/figures). No new human sequencing datasets were generated for this study. Any additional intermediate analysis outputs used to generate the results are available from the corresponding author upon reasonable request.

## Abbreviations

AZF: Azoospermia Factor
BBS: Bardet-Biedl syndrome
BP: Biological Process
CCI: Charlson Comorbidity Index
CC: Cellular Component
FA: Fanconi anemia
GnRH: Gonadotropin-releasing hormone
GO: Gene Ontology
GTEx: Genotype-Tissue Expression
IFT: Intraflagellar transport
IHH: Idiopathic hypogonadotropic hypogonadism
MF: Molecular Function
NGS: Next-generation sequencing
OMIM: Online Mendelian Inheritance in Man
p-adj: Adjusted p-value
PANTHER: Protein ANalysis THrough Evolutionary Relationships
TART: Testicular adrenal rest tumors
TPM: Transcripts per million
VACTERL-H: Vertebral, anal, cardiac, tracheoesophageal fistula, esophageal/duodenal atresia, renal, limb, hydrocephalus
Yq: Long arm of the Y chromosome
