# Supplementary material for: Gene set enrichment analysis of curated monogenic loci highlights key pathways and multisystem involvement in male infertility
Source: Basic Clin Androl. 2026 Jun 10;36:17. doi: 10.1186/s12610-026-00316-2 (PMC13251197; doi:10.1186/s12610-026-00316-2)
Supplement: Supplementary file 1 — Supplementary Material 1. [file 12610_2026_316_MOESM1_ESM.docx]

Supplementary table 1: Curated list of candidate genes with their associated phenotypic and indexing information. References listed as PMIDs. OMIM - Online Mendelian Inheritance in Man. GU - Genitourinary. AR - Autosomal recessive. AD - Autosomal dominant. SMu - Spontaneous mutation. XL - X-linked. XLR - X-linked recessive. XLD - X-linked dominant. NOA - Non-obstructive azoospermia. OA - Obstructive azoospermia. Green - strong evidence. Yellow - moderate evidence. Orange - weak evidence. Cyan - subcategorization for Pretesticular/Endocrine genes. Green background - Strong evidence. Yellow background - Moderate evidence. Organge background - Weak evidence.

| Gene symbol | Locus | OMIM | Inheritance | Phenotype MIM | GU (or endocrine)  Phenotype(s) | Other associated  phenotypes | Reference(s) |
| --- | --- | --- | --- | --- | --- | --- | --- |
| *AKR1C2* | 10p15.1 | 600450 | AR | 614279 | 46XY sex reversal 8 |  | 24986825; 32732174; 21802064; 24793988; 10998348 |
| *AKR1C4* | 10p15.1 | 600451 | AR | 614279 | {46XY sex reversal 8, modifier of} |  | 24986825; 30496128; 32732174; 10998348 |
|  |  |  |  |  |  | {Craniosynostosis |  |
|  |  |  |  |  |  | 5, susceptibility |  |
|  |  |  |  |  |  | to} |  |
|  |  |  | AD | 615529 |  | Frontonasal |  |
|  |  |  | AR | 613451 | Cryptorchidism | dysplasia 2 |  |
|  |  |  | AD | 609597 | per Houston et. | Parietal foramina |  |
| ALX4 | 11p11.2 | 605420 | AD | NA - PS219050 | al. 2020 | 2 | 32385972 |
| AMH | 19p13.3 | 600957 | AR | 261550 | Persistent Mullerian duct syndrome, type I | inguinal hernia | 28742509;28142151;26492835;26181047;25026127;23611722;23295284;22797409;22188863;11760020;8162013;1483695;2023927;28938747;30496128;31377750;31291191;31291191;  31238341 |
| AMHR2 | 12q13.3 | 600956 | AR | 261550 | Persistent Mullerian duct syndrome, type II | inguinal hernia | 29687786;29332065;29285121;28142151;28094762;28163853;25383892;23295284;22584735;20480734;19457927;14745940;11549681;8872466;7493017;31184456;30853106;30933950 |
|  |  |  |  |  | Androgen |  |  |
|  |  |  |  |  | insensitivity |  |  |
|  |  |  |  |  | Androgen |  |  |
|  |  |  |  |  | insensitivity, |  |  |
|  |  |  |  |  | partial, with or |  | 29264534;28659371;27498682;26681172;25677139;25532578;25433660;23808476;22469007;21962961;21127309;20305676;20150575;19937586;19361789;19017913;17054461;16400085; |
|  |  |  |  |  | without breast |  | 14756668;12843171;12801573;11788673;11545293;11422119;10999818;10976396;10946887;10935543;10852459;10470409;10359561;10333364;9851768;9788719;9607727;9433918;7926115; |
|  |  |  |  |  | cancer |  | 7993455;1424203;1598912;1750490;29396419;29267169;29051026;29393262;29278518;28261839;27849622;27403927;27087292;27051040;26935236;26688387;26352087;26778393;26492835; |
|  |  |  |  |  | Hypospadias 1, |  | 26197461;25605705;25241384;24907477;24737579;24577144;24367986;23729601;23589523;22334658;21535007;20056211;20333878;19851057;19601939;18697867;18656523;17937062; |
|  |  |  |  |  | X-linked |  | 16448442;24790336;16283146;16151980;15963062;15925895;15522944;15486055;15541764;15452386;15266301;15171708;15053245;15001585;14646391;19125473;12713250;12705360; |
|  |  |  | XLR | 300068 | Spinal and bulbar |  | 12068007;12050225;12006704;11818512;11807912;11701709;11591424;11587068;11579211;11549642;11397856;11260228;11238515;12058191;10999816;10971094;10690872;10590024; |
|  |  |  | XLR | 312300 | muscular atrophy |  | 10571951;10543676;10502786;10458483;10092153;10022458;9856504;9768671;9720578;9627582;9543136;9519369;9360511;9345099;9302173;9245853;9106550;9160185;9156036;9010714; |
|  |  |  | XLR | 300633 | of Kennedy |  | 9039340;9001799;8823308;8768864;8683794;8809734;8647313;8824883;7673412;7581399;7929841;7970939;8033918;8128958;8126121;8246999;8274427;8325932;8450042;8097257;8446106; |
|  |  |  | XLR | 313200 | {Prostate cancer, |  | 1307250;1303262;1316540;1356901;1307741;2001749;29785970;30496128;30411392;30251955;32155011;31377750;31373714;31219235;30815925;30601762;30372699;29582157;32469048; |
| AR | Xq12 | 313700 | AD, SMu | 176807 | susceptibility to} |  | 32435981;32345305;32242295;32229106 |
|  |  |  |  |  | Hydranencephaly |  |  |
|  |  |  |  |  | with abnormal |  |  |
|  |  |  |  |  | genatalia |  |  |
|  |  |  |  |  | (ambiguous |  |  |
|  |  |  |  |  | genitalia, small |  |  |
|  |  |  |  |  | testes, |  |  |
|  |  |  |  |  | underdeveloped |  |  |
|  |  |  |  |  | scrotal folds, |  |  |
|  |  |  |  |  | small penis) |  |  |
|  |  |  |  |  | Lyssencephaly, |  |  |
|  |  |  |  |  | X-Linked, 2 | Developmental |  |
|  |  |  |  |  | (ambiguous | and epileptic |  |
|  |  |  |  |  | genitalia, small | encephalopathy 1 |  |
|  |  |  |  |  | testes, | Hydranencephaly |  |
|  |  |  |  |  | underdeveloped | with abnormal |  |
|  |  |  |  |  | scrotal folds, | genitalia |  |
|  |  |  |  |  | small penis) | Intellectual |  |
|  |  |  |  |  |  | developmental |  |
|  |  |  |  |  |  | disorder, X-linked |  |
|  |  |  | XLR | 308350 | Proud syndrome | 29 |  |
|  |  |  | XL | 300215 | (cryptorchidism, | Lissencephaly, X- |  |
|  |  |  | XLR | 300419 | hypospadias, | linked 2 |  |
|  |  |  | XL | 300215 | small kidney | Partington |  |
|  |  |  | XLR | 309510 | (reported in 1 | syndrome |  |
| ARX | Xp21.3 | 300382 | XL | 300004 | patient)) | Proud syndrome | 33742552 |
| BMP4 | 14q22.2 | 112262 | AD | 607932  600625 | Microphthalmia, syndromic 6 (cryptorchidism, micropenis, small scrotum, bifid scrotum, hypoplastic foreskin, hypospadias, hypoplastic kidneys, hypoplastic adrenal glands) | Orofacial cleft 11 | 17003840;31219235;29582157;34009138;33270637 |

Supplementary table 1: Curated list of candidate genes with their associated phenotypic and indexing information. References listed as PMIDs. OMIM - Online Mendelian Inheritance in Man. GU - Genitourinary. AR - Autosomal recessive. AD - Autosomal dominant. SMu - Spontaneous mutation. XL - X-linked. XLR - X-linked recessive. XLD - X-linked dominant. NOA - Non-obstructive azoospermia. OA - Obstructive azoospermia. Green - strong evidence. Yellow - moderate evidence. Orange - weak evidence. Cyan - subcategorization for Pretesticular/Endocrine genes. Green background - Strong evidence. Yellow background - Moderate evidence. Organge background - Weak evidence.

| Gene symbol | Locus | OMIM | Inheritance | Phenotype MIM | GU (or endocrine)  Phenotype(s) | Other associated  phenotypes | Reference(s) |
| --- | --- | --- | --- | --- | --- | --- | --- |
|  |  |  |  |  |  | Adenocarcinoma |  |
|  |  |  |  |  |  | of lung, somatic |  |
|  |  |  |  |  |  | Cardiofasciocutan |  |
|  |  |  |  |  |  | eous syndrome |  |
|  |  |  |  |  |  | Colorectal cancer, |  |
|  |  |  |  |  |  | somatic |  |
|  |  |  |  |  |  | LEOPARD |  |
|  |  |  |  |  |  | syndrome 3 |  |
|  |  |  |  | 211980 |  | Melanoma, |  |
|  |  |  | AD | 115150 |  | malignant, |  |
|  |  |  |  | 114500 |  | somatic |  |
|  |  |  | AD | 613707 |  | Nonsmall cell lung |  |
|  |  |  |  | 155600 |  | cancer, somatic |  |
|  |  |  |  | 211980 | Cryptorchidism | Noonan |  |
|  |  |  | AD | 613706 | per Houston et. | syundrome 7 |  |
| BRAF | 7q34 | 164757 | AD | NA - PS219050 | al. 2020 |  | 29752777 |
| CALCA | 11p15.2 | 114130 | Undetermin ed | NA - PS219050 | Cryptorchidism per Houston et. al. 2020 |  | 15636430 |
| CALCB | 11p15.2 | 114160 | Undetermin ed | NA - PS219050 | Cryptorchidism per Houston et. al. 2020 |  | 15636430 |
|  |  |  |  |  | Cryptorchidism - |  |  |
|  |  |  | AR | 618773 | per Houston et. | ?Lymphatc |  |
| CALCRL | 2q32.1 | 114190 | AR | NA - PS219050 | al. 2020 | malformation 8? | 15636430 |
| CBX2 | 17q25.3 | 602770 | AR | ?613080? | ?46XY sex reversal 5? female: normal female external geitalia, normal vagina, normal uterus, normal ovaries with primordial follicles, |  | 23219007;30496128;19361780 |
| CBX2 | 17q25.3 | 602770 | AR | NA | 46,XX Disorder of sex development per Houston et. al. 2020 |  | 23219007;30496128;19361780 |
| CRCP | 7q11.21 | 606121 | Undetermin ed | NA - PS219050 | Cryptorchidism - per Houston et. al. 2020 |  | 15636430 |
| DHH | 12q13.12 | 605423 | AR | 233420 | 46XY sex reversal 7 | neuropathy | 27711951;30298535; 11017805; 15356051; 25927242; 28589169; 28708305; 29471294 |
| DHH | 12q13.12 | 605423 | AR | 607080 | 46XY gonadal dysgenesis with minifasicular neuropathy | neuropathy | 27711951;30298535; 11017805; 15356051; 25927242; 28589169; 28708305; 29471294 |
|  |  |  |  |  |  | Neurodevelopme |  |
|  |  |  |  |  |  | ntal disorder with |  |
|  |  |  |  |  |  | brain anomalies |  |
|  |  |  |  |  |  | and with or |  |
|  |  |  |  |  | 46XY sex reversal | without vertebral |  |
|  |  |  | AD | 273250 | 11 | or cardiac |  |
| DHX37 | 12q24.31 | 617362 | AR | 618731 |  | anomalies | 31337883;31287541;37717579;37240737;37147882;37065748;36617173;35835064;35432193;31745530 |
| DMRT2 | 9q24.3 | 604935 |  | none confirmed | none reported | severe rib and vertebral malformations, not confirmed | 15481033, 10999792 |
|  |  |  |  |  |  | {Migraine, |  |
|  |  |  |  |  |  | susceptibility to} |  |
|  |  |  |  |  |  | {Myocardial |  |
|  |  |  |  |  |  | infarction, |  |
|  |  |  | AD | 157300 |  | susceptibility to} Breast cancer, |  |
|  |  |  |  | 608446 |  | somatic |  |
|  |  |  |  | 114480 | 46,XY Disorders | Estrogen |  |
| ESR1 | 6q25.1-25.2 | 133430 | AR  AD | 615363  NA | of sex  development | resistance | 16213843;29582157;37010083 |
| FGF9 | 13q12.11 | 600921 | AD AD | 612961  NA | 46,XY Disorders of Sex Development | Multiple synostoses syndrome 3 | 26260363;23380608;32452519;36349847 |

Supplementary table 1: Curated list of candidate genes with their associated phenotypic and indexing information. References listed as PMIDs. OMIM - Online Mendelian Inheritance in Man. GU - Genitourinary. AR - Autosomal recessive. AD - Autosomal dominant. SMu - Spontaneous mutation. XL - X-linked. XLR - X-linked recessive. XLD - X-linked dominant. NOA - Non-obstructive azoospermia. OA - Obstructive azoospermia. Green - strong evidence. Yellow - moderate evidence. Orange - weak evidence. Cyan - subcategorization for Pretesticular/Endocrine genes. Green background - Strong evidence. Yellow background - Moderate evidence. Organge background - Weak evidence.

| Gene symbol | Locus | OMIM | Inheritance | Phenotype MIM | GU (or endocrine)  Phenotype(s) | Other associated  phenotypes | Reference(s) |
| --- | --- | --- | --- | --- | --- | --- | --- |
|  |  |  |  |  |  | Encephalocranioc |  |
|  |  |  |  |  |  | utaneous |  |
|  |  |  |  |  |  | lipomatosis, |  |
|  |  |  |  |  |  | somatic mosaic |  |
|  |  |  |  |  |  | Hartsfield |  |
|  |  |  | AD | 613001 |  | syndrome |  |
|  |  |  | AD, AR, dig, | 615465 |  |  |  |
|  |  |  | olig | 147950 |  | Jackson-Weiss |  |
|  |  |  | AD | 123150 |  | syndrome | 25739677;22035731;17200176;16764984;15845591;15613419;29658329;29228280;28833369;28411082;28209183;28195315;28087897;28008864;26277103;26199944;26051373;26031747; |
|  |  |  | AD | 166250 |  | Osteoglophonic | 25636053;25394172;25383892;25064402;24732674;24522099;24204987;23643382;23533228;23200691;23154428;22724017;21682876;21664240;21292259;20591981;20536592;19820032; |
|  |  |  | AD | 101600 |  | dysplasia | 18723471;18463157;18160472;17235395;17154279;16882753;16757108;16606836;16418210;16061567;15605412;15001591;12627230;30098700;31996231;31996231;31910188;31748124; |
|  |  |  | AD | 190440 | Isolated | Pfeiffer syndrome | 31748124;31781422;31200363;31200363;30669598;30669598;32485746;32485746;37814704;37805574;36859276;36407308;36384729;36138264;35669683;35668409;35457241;35133534; |
| FGFR1 | 8p11.23 | 136350 |  | NA - PS219050 | cryptorchidism | Trigonocephaly 1 | 34348883;33983622;33775534;33819414;33680884 |
|  |  |  |  |  |  | ?FG syndrome 2? |  |
|  |  |  |  |  |  | Cardiac valvular |  |
|  |  |  |  |  |  | dysplasia, X- |  |
|  |  |  |  |  |  | linked |  |
|  |  |  |  |  |  | Congenital short |  |
|  |  |  |  |  |  | bowel syndrome |  |
|  |  |  |  |  |  | Frontometaphyse |  |
|  |  |  |  |  |  | al dysplasia 1 |  |
|  |  |  |  |  |  | Heterotopia, |  |
|  |  |  |  |  |  | periventricular, 1 |  |
|  |  |  |  |  |  | Intestinal |  |
|  |  |  | XL | 300321 |  | pseudoobstructio |  |
|  |  |  | XL | 314400 |  | n, neuronal |  |
|  |  |  | XLR | 300048 |  | Melnick-Needles |  |
|  |  |  | XLR | 305620 |  | syndrome |  |
|  |  |  | XLD | 300049 |  | Otopalatodigital |  |
|  |  |  | XLR | 300048 |  | syndrome, type I |  |
|  |  |  | XLD | 309350 |  | Otopalatodigital |  |
|  |  |  | XLD | 311300 |  | syndrome, type II |  |
|  |  |  | XLD | 304120 | Androgen | Terminal osseous |  |
|  |  |  | XLD | 300244 | receptor | dysplasia |  |
| FLNA | Xq28 | 300017 | XL | NA | dysfunction |  | 28432720;17632775;15249610;33424767;36830778 |
|  |  |  |  |  | ?Testicular |  |  |
|  |  |  |  |  | anomalies with or | Atrial septal |  |
|  |  |  |  |  | without congenital | defect 2 |  |
|  |  |  | AD | ?615542? | heart disease? | Atrioventricular |  |
|  |  |  | AD | 607941 |  | septal defect 4 |  |
|  |  |  | AD | 614430 |  | Tetralogy of Fallot |  |
|  |  |  | AD | 187500 |  | Ventricular septal |  |
| GATA4 | 8p23.1 | 600576 | AD | 614429 |  | defect 1 | 27899089;29670578;21220346;29735817;37628683;35751412;35432193;34355877;32992319;31962012 |
| HOXA10 | 7p15.2 | 142957 | Undetermin ed | NA - PS219050 | Cryptorchidism - per Houston et. al. 2020 |  | 17216618;14960020;27108669;10037424 |
|  |  |  |  |  |  | Radioulnar |  |
|  |  |  |  |  |  | synostosis with |  |
|  |  |  |  |  |  | amegakaryocytic |  |
|  |  |  | AD |  | Cryptorchidism - | thrombocytopenia |  |
|  |  |  | Undetermin | 605432 | per Houston et. | 1 |  |
| HOXA11 | 7p15.2 | 142958 | ed | NA - PS219050 | al. 2020 |  | 17216618;32761594;28261830 |
|  |  |  |  |  | Preaxial |  |  |
|  |  |  |  |  | deficiency, |  |  |
|  |  |  |  |  | postaxial |  |  |
|  |  |  |  |  | polydactyly, and |  |  |
|  |  |  |  |  | hypospadias |  |  |
|  |  |  |  |  | Hand-Foot- |  |  |
|  |  |  | AD |  | Genital syndrome  Cryptorchidism - |  |  |
|  |  |  | AD |  | per Houston et. |  |  |
| HOXA13 | 7p15.2 | 142959 | AD | NA - PS219050 | al. 2020 |  | 17216618;14675924;30496128;10839976;8484413;32761594;27649277 |
| HOXA4 | 7p15.2 | 142953 | Undetermin ed | NA - PS219050 | Cryptorchidism - per Houston et. al. 2020 |  | 17003840;32761594 |
| HOXA9 | 7p15.2 | 142956 | Undetermin ed | NA - PS219050 | Cryptorchidism - per Houston et. al. 2020 |  | 17216618 |
| HOXB13 | 17q21.32 | 604607 | AR | 610997  NA - PS219050 | Cryptorchidism - per Houston et. al. 2020 | {Prostate cancer, hereditar, 9} | 17216618 |
| HOXB9 | 17q21.32 | 142964 | Undetermin ed | NA - PS219050 | Cryptorchidism - per Houston et. al. 2020 |  | 17216618 |
| HOXC10 | 12q13.13 | 605560 | Undetermin ed | NA - PS219050 | Cryptorchidism - per Houston et. al. 2020 |  | 17216618 |
| HOXC11 | 12q13.13 | 605559 | Undetermin ed | NA - PS219050 | Cryptorchidism - per Houston et. al. 2020 |  | 17216618 |

Supplementary table 1: Curated list of candidate genes with their associated phenotypic and indexing information. References listed as PMIDs. OMIM - Online Mendelian Inheritance in Man. GU - Genitourinary. AR - Autosomal recessive. AD - Autosomal dominant. SMu - Spontaneous mutation. XL - X-linked. XLR - X-linked recessive. XLD - X-linked dominant. NOA - Non-obstructive azoospermia. OA - Obstructive azoospermia. Green - strong evidence. Yellow - moderate evidence. Orange - weak evidence. Cyan - subcategorization for Pretesticular/Endocrine genes. Green background - Strong evidence. Yellow background - Moderate evidence. Organge background - Weak evidence.

| Gene symbol | Locus | OMIM | Inheritance | Phenotype MIM | GU (or endocrine)  Phenotype(s) | Other associated  phenotypes | Reference(s) |
| --- | --- | --- | --- | --- | --- | --- | --- |
| HOXC12 | 12q13.13 | 142975 | Undetermin ed | NA - PS219050 | Cryptorchidism - per Houston et. al. 2020 |  | 17216618 |
| HOXC13 | 12q13.13 | 142976 | AR  Undetermin ed | 614931  NA - PS219050 | Cryptorchidism - per Houston et. al. 2020 | Ectodermal dysplasia 9, hair/nail type | 17216618 |
| HOXC9 | 12q13.13 | 142971 | Undetermin ed | NA - PS219050 | Cryptorchidism -  per Houston et. al. 2020 |  | 17216618 |
| HOXD10 | 2q31.1 | 142984 | 192950  192950  Undetermin ed | AD AD  NA - PS219050 | Cryptorchidism - per Houston et. al. 2020 | Charcot-Marie-Tooth disease, foot deformity of Vertical talus, congenital | 17216618 |
| HOXD11 | 2q31.1 | 142986 | Undetermin ed | NA - PS219050 | Cryptorchidism -  per Houston et. al. 2020 |  | 17216618 |
| HOXD12 | 2q31.1 | 142988 | Undetermin ed | NA - PS219050 | Cryptorchidism -  per Houston et. al. 2020 |  | 17216618 |
| HOXD13 | 2q31.1 | 142989 | AD AD AD AD AD | 610713  113200  113300  186300  186000  NA - PS219050 | Cryptorchidism - per Houston et. al. 2020 | ?Brachydactyl-syndactly syndrome?  Brachydactyly, type D Brachydactyly, type E Syndactyly, type V  Synpolydactyly 1 | 17216618 |
| HOXD9 | 2q31.1 | 142982 | Undetermin ed | NA - PS219050 | Cryptorchidism - per Houston et. al. 2020 |  | 17216618;30478911 |
| HRAS | 11p15.5 | 190020 | AD AD  AD | NA - PS219050 | Bladder cancer, somatic  Cryptorchidism - per Houston et. al. 2020 | Congenital myopathy with excess of muscle spindles  Costello syndrome  Nevus sebaceous or wooly hair nevus, somatic Schimmelpenning  -Feuerstein-Mims syndrome, somatic mosaic Spitz nevus or nevus spilus, somatic  Thyroid carcinoma, follicular, somatic | 29752777 |
| HSD17B3 | 9q22.32 | 605573 | AR | 264300 | Pseudohermaphr oditism, male, with gynecomastia |  | 17466011;22445608;26681172;25960839;25605705;27073926;30496128;37855374;36617173;36563763;36110220;36077423;35588601;35432193;35065919;34009138;30668521;32865613 |
| HSD3B2 | 1p12 | 613890 | AR | 201810 | Adrenal hyperplasia, congenital, due to 3-beta-hydroxylase dehydrogenase 2 deficiency |  | 25605705;14764821;10770215;27476613;28870780;18252794;35432193;36553457;36233635;35757411;35714169;34055358;33757164;33864926 |
| INSL3 | 19q13.11 | 146738 | AD,  frequently maternally inherited | 219050 | Cryptorchidism (normozoospermi c or oligozoospermic) | gubernaculum feminization during embryogenesis in mice deficient in  INSL3 | 26840636;21853106;19017913;16687567;15353080;12970298;11182749;25728210;19416190;17437853;17028442;15579790;15533513;12601553;11992081;11746019;11383919;11380919;  11095425;10729310;31444964;37208861 |
| KISS1 | 1q32.1 | 603286 | AR | 614842 | Association pending confirmation - central precocious  puberty |  | 27544332;25739677;26199944;26031747;25783047;25636053;25064402;22724017;20237166;17179725 |

Supplementary table 1: Curated list of candidate genes with their associated phenotypic and indexing information. References listed as PMIDs. OMIM - Online Mendelian Inheritance in Man. GU - Genitourinary. AR - Autosomal recessive. AD - Autosomal dominant. SMu - Spontaneous mutation. XL - X-linked. XLR - X-linked recessive. XLD - X-linked dominant. NOA - Non-obstructive azoospermia. OA - Obstructive azoospermia. Green - strong evidence. Yellow - moderate evidence. Orange - weak evidence. Cyan - subcategorization for Pretesticular/Endocrine genes. Green background - Strong evidence. Yellow background - Moderate evidence. Organge background - Weak evidence.

| Gene symbol | Locus | OMIM | Inheritance | Phenotype MIM | GU (or endocrine)  Phenotype(s) | Other associated  phenotypes | Reference(s) |
| --- | --- | --- | --- | --- | --- | --- | --- |
| KRAS | 12p12.1 |  | AD  AD  AD AD | 108010  109800  114480  615278  613659  601626  211980  609942  600268  260350  614470  163200  NA - PS219050 | Bladder cancer, somatic  Noonan syndrome 3  Cryptorchidism per Houston et. al. 2020 |  | 29752777 |
| LHCGR | 2p16.3 | 152790 | AR AR AR | 238320  238320  238320 | Leydig cell hypoplasia with hypergonadotropi c hypogonadism Leydig cell hypoplasia with pseudohermaphro ditism  Luteinizing hormone  resistance, female |  | 23884663;18508780;9626653;26246498;23232123;14689055;10852464;9215288;11849253;8843415;27532428;26831561;21490077;21060208;19492585;16918965;16759041;14510919;  11936470;11200941;11134146;10084607;9973550;9661624;9598734;9467560;8855841;8812739;8607787;8929952;8829636;7593421;7629248;7562970;7628171;7714085;7892197;7757065;  7527413;8281137;7692306;9703386;10580072;10704433;29582157;34950567;34338568;32761586;30444213;29305568;37827240;29912377 |
| LHCGR | 2p16.3 | 152790 | AD | 176410  176410 | Leydig cell adenoma, somatic, with precocious puberty Precocious puberty, male |  | 23884663;18508780;9626653;26246498;23232123;14689055;10852464;9215288;11849253;8843415;27532428;26831561;21490077;21060208;19492585;16918965;16759041;14510919;  11936470;11200941;11134146;10084607;9973550;9661624;9598734;9467560;8855841;8812739;8607787;8929952;8829636;7593421;7629248;7562970;7628171;7714085;7892197;7757065;  7527413;8281137;7692306;9703386;10580072;10704433;29582157;34950567;34338568;32761586;30444213;29305568;37827240;29912377 |
| LHX9 | 1q31.3 | 606066 | AD | NA - PS400044 | 46,XY disorders of sex development per Houston et. al.  2020 |  | 11397841, 32949097 |
| MAMLD1 | Xq28 | 300120 | XLR | 300758 | Hypospadias 2, X-linked (46XY DSD) | n.r. | 21853106;27383042;26580071;25833151;25383892;22479329;20347055;18635673;24790369;17086185;29582157;35837313;33468338;33424767;33312738;32690052;31555317;30668521 |
| MAP2K1 | 15q22.31 | 176872 | AD AD | 615279  155950  NA - PS219050 | Cardiofaciocutane ous syndrome 3 (cryptorchidism in some patients)  Cryptorchidism - per Houston et. al. 2020 | Melorheostosis, isolated, somatic mosaic | 29752777;36313893 |
| MAP2K2 | 19p13.3 | 601263 | AD AD | 615280  NA - PS219050 | Cardiofaciocutane ous syndrome 4 (penoscrotal inversion) Cryptorchidism - per Houston et. al. 2020 |  | 29752777 |
| MAP3K1 | 5q11.2 | 600982 | AD | 613762 | 46 XY sex  reversal 6 | increased height in females, gonadoblastoma, dysgerminoma, sparse axillary and pubic hair,  hirsutism (rare) | 25383892;21129722;28504475 |
| MTOR | 1p36.22 | 601231 | AD | 607341  616638 | Smith-Kingsmore syndrome (cryptorchidism) |  | Smith, L. D., Saunders, C. J., Dinwiddie, D. L., Atherton, A. M., Miller, N. A., Soden, S. E., Farrow, E. G., Abdelmoity, A. T. G., Kingsmore, S. F. Exome sequencing reveals de novo germline mutation of mammalian target of rapamycin (MTOR) in a patient with megalencephaly and intractable seizures. J. Genomes Exomes 2: 63-72, 2013.  25851998, 26542245, 27159400, 27830187 |
| MYRF | 11q12.2 | 608329 | AD AD | 618280  618113 | Cardiac-urogenital syndrome (46XY DSD) | Encephalitis/ence phalopathy, mild, with reversible myelin vacuolization | 30985895;29446546;30070761;30532227;37511771;37147882;36467480;35432193 |
| NR0B1 (DAX1) | Xp21.2 | 300473 | XL | 300018 | 46XY sex reversal 2, dosage-sensitive |  | 28741070;27648561;26260363;26207377;25529318;23384712;21227944;16645015;16556678;16275267;11788621;10675358;29176027;28924487;28284037;28075027;27711951;27035099;  26537215;26030781;25993682;25968435;25064402;25003377;24197767;24232823;24140641;23585174;23295288;23018754;22761912;22562240;21925982;21632081;21270512;20975255;  21340153;20573681;19508677;19773398;19672728;19129717;18607630;18604556;18941128;18380948;18202527;18056774;17054473;16553032;16355812;15884018;15860922;15841486;  14689055;14689056;12773801;12629128;11443184;11113848;10931108;10599709;10599708;10522996;10361383;10341858;10022408;9508067;9415399;9195207;9003500;8855822;8770879;  8675564;8636263;8844218;7990958;28483799;30179867;30129976;32129306;32028936;31917682;31700544;31642359;31377750;31280422;31219797;30620004;32166680;35849255;35432221;  32860660;37237297;37118935;36160878;37906859;35848959;35784540;35417110;35230670;34938333;34373561;34130666;33381670;32460754;32129306;32028936; 31280422;31219797;  31164167 |

Supplementary table 1: Curated list of candidate genes with their associated phenotypic and indexing information. References listed as PMIDs. OMIM - Online Mendelian Inheritance in Man. GU - Genitourinary. AR - Autosomal recessive. AD - Autosomal dominant. SMu - Spontaneous mutation. XL - X-linked. XLR - X-linked recessive. XLD - X-linked dominant. NOA - Non-obstructive azoospermia. OA - Obstructive azoospermia. Green - strong evidence. Yellow - moderate evidence. Orange - weak evidence. Cyan - subcategorization for Pretesticular/Endocrine genes. Green background - Strong evidence. Yellow background - Moderate evidence. Organge background - Weak evidence.

| Gene symbol | Locus | OMIM | Inheritance | Phenotype MIM | GU (or endocrine)  Phenotype(s) | Other associated  phenotypes | Reference(s) |
| --- | --- | --- | --- | --- | --- | --- | --- |
| NR5A1 | 9q33.3 | 184757 | AD | 617480 | 46, XX sex  reversal 4 | adrenocortical insufficiency | 29265478;28459839;27135758;27899089;27463801;27169744;26681172;25989977;24750329;24067197;23380608;23299922;21853106;20887963;29393271;29027717;29190620;29090230;  28326187;27711951;27610946;27553487;26260161;26492835;25502990;25160005;24591553;23969951;23729601;22909003;23096908;22907560;22474171;21535007;21163476;21340153;  17940071;17488792;17200175;15472171;27378692;28938747;30350900;30103258;30067310;29935645;29332064;29027299;32008008;30372699;29668062;29582157;32271476;32242295;  27490115 |
| NR5A1 | 9q33.3 | 184757 | AD | 612965 | 46 XY sex  reversal 3 | adrenocortical insufficiency | 29265478;28459839;27135758;27899089;27463801;27169744;26681172;25989977;24750329;24067197;23380608;23299922;21853106;20887963;29393271;29027717;29190620;29090230;  28326187;27711951;27610946;27553487;26260161;26492835;25502990;25160005;24591553;23969951;23729601;22909003;23096908;22907560;22474171;21535007;21163476;21340153;  17940071;17488792;17200175;15472171;27378692;28938747;30350900;30103258;30067310;29935645;29332064;29027299;32008008;30372699;29668062;29582157;32271476;32242295;  27490115 |
| NRAS | 1p13.2 | 164790 | AD | 614470  114500  162900  249400  613224  163200  188470 | Noonan syndrome 6 | ?RAS-associated autoimmune lymphoproliferativ e syndrome type IV, somatic?  Colorectal cancer, somatic Epidermal nevus, somatic Melanocytic nevus syndrome, congenital, somatic Neurocutaneous melanosis, somatic  Noonan syndrome 6 Schimmelpenning  -Feuerstein-Mims syndrome, somatic mosaic Thyroid carcinoma,  follicular, somatic | 29752777, 19966803, 26467218 |
| POR | 7q11.23 | 124015 | AR AR | 201750  613571  NA - PS 273250 | Antley-Bixler syndrome with genital anomalies and disordered steroidogenesis (horshoe kidney, micropenis, hypospadias, chordee, hypoplastic scrotum, bifid scrodum, cryptorchidism) Disordered steroidogenesis due to cytochrome P450 oxidoreductase Testicular regression syndrome per Houston et. al.  2020 |  | 34009138;30496128;33123976;32994263 |
| PPP2R3C | 14q13.2 | 615902 | AR | 618419 | Myoectodermal gonadal dysgenesis syndrome (complete gonadal dysgenesis, complete 46,XY female, intrafamilial variability seen - range of ambigous genitalia, unilateral renal agenesis has been seen) | Myoectodermal gonadal dysgenesis syndrome | 30893644;34714774;34750818;35812758;37147882 |

Supplementary table 1: Curated list of candidate genes with their associated phenotypic and indexing information. References listed as PMIDs. OMIM - Online Mendelian Inheritance in Man. GU - Genitourinary. AR - Autosomal recessive. AD - Autosomal dominant. SMu - Spontaneous mutation. XL - X-linked. XLR - X-linked recessive. XLD - X-linked dominant. NOA - Non-obstructive azoospermia. OA - Obstructive azoospermia. Green - strong evidence. Yellow - moderate evidence. Orange - weak evidence. Cyan - subcategorization for Pretesticular/Endocrine genes. Green background - Strong evidence. Yellow background - Moderate evidence. Organge background - Weak evidence.

| Gene symbol | Locus | OMIM | Inheritance | Phenotype MIM | GU (or endocrine)  Phenotype(s) | Other associated  phenotypes | Reference(s) |
| --- | --- | --- | --- | --- | --- | --- | --- |
| PRKAR1A | 17q24.2 | 188830 | AD  AD AD AD | 101800  160980  255960  610489 | Acrodysostosis 1, with or without hormone resistance (cryptorchidism) | Adrenocortical tumor, somatic Carney complex, type 1  Myxoma, intracardiac Pigmented nodular adrenocrotical disease, primary,  1 | 22464250, 22464252, |
| PTPN11 | 12p24.13 | 176876 | AD  AD AD AD | 151100  607785  156250  163950 | LEOPARD  syndrome 1 (cryptorchidism)  Noonan syndrome 1 (cryptorchidism, hypogonadism) | Leukemia, juvenile myelomonocytic, somatic Metachondromato sis | 11704759, 11992261, 12161469, 15723289, 12058348, 20308328, 20535210, 12325025, |
| RAD21L1 | 20p13 | n.a. | AR |  | n.r. RAD21L1 -/-  zebrafish were predominantly male due to late female to male  sex reversal | n.r. | 32741963;28635411;34138874 |
| RAF1 | 3p25.2 | 164760 | AD AD AD | 615916  611554  611553 | Noonan syndrome 5 (cryptorchidism in some patients) | Cardiomyopathy, dilated, 1NN Leopard syndrome 2 | 29752777;30904638 |
| RSPO1 | 1p34.3 | 609595 | AR AR | 610644  610644 | Palmoplantar hyperkeratosis and true hermaphroditism Palmoplantar hyperkeratosis with squamous cell carcinoma of skin and sex reversal |  | 26260363;25529318;24140641;16227098;29262419;17041600;18085567 |
| RXFP2 | 13q13.1 | 606655 | Undetermin ed | NA - PS 219050 | Variant of unknown significance - cryptorchidism |  | 21853106;19017913;15353080;12970298;12217959;19416190;17028442;15579790;15533513;14656401;20636340;31167797;37208861;34009138 |
| SIN3A | 15q24.2 | 607776 | AD | 613406 | Witteveen-Kolk syndrome (hypospadias, microphallus, cryptorchidism) |  | 25395209;22820070;34009138;36758531 |
| SOS1 | 2p22.1 | 182530 | AD AD | 135300  610733 | Noonan syndrome 4 | Fibromatosis, gingival, 1 | 29752777 |
| SOX3 | Xq27.1 | 313430 | XLD | 300833 | 46,XX sex reversal 3 |  | 26260363;15292361;25781358;25064402;22678921;21183788;27260338;29582157;36416214;36189645;36064700;35164824;34178900;31523625;35295983;35133534;30216942 |
| SOX9 | 17q24.3 | 608160 | AD | 114290  114290  114290 | Acampomelic campomelic dysplasia Campomelic dysplasia Campomelic dysplasia with autosomal sex reversal  46,XX disorders of sex development (Prader scale 4;5 or 6); per Houston  et. al 2020 |  | 37551848; 36064700; 34050715; 10588843; 27711951; 22570960; 30496128; 21208124; 26740947; 21653197; 21653197; 15266301; 24140641; 31700544; 25604083; 22051515; 21340153;  16556678; 26260363; 10588843; 18056774 |

Supplementary table 1: Curated list of candidate genes with their associated phenotypic and indexing information. References listed as PMIDs. OMIM - Online Mendelian Inheritance in Man. GU - Genitourinary. AR - Autosomal recessive. AD - Autosomal dominant. SMu - Spontaneous mutation. XL - X-linked. XLR - X-linked recessive. XLD - X-linked dominant. NOA - Non-obstructive azoospermia. OA - Obstructive azoospermia. Green - strong evidence. Yellow - moderate evidence. Orange - weak evidence. Cyan - subcategorization for Pretesticular/Endocrine genes. Green background - Strong evidence. Yellow background - Moderate evidence. Organge background - Weak evidence.

| Gene symbol | Locus | OMIM | Inheritance | Phenotype MIM | GU (or endocrine)  Phenotype(s) | Other associated  phenotypes | Reference(s) |
| --- | --- | --- | --- | --- | --- | --- | --- |
| SOX9 | 17q24.3 | 608160 | AD | 114290  114290  114290 | Acampomelic campomelic dysplasia Campomelic dysplasia Campomelic dysplasia with autosomal sex reversal  46,XY disorders of sex development (Prader scale 4;5 or 6); per Houston  et. al. 2020 |  | 37551848; 36064700; 34050715; 10588843; 27711951; 22570960; 30496128; 21208124; 26740947; 21653197; 21653197; 15266301; 24140641; 31700544; 25604083; 22051515; 21340153;  16556678; 26260363; 10588843; 18056774 |
| SRD5A2 | 2p23.1 | 607306 | AR | 264600 | Pseudovaginal perineoscrotal hypospadias 46,XY Disorders of Sex Development (Prader scale 4; 5  or 6); per Houston et. al.  2020 |  | 26681172;21334614;20305676;12137870;12009375;10368476;9077378;28663096;27849622;26780871;27086719;27051040;26446026;26492835;26453174;25899528;25605705;25077171;  24737579;24665940;24012728;23729601;23664981;21540559;21535007;21147889;20736251;20583543;20190539;20132346;20019388;19492581;18097518;16181229;15813602;15528927;  15266301;10999800;10564874;10458450;10092153;9843052;9745434;8789759;28938747;22453073;30269266;31613402;31219235;31186340;31031332;30815925;29582157;1406794 |
| SRY | Yp11.2 | 480000 | YL, XLD | 400045 | 46XX sex reversal 1 | tall stature, gynecomastia, gonadoblastoma | 28379671;27882599;27899089;27648416;26690523;25374395;25169080;24496683;24379036;23998093;23380608;23378127;23290744;22844483;21853106;21705820;20849656;20227075;  20170343;19205451;18414071;18412126;17762975;16769064;16530711;15652914;15579656;14686579;12137585;12095512;11278224;10101575;9733030;9004179;7726236;7782053;8383144;  29090230;27898418;27711951;27297128;25102093;24668626;24149105;24140641;23110663;23157850;20841307;21340153;20451191;19752597;19531589;19143733;18972202;18701099;  18056774;17183475;16218050;16045561;15751609;15665984;15641705;15579790;15266301;14689056;12890966;12818524;12483463;11694229;9521592;9341880;9030973;8710915;8725494;  7586641;7966049;1471700;1487232;30055081;31700544;31423422;31336995;30372699;29668062;29582157;32268053;31860174;23624391 |
| SRY | Yp11.2 | 480000 | YL, XLD | 400044 | 46XY sex reversal 1 | tall stature, gynecomastia, gonadoblastoma | 28379671;27882599;27899089;27648416;26690523;25374395;25169080;24496683;24379036;23998093;23380608;23378127;23290744;22844483;21853106;21705820;20849656;20227075;  20170343;19205451;18414071;18412126;17762975;16769064;16530711;15652914;15579656;14686579;12137585;12095512;11278224;10101575;9733030;9004179;7726236;7782053;8383144;  29090230;27898418;27711951;27297128;25102093;24668626;24149105;24140641;23110663;23157850;20841307;21340153;20451191;19752597;19531589;19143733;18972202;18701099;  18056774;17183475;16218050;16045561;15751609;15665984;15641705;15579790;15266301;14689056;12890966;12818524;12483463;11694229;9521592;9341880;9030973;8710915;8725494;  7586641;7966049;1471700;1487232;30055081;31700544;31423422;31336995;30372699;29668062;29582157;32268053;31860174;23624391 |
| TP63 | 3q28 | 603273 | AD AD AD AD  AD AD AD | 103285  604292  106260  603543  618149  620311  129400  605289 | Ectrodactyly, ectodermal dysplasia, and cleft lip/palate syndrome 3 (cryptorchidism, urethral stenosis) Hay-Wells syndrome (hypospadias)  Premature ovarian failure 21 Rapp-hodgkin syndrome (hypospadias) | ADULT syndrome  Limb-mammary syndrome Orofacial cleft 8  Split-hand/foot malformation 4 | 12401986, 31792669, 21204238, 10535733, 12939657, 11159940, 12838557, 14684701 |
| WNT4 | 1p36.12 | 603490 | AR ADR | 611812  158330 | ?SERKAL  syndrome? Mullerian aplasia and hyperadrogenism |  | 21340153;14689056;31700544 |
| WT1 | 11p13 | 607102 | AD, SMu AD, SMu AD  AD  AD,Smu AD | 194080  136680  608978  156240  256370  194070  NA - PS400044 | 46,XY Disorders of Sex Development (Prader scale 4; 5  or 6) without Wilm's tumor - per Houston et. al.  2020 | Denys-Drash syndrome Frasier syndrome Meacham syndrome Mesothelioma Nephrotic syndrome, type 4  Wilms tumor, type 1 | 27990711;25451826;24912414;23935527;23380608;28878596;27898418;21508141;15665984;15266301;11241055;10092153;8411073;30496128;29668062;29582157;32493750;36796343;  36496321;36382929;35498778;35432193;34392242;32780953;34009138 |
| ZFPM2 | 8q23.1 | 603693 | AD AD | 616067  610187  187500 | 46,XY sex reversal 9 | Diaphragmatic hernia 3 Tetralogy of Fallot | 24549039;36017582 |

| Gene symbol | Locus | OMIM | Inheritance | Phenotype  MIM | GU Phenotype(s) | Other associated  phenotypes | Reference(s) |
| --- | --- | --- | --- | --- | --- | --- | --- |
| ABCA1 | 9q31.1 | 600046 | AD AR | 604091  205400 | Tangier disease | HDL deficiency, familial, 1 | 29198592 |
| ABCG8 | 2p21 | 605460 | AR  Undetermin ed | 611465  210250  NA - PS258150 | Non-obstructive azoospermia - per Houston et. al. 2020 | {Gallbladder disease 4}  Sitosterolemia 1 | 31377750 |
| ACE | 17q23.2 | 106180 | AR  Undetermin ed | 612624  614519  267430  NA - PS258150 | Renal tubular dysgenesis  Non-obstructive azoospermia - per Houston et. al. 2020 | [Angiotensin I-converting enzyme, benign serum increase]  {Microvascular complications of diabetes 3}  {Myocardial infarction, susceptibility to}  {SARS, progression of}  {Stroke, hemorrhagic} | 11870238;31377750;37992807 |
| ACR | 22q13.33 | 102480 | AR | 620500 | ?Spermatogenic failure 87? (total fertilization failure) |  | 12801583;37004249 |
| ACTL7A | 9q31.3 | 604303 | AR | 620499 | Spermatogenic failure 86 |  | 17644991;31310081;37991128;37667331;37286336;36734600;36593593;36574082;35921706;35863052;34727571;32923619 |
| ACTL7B | 9q41,3 | 604304 | AR | NA - PS258150 | Azoospermia and oligozoospermia per Houston et. al. 2020 |  | 17644991;31310081;37800308;36617158 |
| ACTL9 | 19p13.2 | 619251 | AR | 619528 | Spermatogenic failure 53 | n.r. | 33626338 |
| ADAD1 | 4q27 | 614130 | Undetermin ed |  | n.r.  severe teratospermia observed in ADAD1 variant mice  See sources for WES findings in  infertile men |  | 36708028; 32665638 |
| ADAD2 | 16q24.1 | 619532 | AR |  | n.r.  ADAD2 variant mice germ cells unable to progress beyond round spermatid  found to be associated with incomplete SGA in two patients belonging to two independent  cohorts | n.r. | 36708028; 32741963 |
| ADAM20 | 14q24.2 | 603712 | Undetermin ed |  | Fertilization failure - per Houston et. al. 2020 |  | 29416755 |
| ADCY10 | 1q24.2 | 605205 | AD AR | NA - PS258150 | {Hyoercalciuria, absorptive, susceptibility to} Asthenozoospermia - per Houston et. al. 2020 |  | 21255775;31119281;33574797 |
| AGFG1 | 2q26.2 | 600862 | AR | NA -  PS258150 | Globozoospermia per Houston et.  al. 2020 |  | 16400072 |
| AK7 | 14q32.2 | 615364 | AR | 617965 | ?Spermatogenic failure 27? (MMAF) | may contribute to primary ciliary dyskinesia | 29365104;34854019;34529793 |
| AKAP3 | 12p13.32 | 604689 | AR | 620353 | Spermatogenic failure 82 (MMAF) |  | 25877373;25652624;16009167;15980003;11229805;38145487;35228300;31969357 |
| AKAP4 | Xp11.22 | 300185 | XL | NA - PS 305700 | Spermatogenic failure (MMAF) | n.r. | 25877373;25652624;21255775;16009167;15980003;11476771;11229805;38050179;34415320;34409659 |
| ALG13 | Xq23 | 300776 | XL  Undetermin ed | 300884  NA - PS305700 | Spermatogenic failure - 1 carrier identified by WGS of men with NOA | Developmental and epileptic encephalopathy 36 | 36017582 |
| ALKBH5 | 17p11.2 | 613303 | Undetermin ed | NA - PS258150 | Male infertility per Houston et. al. 2020 |  | 26820768;36270636;35719406 |
| ALOX15 | 17p13.2 | 152392 | AR | NA -  PS258150 | NOA per Houston et. al. 2020 |  | 31377750 |
| AMELY | Yp11.2 | 410000 | YL | NA - PS400042 | Spermatogenic failure |  | 15726419;33574797 |
| APOA1 | 11q23.2 | 107680 | AD AR AD | 105200  618463  619836 | Amyloidosis, 3 or more types (unclear if intratesticular obstructive azoospermia or nonobstructive azoospermia due to parenchymal replacement with amyloid) | Hypoalphalipoproteinemia, primary, 2 Hypoalphalipoproteinemia, primary, 2 | 15131802; 17507040; 18285420; 24925720; 25565309; 29446975 |

| Gene symbol | Locus | OMIM | Inheritance | Phenotype  MIM | GU Phenotype(s) | Other associated  phenotypes | Reference(s) |
| --- | --- | --- | --- | --- | --- | --- | --- |
| APOE | 19q13.32 | 107741 | AD AD  AD  AR  Undetermin ed | 607822  603075  617347  104310  617347  611771  269600  NA - PS258150 | Lipoprotein glomerulopathy  Male infertility per Houston et. al. 2020 | {?Alzheimer disease, protein against, due to APOE3-Christchurch}?  {?Macular degeneration, age-related{?  {Coronary artery disease, severe, susceptibiltiy to} Alzheimer disease 2 Hyperlipoproteinemia, type III  Sea-blue histiocyte disease | 31377750 |
| AR | Xq12 | 313700 | XL | NA - PS 305700 | Non-Obstructive Azoospermia - per Houston et. al. 2020 |  | 29264534;28659371;27498682;26681172;25677139;25532578;25433660;23808476;22469007;21962961;21127309;20305676;  20150575;19937586;19361789;19017913;17054461;16400085;14756668;12843171;12801573;11788673;11545293;11422119;  10999818;10976396;10946887;10935543;10852459;10470409;10359561;10333364;9851768;9788719;9607727;9433918;7926115;  7993455;1424203;1598912;1750490;29396419;29267169;29051026;29393262;29278518;28261839;27849622;27403927;  27087292;27051040;26935236;26688387;26352087;26778393;26492835;26197461;25605705;25241384;24907477;24737579;  24577144;24367986;23729601;23589523;22334658;21535007;20056211;20333878;19851057;19601939;18697867;18656523;  17937062;16448442;24790336;16283146;16151980;15963062;15925895;15522944;15486055;15541764;15452386;15266301;  15171708;15053245;15001585;14646391;19125473;12713250;12705360;12068007;12050225;12006704;11818512;11807912;  11701709;11591424;11587068;11579211;11549642;11397856;11260228;11238515;12058191;10999816;10971094;10690872;  10590024;10571951;10543676;10502786;10458483;10092153;10022458;9856504;9768671;9720578;9627582;9543136;9519369;  9360511;9345099;9302173;9245853;9106550;9160185;9156036;9010714;9039340;9001799;8823308;8768864;8683794;8809734;  8647313;8824883;7673412;7581399;7929841;7970939;8033918;8128958;8126121;8246999;8274427;8325932;8450042;8097257;  8446106;1307250;1303262;1316540;1356901;1307741;2001749;29785970;30496128;30411392;30251955;32155011;31377750;  31373714;31219235;30815925;30601762;30372699;29582157;32469048;32435981;32345305;32242295;32229106 |
| ARID5B | 10q21.2 | 608538 | AR | NA - PS258150 | Non-obstructive azoospermia per Houston et. al. 2020 |  | 31377750;35669193 |
| ARL2BP | 16q13 | 615407 | AR AR | 615434  NA - 258150 | Asthenozoospermia per Houston et. al. 2020 | Retinitis pigmentosa with or without situs inversus | 31425546;36507858 |
| ARL6 | 3q11.2 | 608845 | AR, DR AR  AR | 209900  600151  613575 | {Bardet-Biedl syndrome 1, modifier of}  Bardet-Biedl syndrome 3 | Retinitis pigmentosa 55 | 8298649; 15258860; 15314642; 23219996; 24608809; 24849935; 26518167; 28502102; 31888296; 33046855; 32361989 |
| ARMC2 | 6q21 | 618424 | AR | 618433 | Spermatogenic failure 38 (MMAF) | n.r. | 30686508; 34493464; 35543806 |
| ARMCX4 | Xq22.1 | 301046 | XL | NA - PS258150 | Non-obstructive azoospermia - per Houston et. al. 2020 |  | 32376790 |
| ART3 | 4q21.1 | 603086 | Undetermin  ed | NA -  PS258150 | Non-obstructive azoospermia -  per Houston et. al. 2020 |  | 28718531 |
| ATM | 11q22.3 | 607585 | AR  AD, SMu | 208900  114480 | Ataxia-telangiectasia (hypogonadism and impaired spermatogenesis reported) | Lymphoma, B-cell non-Hodgkin, somatic Lymphoma, mantle cell, somatic  T-cell prolymphocytic leukemia, somatic  {Breast cancer, susceptibility to} | 8689683; 8843194 |
| ATRX | Xq21.1 | 300032 | XLD, XLR | 300448  301040  309580 | Alpha-thalassemia myelodysplasia syndrome, somatic  Alpha-thalassemia/mental retardation syndrome (Shawl scrotum, small penis, hypospadias, cryptorchidism, renal agenesis, hydronephrosis) Intellectual disability-hypotonic facies syndrome, X-linked, 1 (hypogonadism, small testes, hypoplastic scrotum, micropenis, hypospadias, cryptorchidism, renal hypoplasia, vesicoureteral  reflux) | Alpha-thalassemia myelodysplasia syndrome, somatic  Alpha-thalassemia/mental retardation syndrome Intellectual disability-hypotonic facies syndrome,  X-linked, 1 | 35124761 |
| ATXN1 | 6p22.3 | 601556 | AD AR | 164400  NA - PS258150 | Non-obstructive azoospermia | Spinocerebellar ataxia 1 | 31377750 |
| AURKC | 19q13.43 | 603495 | AR | 243060 | Spermatogenic failure 5 |  | 17435757; 19147683; 21733974; 22888167; 23273756; 24484996; 25530203; 25755131; 26341096; 28187507; 28801929;  30594972; 31455599; 32399982 |
| B4GALT1 | 9p21.1 | 137060 | AR AR  Undetermin  ed | 620364  607091  NA | Fertilization failure - per Houston et. al. 2020 | Combined low LDL and fibrinogen  Congenital disorder of  glycosylation | 2567120 |

| Gene symbol | Locus | OMIM | Inheritance | Phenotype  MIM | GU Phenotype(s) | Other associated  phenotypes | Reference(s) |
| --- | --- | --- | --- | --- | --- | --- | --- |
| BBS1 | 11q13.2 | 209901 | AR, DR | 209900 | Bardet-Biedl syndrome 1 (Hypogonadism, renal anomalies, hypogentalism) | Bardet-Biedl syndrome 1 | 12118255; 12524598; 12567324; 12677556; 12837689; 15314642; 15770229; 20120035; 20472660; 28143435; 36833331 |
| BBS10 | 12q21.2 | 610148 | AR | 615987 | Bardet-Biedl syndrome 10  (hypogonadism, renal anomalies, cystic kidneys, renal failure) | Bardet-Biedl syndrome 10 | 16582908; 20805367; 16823392; 20120035; 20472660; 2104490; 23403234; 28143435; 37293956 |
| BBS12 | 4q27 | 610683 | AR | 615989 | Bardet-Biedl syndrome 12 (hypogonadism, renal anomalies (in some patients)) | Bardet-Biedl syndrome 12 | 17160889; 20120035; 31888296; 20472660; 21044901; 24849935; 37293956 |
| BBS2 | 16q13 | 606151 | AR AR | 615505  616562 | Bardet-Biedl syndrome 2 (hypogenitalism, hypogonadism, renal hypodysplasia (in some patients), kidney malrotation) Retinitis pigmentosa 74 | Bardet-Biedl syndrome 2  Retinitis pigmentosa 74 | 9039982; 11285252; 11567139; 11179009; 12677556; 12837689; 15770229; 16877420; 20120035; 20472660; 20618352;  21044901; 24608809; 24849935; 26078953; 26325687; 28143435; 28800606; 33520300; 36833331; 37031301 |
| BBS4 | 15q24.1 | 600374 | AR | 615982 | Bardet-Biedl syndrome 4 (cryptorchidism, hypogenitalism, hypogonadism renal cysts, renal  anomalies) | Bardet-Biedl syndrome 4 (can involved olfactory impairment) | 11381270; 12016587; 24849935; 37031301; 37293956 |
| BBS5 | 2q31.1 | 603650 | AR | 615983 | Bardet-Biedl syndrome 5 (hypognitalism, hypogonadism, renal abnormalities) | Bardet-Biedl syndrome 5 | 15137946; 18203199; 31888296; 16877420; 26325687 |
| BBS7 | 4q27 | 607590 | AR | 615984 | Bardet-Biedl syndrome 7  (hypogenitalism, renal abnormalities) | Bardet-Biedl syndrome 7 | 12567324; 16877420; 20472660; 21937992; 24849935; 36833331; 37293956 |
| BBS9 | 7p14.3 | 607968 | AR | 615986 | Bardet-Biedl syndrome 9 (renal disease) | Bardet-Biedl syndrome 9 | 16380913; 22353939; 31888296; 20120035; 20472660; 24849935 |
| BCL2L2 | 14q11.2 | 601931 | AR | NA -  PS258150 | Non-obstructive azoospermia per  Houston et. al. 2020 |  | 19369647 |
| BCORL1 | Xq26.1 | 300688 | XLR XL | 301029  NA - PS305700 | Non-obstructive azoospermia per Houston et. al. 2020 |  | 32376790; 38342987; |
| BEND2 | Xq22.13 | NA | Undetermin ed | NA - PS305700 | Spermatogenic failure - 1 carrier identified by WGS of men with NOA |  | 36017582 |
| BMPR1B | 4q22.3 | 603248 | AR AD AD AD | 609441  616849  112600  NA - PS258150 | Acromesomelic dysplasia 3 (hypergondotropic hypogonadism)  Azoospermia per Houston et. al. 2020 | Brachydactyly, type A1, D Brachydactyle, type A2 | 29581481 |
| BOLL | 2q33.1 | 606165 | AR | NA - PS258150 | Azoospermia and oligozoospermia per Houston et. al. 2020 |  | 15705409;15379971;37957469 |
| BPY2 | Yq11.223 | 400013 | YL | NA - PS400042 | Azoospermia and  oligozoospermia per Houston et. al. 2020 |  | 27203195;34462535;33781384 |
| BRCA1 | 17q21.31 | 113705 | AD, Mu  AR  Undetermin ed | 604370  614320  617883  NA - PS258150 | Male infertility per Houston et. al. 2020 |  | 31792669 |
| BRCA2 | 13q13.1 | 600185 | AD, SMu AD  AR  AD, AR,  SMu  AD, SMu, XL  AR  AD, SMu  Undetermin ed | 114480  612555  613029  155255  613347  176807  605724  194070  NA - PS258150 | {Prostate cancer} Fnaconi anemia,  complementation group D1 Wilms tumor  Non-obstructive azoospermia | {Breast cancer, male, susceptibility to}  {Breast-ovarian cancer, familial, 2}  {Glioblastoma 3}  {Medulloblastoma}  {Pancreatic cancer 2} | 31377750;34987987 |
| BRD2 | 6p21.32 | 601540 | Undetermin ed | NA - PS258150 | Non-obstructive azoospermia - per Houston et. al. 2020 |  | 31377750; 32860660 |
| BRDT | 1p22.1 | 602144 | AR | 617644 | ?Spermatogenic failure 21?  (acephalic sperm) | n.r. | 25739334; 28199965; 31377750; 32469048 |
| BRDT | 1p22.1 | 602144 | AR | 617644 | Non-obstructive azoospermia | n.r. | 25739334; 28199965; 31377750; 32469048 |

| Gene symbol | Locus | OMIM | Inheritance | Phenotype  MIM | GU Phenotype(s) | Other associated  phenotypes | Reference(s) |
| --- | --- | --- | --- | --- | --- | --- | --- |
| BRWD1 | 21q22.2 | 617824 | AR | 620438 | Ciliary dyskinesia, primary, 51 (infertility, reduced sperm count (in some patients), MMAF) |  | 25739334;33389130 |
| BRWD1 | 21q22.2 | 617824 | AR | 620438 | Non-obstructive azoospermia per  Houston et. al. 2020 |  | 25739334;33389130 |
| BRWD3 | Xq21.1 | 300533 | XLR  Undetermin ed | 300659  NA - PS305700 | Intellectual developmental disorder, X-linked 93 (cryptorchidism in some patients) Spermatogenic failure - 2 carries identified by WGS of men with NOA | Intellectual developmental disorder, X-linked 93 | 36017582 |
| BSCL2 | 11q12.3 | 606158 | AR AR AD AD | 615924  269700  619112  270586  NA - PS | Teratozoospermia | Encephalopathy, progressive, with or without lipodystrophy Lipodystrophy, congenital generalized, type 2 Neuronopathy, distal hereditary motor, autosomal dominant 13  Silver spastic paraplegia  syndrome | 24778225 |
| C14orf39 | 14q23.1 | 617307 | AR | ?619203?  619202 | ?premature ovarian failure 18? Spermatogenic failure 52 | n.r. | 33508233; 35172124; 27796301 |
| C1ORF146 | 1p22.1 | 618968 | Undetermin ed | NA - PS258150 | NOA - identified by WES + 2  stage burden testing vs fertile controls |  | 36572685 |
| C2CD6 | 2q33.1 | 619776 | AR | 619805 | ?Spermatogenic failure 68? |  | 31985809;34919125 |
| CACNA2D1 | 7q21.11 | 114204 | AR AD | 620149  NA - PS258150 | Azoospermia per Houston et. al. 2020 | Developmental and epileptic encephalopathy 110 | 24937803 |
| CAMK4 | 5q22.1 | 114080 | Undetermin ed | NA - PS258150 | Azoospermia per Houston et. al. 2020 |  | 22897820 |
| CAPZA3 | 12p12.3 | 608722 | AR | NA | Male infertility per Houston et. al.  2020 |  | 17644991 |
| CATIP | 2q35 | 619387 | AR | 619379 | ?Spermatogenic failure 54? |  | 32503832 |
| CATSPER1 | 11q13.1 | 606389 | AR | 612997 | Spermatogenic failure 7 | n.r. | 19344877; 21255775 |
| CATSPER2 | 15q15.3 | 607249 | ?AR? | 611102 | Deafness - infertility syndrome (asthenoteratozoospermia) | Hearing loss, sensorineural (bilateral, prelingual) | 12825070; 17098888; 21255775; 24690399; 30629171 |
| CATSPER2 | 15q15.3 | 607249 |  | n.r. | Asthenozoospermia without deafness |  | 12825070; 17098888; 21255775; 24690399; 30629171 |
| CATSPER2 | 15q15.3 | 607249 |  | n.r. | Normozoospermia with  fertilization failure with CATSPER2 disruption |  | 12825070; 17098888; 21255775; 24690399; 30629171 |
| CATSPER2 | 15q15.3 | 607249 |  | n.r. | Oligozoospermia |  | 12825070; 17098888; 21255775; 24690399; 30629171 |
| CATSPER3 | 5q31.1 | 609120 |  | n.r. | Asthenozoospermioa |  | 21255775; 17344468; 33350607 |
| CATSPER4 | 1p36.11 | 609121 |  | n.r. | Asthenozoospermioa |  | 21255775; 17344468 |
| CATSPERE | 1q44 | 617510 |  | n.r. | Variant of unknown significance - spermatogenic failure |  | 30239785; 26453676 |
| CCDC103 | 17q21.31 | 614677 | AR | 614679 | Ciliary dyskinesia, primary, 17 |  | 25877373; 36777727;35259782;33988008 |
| CCDC103 | 17q21.31 | 614677 | AR | NA - PS258150 | Nonsyndromic asthenozoospermia |  | 25877373; 36777727;35259782;33988008 |
| CCDC33 | 15q24.1 | 618525 | Undetermin  ed | NA -  PS258150 | NOA - identified by WES from  GEMINI cohort |  | 36572685;36259570 |
| CCDC39 | 3q26.6 | 613798 | AR | 613807 | Ciliary dyskinesia, primary, 14 Oligoasthenozoospermia (MMAF) according to Aprea et. al 2023 | ciliary dyskinesia, primary, 14 | 21131972; 22693285; 25877373; 31469207; 34674941; 33988008; 35795318; 36873931 |
| CCDC39 | 3q26.6 | 613798 | AR | NA - PS  258150 | asthenozoospermia per houston  review | ciliary dyskinesia, primary, 14 | 21131972; 22693285; 25877373; 31469207; 34674941; 33988008; 35795318; 36873931 |
| CCDC40 | 17q25.3 | 613799 | AR | 613808 | Ciliary dyskinesia, primary, 15 Oligoasthenozoospermia (MMAF) according to Aprea et. al 2023 | ciliary dyskinesia, primary, 14 | 21131974; 22693285; 25619595; 25877373; 29456554; 33574797; 34941110; 35449766; 36873931 |
| CCDC40 | 17q25.3 | 613799 | AR | NA - PS  258150 | Asthenozoospermia | ciliary dyskinesia, primary, 14 | 21131974; 22693285; 25619595; 25877373; 29456554; 33574797; 34941110; 35449766; 36873931 |
| CCDC62 | 12q24.31 | 613481 | AR | 619803 | ?Spermatogenic failure 67? |  | 31985809;28339613 |

| Gene symbol | Locus | OMIM | Inheritance | Phenotype  MIM | GU Phenotype(s) | Other associated  phenotypes | Reference(s) |
| --- | --- | --- | --- | --- | --- | --- | --- |
| CCDC89 | 11q14.1 | N/A | Undetermin ed | NA - PS 258150 | spernatogenic failure - identified through analyses of the GEMINI cohort with 2 stage burden testing |  | 36572685 |
| CCDC9 | 19q13,32 | N/A | AR | NA - PS  258150 | Asthenozoospermia - per  Houston et. al. 2020 |  | 31502483;38073178 |
| CCIN | 9p13.3 | 603960 | AR | NA - PS 258150 | Variant of unknown significance - spermatogenic failure (globozoospermia) |  | 31985809;36546111;36527329 |
| CCNA1 | 13q13.3 | 604036 | Undetermin ed | NA - PS258150 | Azoospermia and  oligozoospermia per Houston et. al. 2020 |  | 19886767 |
| CD46 | 1q32.2 | 120920 | AD, AR AR | 612922  NA | Idiopathic male infertility per Housotn et. al. 2020 | {Hemolytic uremic syndrome, atypical, susceptibility to, 2} | 11150852 |
| CDC14A | 1p21.2 | 603504 | AR | 608653 | Deafness, autosomal recessive 32, with or without immotile sperm  (Infertility (in some patients), immotile sperm (in some patients), abnormal sperm  morphology (in some patients)) |  | 29293958; 32679235 |
| CDC20 | 1p34.2 | 603618 | AR | 620276  NA - PS258150 | Oocyte/zygote/embryo maturation arrest 14 (female infertility) Association pending confirmation  - spermatogenic failure |  | 29245942 |
| CDC45 | 22q11.21 | 603465 | AR  Undetermin ed | 617063  NA - PS258150 | Spermatogenic failure - 1 carrier within GEMINI cohort | Meier-Gorlin sydrome 7 | 35719406;36572685 |
| CDK2 | 12p13.2 | 116953 | AD | NA - PS258150 | Non-obstructive azoospermia per Houston et. al. 2020 |  | 18941885;29373224 |
| CDKN2A | 9p21.3 | 600160 | AD AD AD  Undetermin  ed | 155755  606719  155601  NA - PS258150 | Male infertility per Houston et. al. 2020 | {Melanoma and neural system tumor syndrome}  {Melanoma-pancreatic cancer syndrome}  {Melanoma, cutaneous malignant, 2} | 31792669 |
| CDY1 | Yq11.23 | 400016 | YL | NA - PS400042 | Non-obstructive azoospermia per Houston et. al. 2020 |  | 27203195;35358715 |
| CELSR3 | 3p21.31 | 604264 | Undetermin  ed | NA -  PS258150 | NOA - identified by WES from  GEMINI cohort |  | 36572685 |
| CEP112 | 17q24.1 | 618980 | AR | 619044 | Spermatogenic failure 44 | n.r. | 31654588 |
| CEP135 | 4q12 | 611423 | AR AR | 614673  NA - PS617576 | Variant of unknown significance - spermatogenic failure | Microcephaly 8, primary, autosomal recessive | 28866084 |
| CEP164 | 11q23.3 | 614848 | AR | 614845 | Nephronopthisis 15 |  | 27708425 |
| CEP290 | 12q21.32 | 610142 | AR AR  AR AR | 615991  610188  611755  611134  610189 | ?Bardet-Biedl syndrome 14?  Joubert syndrome 5  Leber congenital amaurosis 10 Meckel syndrome, type 4 Senior-Loken syndrome 6 | ?Bardet-Biedl syndrome 14?  Joubert syndrome 5  Leber congenital amaurosis 10  Meckel syndrome, type 4  Senior-Loken syndrome 6 | 18327255; 24608809; 22355252 |
| CEP78 | 9q21.2 | 617110 | AR AR | 617236  NA - PS617236 | Oligoasthenoteratospermia in combination with cone-rod dystrophy and hearing loss | Cone-rod dystrophy and hearing loss 1 | 31999394;36756949;36206347 |
| CFAP251 | 12q24.31 | 618146 | AR | 618152 | Spermatogenic failure 33 | n.r. | 30122540; 30122541; 30310178; 30686508 |
| CFAP298 | 21q22.11 | 615494 | AR | 615500 | Ciliary dyskinesia, primary, 26 | cliary dyskinesia, primary, 26 (with and without situs inversus) | 24094744 |
| CFAP300 | 11q22.1 | 618058 | AR | 618058 | Ciliary dyskinesia, primary, 28 |  | 29727693;33635866 |
| CFAP43 | 10q25.1 | 617558 | AR | 236690 | Spermatogenic failure 19 | Hydrocephalus, normal-pressure, 1 | 29277146;28552195;29449551;30251428;30904354;30811583;30686508;32207550;34089056;34529793;34100391 |
| CFAP44 | 3q13.2 | 617559 | AR | 617593 | Spermatogenic failure 20 | n.r. | 29277146;28552195;29449551;30251428;30904354;30811583;30686508;34529793;34089056 |
| CFAP47 | Xp21.1 | 301057 | XLR | 301059 | Spermatogenic failure, X-linked 3 | n.r. | 33472045; 36571501; 37424856 |
| CFAP58 | 10q25.1 | 619129 | AR | 619144 | Spermatogenic failure 49 | n.r. | 32791035; 33314088; 34089056 |
| CFAP65 | 2q35 | 614279 | AR | 618664 | Spermatogenic failure 40 | n.r. | 28552195; 31413122; 31501240; 31571197; 34914225 |
| CFAP69 | 7q21.13 | 617949 | AR | 617959 | Spermatogenic failure 24 | n.r. | 29606301; 30415212; 34529793; 37392306 |
| CFAP70 | 10q22.2 | 618661 | AR | 618670 | ?Spermatogenic faiure 41? | n.r. | 31621862; 37352829 |

| Gene symbol | Locus | OMIM | Inheritance | Phenotype  MIM | GU Phenotype(s) | Other associated  phenotypes | Reference(s) |
| --- | --- | --- | --- | --- | --- | --- | --- |
| CFAP74 | 1p36.33 | 620187 | AR | 620197 | Ciliary dyskinesia, primary, 49, without situs inversus |  | 32555313 |
| CFAP91 | 3q13.33 | 609910 | AR | 619177 | Spermatogenic failure 51 | n.r. | 32161152 |
| CFTR | 7q31.2 | 602421 | AR | NA - PS 258150 | Non-obstructive azoospermia or oligozoospermia - per Houston Review | Cystic fibrosis  Sweat chloride elevation without CF Bronchiectasis with or without elevated sweat chloride 1  Hypertrypsinemia, neonatal  pancreatitis, hereditary | 28801929;28456595;28196530;27793385;26946416;27488005;27364092;26989879;26277102;25944622;25443471;25536748;  25386751;25308578;25304080;25010724;24958810;24551851;24451227;24149827;23953609;23687349;23555973;23378603;  25246892;22483971;22390181;22340520;22191729;22148899;22103471;21762191;21976147;21679131;21609195;21507732;  21254931;21219377;21067729;20609027;20691141;20657600;20381036;20021716;19810821;19737283;19298730;19181743;  18810634;18796364;18755906;18304229;18078365;17681314;17673436;17662673;17572159;17823699;17617039;17482604;  17448246;17398169;17394391;17329263;17254580;17127107;17050329;16973827;16840743;16714368;16581722;16572913;  16481891;16412743;16272798;16128988;15905293;15870824;15705389;15644056;15533383;15482777;15357566;15333598;  15239534;15070876;14998948;14747162;15463840;12801574;12741342;12475673;12151438;12009340;11993238;11788091;  11574497;11298840;11119745;11101688;10923036;10878476;10875853;10871656;10655317;10655318;10653141;28176072;  10601093;10376575;10341008;10099982;10066035;10050655;10200050;9797105;9736775;9678705;9630075;9620832;9620832;  9598638;9591500;9345100;9334604;9272157;9196095;9067761;9043501;8627844;8671256;8557264;8829643;8582970;7540706;  7539210;7539342;7739684;7539448;7532150;7529962;8556303;7738909;7968122;7513294;7516233;8301660;7505692;7692051;  8100886;8473422;1545465;17975025;17413420;10653048;8834261;7573058;7542209;7691870;7686336;8421472;1975022;  29864494;30450785;29986553;32020786;31823853;31709488;31377750;31357024;30811104;30389601;21875427;10651488 |
| CHD5 | 1p36.31 | 610771 | AD | 619873  NA - PS258150 | Non-obstructive azoospermia | Parenti-Mignot neurodevelopmental syndrome | 31792669;32469048 |
| CLCA4 | 1p22.3 | 616857 | Undetermin  ed | NA -  PS258150 | Azoospermia or oligozoospermia  per Houston et. al. 2020 |  | 23579007; 30887952; 32860660 |
| CLOCK | 4q12 | 601851 | Undetermin ed | NA - PS258150 | Oligozoospermia per Houston et. al. 2020 |  | 31377750 |
| CLPP | 19p13.3 | 601119 | AR | 614219  NA - PS258150 | NOA | Perrault syndrome 3 | 36041235;23851121 |
| COL1A1 | 17q21.33 | 120150 | AD AD AD AD AD AD AD AD  Undetermin  ed | 166710  114000  619115  130060  166200  166210  259420  166220  NA - PS258150 | Sertoli cell only syndrome per Houston et. al. 2020 | {Bone mineral density variation QTL, osteoporosis} Caffey disease  Combined osteogenesis imperfecta and Ehlers-Danlos syndrome 1 Ehlers-Danlos syndrome, arthrochalasia type, 1 Osteogenesis imperfecta, type I  Osteogenesis imperfecta, type II  Osteogenesis imperfecta, type III  Osteogenesis imperfecta, type IV | 30478911 |
| CREM | 10p11.21 | 123812 | AD | NA - PS258150 | Azoospermia - per Houston et. al. 2020 |  | 16143638;11044457 |
| CRISP2 | 6p12.3 | 187430 | AR | NA - PS 258150 | Asthenozoospermia and  teratozoospermia per Houston et. al. 2020 |  | 18550510;33037689 |
| CS | 12q13.3 | 118950 | AD | NA - PS 258150 | Azoospermia and oligozoospermia per Houston et. al. 2020 |  | 17298551 |
| CSMD1 | 8q23.2 | 608397 | Undetermin ed | NA - PS258150 | NOA - identified by WES + 2  stage burden testing vs fertile controls |  | 36572685;31604923 |
| CSNK2A2 | 16q21 | 115442 | Undetermin ed | NA - PS258150 | Globozoospermia per Houston et. al. 2020 |  | 16400072;15746197 |
| CSNK2B | 6p21.33 | 115441 | AD  Undetermin ed | 618732  NA - PS258150 | Globozoospermia per Houston et. al. 2020 | Poirier-Bienvenu  neurodevelopmental syndrome | 15746197 |
| CTCFL | 20q13.31 | 607022 | AR | NA - PS258150 | Oligoasthenoteratozoospermia per Houston et. al. 2020 |  | 23955684;35413094 |
| CUL4B | Xq24 | 300304 | XLR XL | 300354  NA - PS305700 | Intellectual developmental disorder, X-linked syndromic, Cabezas type  Sertoli cell only syndrome per  Houston et. al. 2020 |  | 28816568;26832838;26846852 |
| CYP1A1 | 15q24.1 | 108330 | AR | NA - PS258150 | Non-obstructive azoospermia per Houston et. al. 2020 |  | 31377750 |

| Gene symbol | Locus | OMIM | Inheritance | Phenotype  MIM | GU Phenotype(s) | Other associated  phenotypes | Reference(s) |
| --- | --- | --- | --- | --- | --- | --- | --- |
| CYP1B1 | 2p22.2 | 601771 | AR AR AR | 617315  231300  NA - PS258150 | Non-obstructive azoospermia - per Houston et. al. 2020 | Anterior segment dysgenesis 6, multiple subtypes Glaucoma 3A, primary open angle, congenital, juvenile, or adult onset | 31377750 |
| CYP26B1 | 2p13.2 | 605207 | Undetermin ed | 614416  NA - PS258150 | Non-obstructive azoospermia - per Houston et. al. 2020 | Craniosynostosis with radiohumeral fusions and other skeletal and craniofacial anomalies | 31377750 |
| DAZ1 | Yq11.223 | 400003 | YL | NA - PS258150 | Azoospermia per Houston et. al. 2020 |  | 9239708;36434389 |
| DAZL | 3p24.3 | 601486 | AR | NA NA -  PS258150 | {Spermatogenic failure, susceptibility to}  Azoospermia and  oligozoospermia per Houston et. al. 2020 |  | 27203195;16884537;16573709;16328470;12414900;11499325 |
| DCAF12L1 | Xq25 |  |  | NA - PS258149 | NOA - identified by WES + 2 stage burden testing vs fertile controls |  | 36572685; 35850260 |
| DDX25 | 11q24.2 | 607663 | AR | NA - PS  258150 | Spermatogenic failure |  | 35172124;24168058;18487219;17848414;16293649 |
| DDX3Y | Yq11.221 | 400010 | Y linked | NA - PS 400042 | Azoospermia - per Houston Review |  | 27203195; 28801929; 36997603 |
| DDX53 | Xq22.11 | 301079 | Undetermin ed | NA - PS305700 | Spermatogenic failure - 1 carrier  identified by WGS of men with NOA |  | 14531651;36017582 |
| DEFB126 | 20p13 | 620131 | AR | NA | Male infertility per Houston et. al. 2020 |  | 25721098;21775668; 31504198 |
| DHRSX | Xp22.33 | 301034 | Undetermin ed | NA - PS305700 | Spermatogenic failure - 1 carrier  identified by WGS of men with NOA |  | 36017582 |
| DMC1 | 22q13.1 | 602721 | AR |  | VUS for NOA  one report of only primary spermatocytes in seminiferous tubules and complete azoospermia in one homozygous variant patient | n.r. | 29331980; 34515795; 35172124 |
| DMRT1 | 9p24.3 | 602424 | AD |  | n.r.  NOA - Houston et. al. 2020  Adult male Dmrt1 -/- mice showed severe testis hypoplasia.  Microscopic and immunohistochemical examination revealed that Dmrt1 -  /- seminiferous tubules were disorganized, Sertoli cells failed to differentiate, and germ cells were missing, apparently due to premeiotic germ cell death. No ectopic ovarian tissue or Mullerian duct-derived structures were apparent in Dmrt1 -/- males. In contrast, Dmrt1 +/- males had normal testis and were fertile, and Dmrt1 -/- females had normal  ovaries and were fertile. |  | 32741963; 23555275; 36572623; 37070575 |
| DNAAF1 | 16q24.1 | 613190 | AR | 613193 | ciliary dyskinesia, primary, 13 | ciliary dyskinesia, primary, 13 situs inversus, dextrocardia, respiratory infections, sinusitis, bronchitis, bronchiectasis, | 19944400; 19944405; 33174003 |
| DNAAF2 | 14q21.3 | 612517 | AR | 612518 | Ciliary dyskinesia, primary, 10 (immotile sperm, absent inner and outer dynein arms of sperm flagella seen on transmission  electron microscopy) |  | 19052621; 32638265; 33635866 |

| Gene symbol | Locus | OMIM | Inheritance | Phenotype  MIM | GU Phenotype(s) | Other associated  phenotypes | Reference(s) |
| --- | --- | --- | --- | --- | --- | --- | --- |
| DNAAF3 | 19q13.42 | 614566 | AR | 606763 | Ciliary dyskinesia, primary, 2 | ciliary dyskinesia, primary, 2 otitis media, hearing loss, dextrocardia, respiratory distress, recurrent respiratory infections,sinusitis, nasal polyps, bronchiectasis, situs inversus (~50%) | 10745040; 22387996; 26139845; 35869935; 37537752 |
| DNAAF4 | 15q21.3 | 608706 | AR, AD | 615482  127700 | ciliary dyskinesia, primary, 25  dyslexia, susecptibility to, 1 | ciliary dyskinesia, primary, 25 | 23872636; 28801648; 37147940 |
| DNAAF5 | 7p22.3 | 614864 | AR | 614874 | ciliary dyskinesia, primary, 18 | ciliary dyskinesia, primary, 18 | 23040496; 25232951; 29363216 |
| DNAH1 | 3p21.1 | 603332 | AR | 617576 | Spermatogenic failure 18 | ?Ciliary dyskenesia, primary, 37? | 24360805; 27094479; 27573432; 27798045; 28577616; 29449551; 30811583; 30904354; 33989052; 37302001 |
| DNAH1 | 3p21.1 | 603332 | AR | 617577 | ?Ciliary dyskenesia, primary, 37?  (based on report of 2 sisters) | ?Ciliary dyskenesia, primary,  37? | 24360805; 27094479; 27573432; 27798045; 28577616; 29449551; 30811583; 30904354; 33989052; 37302001 |
| DNAH1 | 3p21.1 | 603332 | AR | 617577 | Azoospermia | ?Ciliary dyskenesia, primary, 37? | 24360805; 27094479; 27573432; 27798045; 28577616; 29449551; 30811583; 30904354; 33989052; 37302001 |
| DNAH1 | 3p21.1 | 603332 | AR | 617577 | Asthenoteratozoospermia | ?Ciliary dyskenesia, primary,  37? | 24360805; 27094479; 27573432; 27798045; 28577616; 29449551; 30811583; 30904354; 33989052; 37302001 |
| DNAH1 | 3p21.1 | 603332 | AR | 617577 | Oligoasthenoteratozoospermia | ?Ciliary dyskenesia, primary, 37? | 24360805; 27094479; 27573432; 27798045; 28577616; 29449551; 30811583; 30904354; 33989052; 37302001 |
| DNAH10 | 12q24.31 | 605884 | AR | 619515 | Spermatogenic failure 56 | n.r. | 34237282; 34657236; 34791246; 37314648 |
| DNAH11 | 7p15.3 | 603339 | AR | 611884 | Ciliary dyskinesia, primary, 7, with or without situs inversus (male infertility remains intact per OMIM) |  | 18492703; 31160482; 31377750; 25802884; 33574797; 33988008 |
| DNAH12 | 3p14.3 | 603340 | Undetermin ed | NA - PS617576 | Multiple morphological  abnormalities of the sperm flagella |  | 34791246;34089056 |
| DNAH17 | 17q25.3 | 610063 | AR | 618643 | Spermatogenic failure 39 | n.r. | 31178125; 31658987; 31841227; 31985809; 33070343; 37574497; 36048845; 35932098; 34126833; 33423959; 33108537 |
| DNAH2 | 17p13.1 | 603333 | AR | 603333 | Spermatogenic failure 45 | n.r. | 30811583; 34791246; 33968937; 33771466 |
| DNAH5 | 5p15.2 | 603335 | AR | 608644 | Ciliary dyskinesia, primary, 3, with or without situs inversus | Ciliary dyskinesia, primary, 3, with or without situs inversus | 11788826; 16627867; 18492703; 23261302; 25877373; 31377750 |
| DNAH6 | 2p11.2 | 603336 |  | NA - PS244400 | Association pending confirmation  - heteroteaxy and primary ciliary dyskinesia | n.r. | 37594300; 37424858; 34089056; 31676830; 29356036; 28206990; 26918822 |
| DNAH6 | 2p11.2 | 603336 |  | NA - PS258150 | Association pending confirmation  - spermatogenic failure 1 | n.r. | 37594300; 37424858; 34089056; 31676830; 29356036; 28206990; 26918822 |
| DNAH6 | 2p11.2 | 603336 | AR | NA -  PS258150 | Azoospermia - per Houston  Review | n.r. | 37594300; 37424858; 34089056; 31676830; 29356036; 28206990; 26918822 |
| DNAH6 | 2p11.2 | 603336 | AR | NA - PS258150 | Teratozoospermia; Globozoospermia and acephaly per Houston Review | n.r. | 37594300; 37424858; 34089056; 31676830; 29356036; 28206990; 26918822 |
| DNAH6 | 2p11.2 | 603336 | AR | NA - PS258150 | Multiple morphological  abnormalities of the sperm flagella per Houston review | n.r. | 37594300; 37424858; 34089056; 31676830; 29356036; 28206990; 26918822 |
| DNAH8 | 6q21.2 | 603337 | AR | 619095 | Spermatogenic failure 46 | n.r. | 24307375; 32619401; 32681648; 33611675; 34529793; 36308074; 35654582; 35672654; 36017582 |
| DNAH9 | 17p12 | 603330 | AR | 618300 | Ciliary dyskinesia, primary, 40 | n.r. | 33610189;30471717;35729109 |
| DNAI1 | 9p13.3 | 604366 | AR | 244400 | Ciliary dyskinesia, primary, 1, with or without situs inversus | ciliary dyskinesia, primary, 1, with or without situs inversus | 11231901; 11713099; 18492703; 25877373; 33574797 |
| DNAI1 | 9p13.3 | 604366 | AR | 244400 | asthenozoospermia per houston  review | ciliary dyskinesia, primary, 1,  with or without situs inversus | 11231901; 11713099; 18492703; 25877373; 33574797 |
| DNAI2 | 17q25.1 | 605483 | AR | 612444 | Ciliary dyskinesia, primary, 9, with or without situs inversus | Ciliary dyskinesia, primary, 9, with or without situs inversus | 18950741; 23261302; 33167880 |
| DNAL1 | 14q24.3 | 610062 | AR | 614017 | Ciliary dyskinesia, primary, 16  (infertility not reported) | Ciliary dyskinesia, primary,  16 | 21496787 |
| DNALI1 | 1p34.3 | 602135 | AR | 620354 | ?Spermatogenic failure 83? |  | 36792588;36726469 |
| DNAJB13 | 11p13.4 | 610263 | AR | 617091 | Primary ciliary dyskinesia |  | 27486783;31342671;35166991 |
| DND1 | 5q31.3 | 609385 | unclear | n.r. | non-obstructive azoospermia |  | 36807972; 36713075; 36246621 |
| DNMT1 | 18p13.2 | 126375 | AD AD AD | 604121  614116  NA - PS258150 | Non-obstructive azoospermia | Cerebellar ataxia, deafness, and narcolepsy, autosomal dominant  Neuropathy, hereditary sensory, type IE | 25739334 |

| Gene symbol | Locus | OMIM | Inheritance | Phenotype  MIM | GU Phenotype(s) | Other associated  phenotypes | Reference(s) |
| --- | --- | --- | --- | --- | --- | --- | --- |
| DNMT3A | 2p23.2 | 602769 | AD AD  Undetermin  ed | 601626  618724  615879  NA - PS258150 | Oligozoospermia | Acute myeloid leukemia, somatic  Heyn-Sproul-Jackson syndrome  Tatton-Brown-Rahman syndrome | 23916795 |
| DNMT3B | 20p11.21 | 602900 | DD AR AD | 619478  242860  NA - PS258150 | Non-obstructive azoospermia | Facioscapulohumeral muscular dystrophy 4, digenic Immunodeficiency-  centromeric instability-facial anomalies syndrome 1 | 25739334;31479588 |
| DNMT3L | 21q22.3 | 606588 | AR | NA - PS258150 | Azoospermia and oligozoospermia |  | 23916795;21126912 |
| DPP6 | 7q36.2 | 126141 | AD AD AD | 612956  616311  NA - PS258150 | Azoospermia | {Ventricular fibrillation, proxysmal familial, 2} Intellectual developmental disorder, autosomal dominant 33 | 24937803 |
| DPY19L2 | 12q14.2 | 613893 | AR | 613958 | Spermatogenic failure 9 (globozoospermia) | n.r. | 33108537; 21397063; 21397064; 21857011; 22627659; 22653751; 23512994; 24265589; 25755131; 26516168; 27441053;  28272055; 28801929; 29339016; 30333325; 30362053; 30584989; 30912172; 31985809; 32312381; 32582379 |
| DRC1 | 2p23.3 | 615228 | AR | 615294  620222 | Ciliary dyskinesia, primary, 21 Spermatogenic failure 80 | Ciliary dyskinesia, primary, 21 | 34815526; 34169321; 34089056; 31270959 |
| DYNLT3 | 6p25.3 | 300302 | XL | NA - 305700 | Azoospermia |  | 14531651 |
| DZIP1 | 13q32.1 | 608671 | AD, AR | 610840  619102 | Spermatogenic failure 47 (Asthenoteratospermia, absent or short flagella, centriolar abnormalities) | ?Mitral valve prolapse 3? | 32051257 |
| E2F1 | 20q11.22 | 189971 | AD | NA - PS258150 | Non-obstructive azoospermia |  | 25439843; 30659775 |
| EGR4 | 2p13.2 | 128992 | AR | NA -  PS258150 | Oligozoospermia and  azoospermia |  | 28464846 |
| EIF1AY | Yq11.223 | 400014 | YL | NA - PS400042 | Non-obstructive azoospermia |  | 31579209 |
| EIF2B2 | 14q24.3 | 606454 | Undetermin ed | 606454  NA - PS258150 | NOA | Leukoencephalopathy with vanishing white matter 2, with or without ovarian failure | 36041235 |
| EIF4G1 | 3q27.1 | 600495 | AD | 614251  NA - PS258150 | Non-obstructive azoospermia | {Parkinson disease 18} | 31268247 |
| EIF5A2 | 3q26.2 | 605782 | Undetermin ed | NA - PS258150 | Oligozoospermia and azoospermia |  | 16169419 |
| EPHX2 | 8p21.2-p21.1 | 132811 | AD, AR  Undetermin ed | 143890  NA - PS258150 | Non-obstructive azoospermia | {Hypercholesterolemia,  familial, due to LDLR defect, modifier of} | 31377750 |
| EPPIN | 20q13.12 | 609031 | Undetermin ed | NA | Male infertility |  | 27827323 |
| ESR2 | 14q23.2-23.3 | 601663 | AD  Undetermin ed | 618187  NA - PS258150 | ?Ovarian dysgenesis 8?  Oligozoospermia and azoospermia |  | 19509112 |
| ESX1 (ESX1L) | Xp22.2 | 300154 | Undetermin ed | NA - PS258150 | Spermatogenic failure - 2 carriers identified by WGS of men with NOA |  | 37783880; 36017582; 33633269 |
| ETV5 | 3q27.2 | 601600 | Undetermin  ed | NA -  PS258150 | Non-obstructive azoospermia |  | 22771031;24204802 |
| EXT1 | 8q24.11 | 608177 | SMu AD AD | 215300  133700  NA - PS258150 | Oligozoospermia | Chondrosarcoma Exostoses, multiple, type 1 | 14556251 |
| FABP9 | 8q21.13 | NA | AR | NA - PS258150 | Teratozoospermia |  | 24520497 |
| FAM9B | Xp22.31 | 300478 | XL | NA - PS305700 | Spermatogenic failure - 1 carrier  identified by WGS of men with NOA |  | 36017582;37594251 |
| FAM9C | Xp22.2 | 300479 | XL | NA - PS305700 | Spermatogenic failure - 1 carrier identified by WGS of men with NOA |  | 36017582 |

| Gene symbol | Locus | OMIM | Inheritance | Phenotype  MIM | GU Phenotype(s) | Other associated  phenotypes | Reference(s) |
| --- | --- | --- | --- | --- | --- | --- | --- |
| FAM47B | Xp21.1 | NA | XL | NA - PS305700 | Spermatogenic failure - 1 carrier identified by WGS of men with NOA |  | 36017582 |
| FAM47C | Xp21.1 | 301067 | XL | NA - PS305700 | Association pending confirmation  - spermatogenic failure (varicocele leading to oligozoospermia per Houston et. al 2020) |  | 30922974;36017582 |
| FAM50B | 6p25.2 | 614686 | Undetermin ed | NA - PS258150 | Spermatogenic failure - 1 carrier identified by WGS of men with NOA |  | 36017582 |
| FANCA | 16q24.3 | 607139 | AR | 227650 | Fancomi anemia (Cryptorchidism, Infertility (in some patients), NOA; Sertoli cell-only syndrome (in  some patients)) | Fanconi anemia | 29904161 |
| FANCB | Xp22.2 | 300515 | XLR | 300514 | Fanconi anemia, complementation group B (hypergonadotropic hypogonadism) | Fanconi anemia | 36017582 |
| FANCM | 14q21.2 | 609644 | AR | ?618096?  618086 | ?Premature ovarian failure 15?  Spermatogenic failure 28 | n.r. | 29895858; 30075111; 34976027; 36017582; 37601968 |
| FATE1 | Xq28 | 300450 | XL | NA - PS305700 | Oligoasthenozoospermia |  | 12811541 |
| FBXO15 | 18q22.3 | 609093 |  | NA - PS258150 | NOA - identified by WES + 2  stage burden testing vs fertile controls |  | 36572685 |
| FBXO43 | 8q22.2 | 609110 | AR | 619697  619696 | Oocyte/zygote/embryo maturation arrest 12 (female infertility) Spermatogenic failure 64 (oligoasthenoteratozoospermia) |  | 34595750; 30878252. |
| FHL5 | 6q16.1 | 605126 | AR | NA -  PS258150 | Azoospermia and  oligozoospermia |  | 16687568 |
| FKBP6 | 7q11.23 | 604839 | AR | 620103 | Spermatogenic failure 77 (cryptorchidism (unilateral), small testis (unilateral), azoospermia, oligozoospermia, sperm head abnormalities, late meiosis arrest, round spermatid arrest (early spermiogenesis)) |  | 36150389 |
| FKBPL | 6q21.32 | 617076 | AD | NA - PS 258150 | Azoospermia |  | 20110997; 34212559; 32860660 |
| FMR1NB | Xp27.3-q28 | NA | XL | NA - PS 258150 | Spermatogenic failure - 1 carrier  identified by WGS of men with NOA |  | 31562180;33615715;36017582 |
| FOXL2 | 3q22.3 | 605597 | AD, AR AD, AR AD  AD | 110100  110100  608996  NA | Premature ovarian failure 3 Oligozoospermia in combination with blepharophimosis, corneal vascularization, deafness, and acroosteolysis | Blepharophimosis, epicanthus inversus, and ptosis, type 1 Blepharophimosis, epicanthus inversus, and ptosis, type 2 | 17103436;31823134 |
| FOXM1 | 12p13.33 | 602341 | Undetermin ed | NA - PS 258150 | NOA - identified by WES of GEMINI Cohort |  | 36572685;35743036 |
| FOXP3 | Xp11.23 | 300292 | XLR XL | 304790  NA - PS305700 | Non-obstructive azoospermia | Immunodysregulation, polyendocrinopathy, and enteropathy, X-linked | 31855573 |
| FSIP2 | 2q32.1 | 615796 | AR | 618153 | Spermatogenic failure 34 | n.r. | 30137358;30686508;30745215;33631238;34935173;36632462 |
| FTHL17 | Xp21.2 | 300308 | XL | NA - PS305700 | Azoospermia |  | 14531651 |
| FTO | 16q12.2 | 610966 | AR AR AR | 612460  612938  NA - PS258150 | Oligozoospermia | {Obesity, susceptibility to, BMIQ14}  Growth retardation, developmental delay, facial dysmorphism | 26820768 |

| Gene symbol | Locus | OMIM | Inheritance | Phenotype  MIM | GU Phenotype(s) | Other associated  phenotypes | Reference(s) |
| --- | --- | --- | --- | --- | --- | --- | --- |
| FUS | 16p11.2 | 137070 | AD AD | 608030  614782  NA - PS258150 | Non-obstructive azoospermia | Amyotrophic lateral sclerosis 6, with or without frontotemporal dementia Essential tremor, hereditary, 4 | 19369647 |
| GALNTL5 | 7q36.1 | 615133 | ? | n.r. | Asthenozoospermia | n.r. | 24398516; 30628500 |
| GAPDHS | 19q13.12 | 609169 | AR | NA - PS258150 | Multiple morphological abnormalities of the sperm flagella |  | 27135296;21255775;32321348 |
| GAS8 | 16q24.3 | 605178 | AR | 616726 | Ciliary dyskinesia, primary, 33  (male infertility not confirmed) |  | 26387594;27120127;37950557 |
| GCNA | Xq13.1 | 300369 | XL | 301077 | Spermatogenic failure, X- linked, 4 |  | 33963445;34413498;35172124 |
| GFPT2 | 5q25.3 | 603865 | AR | NA -  PS258150 | Asthenozoospermia |  | 30849544 |
| GGCX | 2p11.2 | 137167 | AR AR | 610842  277450  NA - PS258150 | Asthenozoospermia | Pseudoxanthoma elasticum-like disorder with multiple coagulation factor deficiency Vitamin K-dependent clotting factors, combined deficiency of, 1 | 30981116 |
| GGN | 19q13.2 | 609966 | AR | 619826 | Spermatogenic failure 69 (oligozoospermia, globozoospermia) |  | 21114676;31985809;33108537 |
| GJA1 | 6q22.31 | 121014 | AR AD AD AR AD AD  Undetermin  ed | 218400  617525  164200  257850  104100  186100  NA | Oligozoospermia in combination with blepharophimosis, corneal vascularization, deafness, and acroosteolysis | Craniometaphyseal dysplasia, autosomal recessive Erythrokeratodermia variabilis et progressiva 3 Oculodentodigital dysplasia Oculodentodigital dysplasia, autosomal recessive Palmoplantar keratoderma with congenital alopecia Syndactyly, type III | 17103436 |
| GJB2 | 13q12.11 | 121011 | AD AD  AR, DD AD  AD AD AD  AD | 149200  601544  220290  602540  148210  148350  124500  NA | Oligozoospermia in combination with blepharophimosis, corneal vascularization, deafness, and acroosteolysis | Bart-Pumphrey syndrome Deafness, autosomal dominant 3A  Deafness, autosomal recessive 1A  Hystrix-like icthyosis with deafness  Keratitis-ichthyosis-deafness syndrome  Keratoderma, palmoplantar, with deafness  Vohwinkel syndrome | 17103436 |
| GJB6 | 13q12.11 | 604418 | AD AR  AR, DD AD  Undetermin  ed | 612643  612645  220290  129500  NA | Oligozoospermia in combination with blepharophimosis, corneal vascularization, deafness, and acroosteolysis | Deafness, autosomal dominant 3B Deafness, autosomal recessive !b Deafness, digenic GJB2/GJB6  Ectodermal dysplasia 2, Clouston type | 17103436 |
| GOPC | 6q22.1 | 606845 | AR | NA - PS 258150 | Globozoospermia |  | 16400072 |
| GPX4 | 19p13.3 | 138322 | AR | 250220  NA - PS 258150 | Oligoasthenozoospermia | Spondylometaphyseal dysplasia, Sedaghatian type | 16872467;12606444 |
| GTF2H3 | 12q24.31 | 601750 | AR | NA - PS258150 | Non-obstructive azoospermia |  | 29966603 |
| GTSE1 | 22q13.31 | 607477 | Undetermin  ed | NA - PS  258150 | NOA - identified by WES of  GEMINI Cohort |  | 36572685 |
| H1-7 | 12q13.11 | 618565 | AR | NA - PS 258150 | Azoospermia and oligozoospermia |  | 17644991;16533358;31377750 |
| H19 | 11p15.5 | 103280 | Undetermin  ed | NA | Sertoli cell only synrome |  | 30478911 |

| Gene symbol | Locus | OMIM | Inheritance | Phenotype  MIM | GU Phenotype(s) | Other associated  phenotypes | Reference(s) |
| --- | --- | --- | --- | --- | --- | --- | --- |
| H2AX | 11q23.3 | 601772 | Undetermin ed | NA - PS258150 | Spermatogenic failure |  | 18536151 |
| H2BW1 | Xq22.2 | 300507 | XL | NA -  PS305700 | Non-obstructive azoospermia |  | 32376790 |
| HASPIN | 17p13.2 | 609240 | Undetermin ed | NA | Male infertility |  | 17644991 |
| HAUS7 | Xq28 | 300540 | XL | NA -  PS258150 | Severe oligozoospermia |  | 29017965;32469048 |
| HCN4 | 15q25.1 | 605206 | AD AD  Undetermin ed | 619521  613123  163800  NA - PS 258150 | NOA - identified by WES + 2 stage burden testing vs fertile controls | {Epilepsy, idiopathic generalized, susceptibility to, 18}  Brugada syndrome 8 Sick sinus syndrome 2 | 36572685 |
| HENMT1 | 1p13.3 | 612178 | Undetermin ed | NA - PS 258150 | Spermatogenic failure - identified by WES of men with NOA |  | 35172124;34590701 |
| HFM1 | 1p22.2 | 615684 | AR | 615724  NA - PS 258150 | Premature ovarian failure 9 Spermatogenic failure |  | 37574498;36864181;35526155;35486194;35172124;34429122; |
| HLA-DPB1 | 6p21.32 | 142858 | Undetermin ed | NA  NA - PS 258150 | Non-obstructive azoospermia | {Beryllium disease, chronic, susceptibility to} | 31377750 |
| HLA-DQB1 | 6p21.32 | 604305 | AR, Mu AD  Mu  Undetermin ed | 212750  123400  126200  NA - PS 258150 | Non-obstructive azoospermia | {Celiac disease, susceptibility to}  {Creutzfeldt-Jakob disease, variant, resistance to}  {Multiple sclerosis, susceptibility to, 1} | 31377750 |
| HLA-DRB1 | 6p21.32 | 142857 | Mu AD  Undetermin  ed | 126200  181000  NA - PS258150 | Non-obstructive azoospermia | {Multiple sclerosis, susceptibility to, 1}  {Sarcoidosis, suceptibility to, 1} | 31377750 |
| HMGB2 | 4q34.1 | 163906 | Undetermin ed |  | Testicular failure |  | 19369647 |
| HOOK1 | 1p32.1 | 607820 | AR | NA | Acephalic sperm |  | 29330334 |
| HORMAD1 | 1q21.3 | 609824 | Undetermin ed | NA - PS 258150 | NOA - identified by WES of a large Turkish cosanguineous family (7 carriers including 3 infertile brothers, 2 fertile brothers, 2 parents) |  | 36524333;22407170 |
| HSD17B4 | 5q23.1 | 601860 | AR | NA - PS 258150  261515  233400 | Non-Obstructive Azoospermia - per Houston et. al. | D-bifunctional protein deficiency  Perrault syndrome 1 | 31377750;24602372 |
| HSF2 | 6q22.31 | 140581 | ? | n.r. | Mou et al (2013) reported nonobstructive azoospermia due to blockage of the spermatogenic process primarily at the spermatocyte stage in a Chinese male with heterozygous variant in HSF2. | n.r. | 23064888; 32655042 |
| HSFY1 | Yq11.222 | 400029 | YL | NA - PS 400042 | Azoospermia |  | 15734897;15044259 |
| HYDIN | 16q22.2 | 610812 | AR | 608647 | Ciliary dyskinesia, primary, 5 (sperm flagella show reduced coordination of beating activity) MMAF asthenoteratozoospermia due to absence of central pair associated protein SPEF2 from flagellar axonemes according to Aprea et. al 2023 | Ciliary dyskinesia, primary, 28, with or without situs inversus, and associated sinopulmonary phenotypes | 23022101; 36742411; 36873931 |
| IFT140 | 16p13.3 | 614620 | AR | NA - PS 258150  617781  266920 | Oligoasthenoteratozoospermia per Houston et. al. 2020 | Retinitis pigmentosa 80 Short-rib thoracic dysplasia 9 with or without polydactly | 31397098, 38084016 |
| IGSF1 | Xq26.1 | 300137 | XLR  Undetermin ed | 300888  NA - 305700 | NOA - identified by WGS of men with NOA | Hypothyroidism, central, and testicular enlargement | 36017582 |
| IGF2R | 6q25.3 | 147280 | Undetermin ed |  | Non-obstructive azoospermia - per Houston et. al. 2020 |  | 31377750 |

| Gene symbol | Locus | OMIM | Inheritance | Phenotype  MIM | GU Phenotype(s) | Other associated  phenotypes | Reference(s) |
| --- | --- | --- | --- | --- | --- | --- | --- |
| ING2 | 4q35.1 | 604215 | AR |  | Non-obstructive azoospermia per Houston et. al. 2020 |  | 31377750, 21124960 |
| INSL6 | 9p24.1 | 606414 | AR | NA -  PS258150 | Asthenozoospermia/cryptorchidis  m per Houston et. al. 2020 |  | 25877373;21616179 |
| IZUMO1 | 19q13.33 | 609278 | AR | NA | Fertilization failure |  | 18,082,733 |
| KASH5 | 19q13.33 | 618125 | AR |  | n.r.  spermatocyte maturation arrest seen in knockout mice | n.r. | 29790874; 33980926; 35587281; 35674372; 36864840 |
| KATNAL1 | 13q12.3 | 614764 | AR | NA - PS258150 | Non-obstructive azoospermia - per Houston et. al. 2020 |  | 24913027 |
| KATNB1 | 16q21 | 602703 | AR | NA -  PS258150 | Oligoazoospermia - per Houston  et. al. 2020 |  | 25280067;34822718 |
| KCND1 | Xp11.23 | 300281 | Undetermin ed | NA - PS258150 | Spermatogenic failure - 2 carries identified by WGS of men with NOA |  | 36017582; |
| KCNQ1 | 11p15.1-p15.4 | 607542 | AD AD AR AD AD  Undetermin  ed | 192500  607554  220400  192500  609621  NA - PS258150 | Sertoli cell only syndrome - per Houston et. al. 2020 | {Long QT syndrome 1, acquired, susceptibility to} Atrial fibrillaation, familial, 3 Jervell and Lange-Nielsen syndrome  Long QT syndorme 1 Short QT syndrome 2 | 30478911 |
| KCTD19 | 16q22.1 | 619943 | Undetermin ed | NA - PS258150 | NOA |  | 36572685; 37485353; 37257643; 37192818 |
| KDM3A | 2p11.2 | 611512 | AD | NA -  PS258150 | Oligozoospermia - per Houston  et. al. 2020 |  | 30053768 |
| KIAA1210 | Xq24 | 300995 | Undetermin ed | NA - PS258150 | Spermatogenic failure - 2 carriers identified by WGS of men with NOA |  | 36017582;34791246 |
| KIF2C | 1p34.1 | 604538 | AR | NA -  PS258150 | Non-obstructive azoospermia -  per Houston et. al. 2020 |  | 31377750 |
| KISS1R | 19p13.3 | 604161 | AR | NA - PS 258150 | Azoospermia per Houston et. al. 2020 |  | 27544332; 25739677; 22035731; 29452377; 29264451; 28833369; 27094476; 26199944; 26031747; 25636053; 25262569;  25064402; 24732674; 24522099; 23643382; 23349759; 22766261; 22724017; 22619348; 21193544; 20371656; 18463157;  17164310; 17179725; 17074994; 16322390; 12944565; 14573733; 30098700; 31885997; 31821609; 31377750; 31073722;  30669598; 30669598; 15598687; 31821609; 37814704; 35735778; 35133534; 33819414; 33270637; |
| KIT | 4q12 | 164920 | AD | 606764  273300  601626  154800  154800  172800  NA - PS 258150 | Azoospermia per Houston et. al. 2020 | Gastrointestinal stromal tumor, familial  Germ cell tumors, somatic Leukemia, acute myeloid, somatic  Mastocytosis, cutaneous Mastocytosis, systemic, somatic  Piebaldism | 12322893 |
| KLHL10 | 17q21.2 | 608778 | AD | 615081 | Spermatogenic failure 11 (oligozoospermia, teratozoospermia in some patients, asthenozoospermia in some patients) | n.r. | 17047026; 31377750; 31479588; 32242295; 32655042; 34433733 |
| KLK12 | 19q13.41 | 605539 | Undetermin  ed | NA | Possibly involved in male inferility  - per Marques et. al. 2016 |  | 27827323 |
| KLK14 | 19q13.41 | 606135 | AR | NA | Possibly involved in male inferility  - per Marques et. al. 2016 |  | 27827323 |
| KLK15 | 19q13.33 | 610601 | Undetermin ed | NA | Possibly involved in male inferility  - per Marques et. al. 2016 |  | 27827323 |
| KLK3 (PSA) | 19q13.33 | 176820 | Undetermin  ed | NA | Possibly involved in male inferility  - per Marques et. al. 2016 |  | 27827323;35768906;28894123 |
| KLK4 | 19q13.41 | 603767 | AR AR | 204700  NA | Possibly involved in male inferility  - per Marques et. al. 2016 | Amelogenesis imperfecta, type IIA1 | 27827323 |
| KLK6 | 19q13.41 | 602652 | Undetermin ed | NA | Possibly involved in male inferility  - per Marques et. al. 2016 |  | 27827323 |
| KLK7 | 19q13.41 | 604438 | Undetermin  ed | NA | Possibly involved in male inferility  - per Marques et. al. 2016 |  | 27827323 |
| KLK8 | 19q13.41 | 605644 | AR | NA | Possibly involved in male inferility  - per Marques et. al. 2016 |  | 27827323 |

| Gene symbol | Locus | OMIM | Inheritance | Phenotype  MIM | GU Phenotype(s) | Other associated  phenotypes | Reference(s) |
| --- | --- | --- | --- | --- | --- | --- | --- |
| LHX4 | 1q25.2 | 602146 | Undetermin ed | NA - PS258150 | Non-Obstructive Azoospermia - per Houston et. al. 2020 |  | 25064402;31377750;30888394;35805171;33098107 |
| LIMK2 | 22q12.2 | 601988 | AR | NA -PS258150 | Testicular failure - per Houston et. al. 2020 |  | 19369647 |
| LIPE | 19q13.2 | 151750 | AR AR | 615980  NA -PS258150 | Testicular failure - per Houston et. al. 2020 | Lipodystroiphy, familial partial, type 6 | 19369647 |
| LRRC6 | 8q24.22 | 614930 | AR | 614935 | Ciliary dyskinesia, primary, 19, with or without situs inversus | primary ciliary dyskinesia 19, with or without situs inversus, and associated sinopulmonary phenotypes | 23122589; 23891469; 36515799; 35768906; 33635866; 29511670; 33988008; 33403504 |
| LRWD1 | 7q22.1 | 615167 | Undetermin  ed | NA -  PS258150 | Sertoli cell only synrdome - per  Houston et. al. 2020 |  | 23445371 |
| LSM6 | 4q31.22 | 607286 | AD | NA - PS258150 | Azoospermia - per Houston et. al. 2020 |  | 18042180 |
| M1AP | 2p13.1 | 619098 | AR | 619108 | Spermatogenic failure 48 | n.r. | 32673564; 35341049; 36572685 |
| MAEL | 1q24.1 | 611368 | AR | NA - PS258150 | Non-obstructive azoospermia - per Houston et. al. 2020 |  | 31377750, 39122675 |
| MAGEB4 | Xp21.2 | 300153 | XL | NA -  PS305700 | Spermatogenic failure, X-linked,  variant of unknown significance |  | 28401488 |
| MAGEB6 | Xp21.3 | 300467 | XL | NA - PS305700 | Spermatogenic failure - 1 carrier identified by WGS of men with NOA |  | 36017582 |
| MAJIN | 11q13.1 | 617130 | AR | NA - PS258150 | association pending confirmation  - NOA - an infertile man of Syrian ancestry was found to be homozygous for an R53H missense variant in Majin  degenerated testis and ovaries seen in Majin -/- mice | n.r. | 33211200; 35342767 |
| MAP3K15 | Wp22.12 | 300820 | XL | NA - PS305700 | Spermatogenic failure - 3 carriers identified by WGS of men with NOA |  | 36017582 |
| MAP7D3 | Xq26.2 | 300930 | XL | NA -  PS258150 | Non-obstructive azoospermia -  per Houston et. al. 2020 |  | 32376790 |
| MCIDAS | 5q11.2 | 614086 | AR | 618695 | Ciliary dyskinesia, primary, 42 (associated with female infertility)  male infertility seen in mice | ciliary dyskinesia, primary, 42; female infertility, sinopulmonary phenotypes associated with ciliary dyskinesia, enlarged ventricles (mild, due to arrested hydrocephalus) | 1523039; 8813877; 25048963; 30237576; 34569065 |
| MCM8 | 20p12.3 | 608187 | AR | 612885 | ?Premature ovarian failure 10? | n.r. | 25873734; 35172124 |
| MCM9 | 6p22.31 | 610098 | AR AR | 616185  NA - PS258150 | Ovarian dysgenesis 4  NOA - per Houston et. al. 2020 |  | 32469048;36769638 |
| MCMDC2 | 8q13.1 | 617545 | Undetermin ed | NA - PS 258150 | Spermatogenic failure - identified by WES of men with NOA |  | 35172124; |
| MDC1 | 6p21.33 | 607593 |  | NA - PS  617576 | Multiple morphological  abnormalities of sperm |  | 34089056 |
| MEI1 | 22q13.2 | 608797 | AR | 618431 | one report of nonobstructive azoospermia in one male who was compound heterozygous for an invariant splice site mutation and a 1-bp deletion  several WES findings - see  sources | Hyatidiform mole | 32741963; 29659827; 30388401; 35413094; 36759719 |
| MEIOB | 16p13.3 | 617670 | AR | 617706 | Spermatogenic failure 22 | n.r. | 32741963; 28206990; 30838384; 35172124 |
| MEIG1 | 10p13 | 614174 | AR | NA - PS258150 | Azoospermia and oligozoospermia per Houston et. al. 2020 |  | 22673690 |
| MKKS | 20p12.2 | 604896 | AR AR | 605231  236700 | Bardet-Biedl syndrome 6 (hypogenitalism, hypospadias, structured renal abnoramlities, lobulated kidneys, cystic kidneys) McKusick-Kaufman syndrome (cryptorchidism) | Bardet-Biedl syndrome 6 McKusick-Kaufman syndrome | 10802661; 10973238; 10973251; 12677556; 12837689; 16104012; 15770229; 20120035; 20472660; 21044901; 24608809;  24849935; 33520300 |

| Gene symbol | Locus | OMIM | Inheritance | Phenotype  MIM | GU Phenotype(s) | Other associated  phenotypes | Reference(s) |
| --- | --- | --- | --- | --- | --- | --- | --- |
| MKS1 | 17q22 | 609883 | AR AR AR | 615990  617121  249000 | Bardet-Biedl syndrome 13  Merckel syndrome 1 | Joubert syndrome 28 | 18327255; 24608809 |
| MLH3 | 14q24.3 | 604395 | AD, SMu  AR (likely - according to DOMINO) | 608089  614385  114500  NA - PS258150 | NOA | {Endometrial cancer, susceptibility to}  Colorectal cancer, hereditary nonpolyposis, type 7 Colorectal cancer, somatic | 17482610;32469048;36041235;34408140;33517345 |
| MNS1 | 15q21.3 | 610776 | AR | 618948 | Heterotaxy, visceral, 9, autosomal, with male infertility (abnormal sperm morphology, short tail on sperm, imparied progressive sperm motility) |  | 30148830; 31534215; 33037173 |
| MSH4 | 1p31.1 | 602105 | AR | 619936  108420 | Premature ovarian failure 20  Spermatogenic failure 2 | n.r. | 21126912; 28718531; 28541421; 33448284; 33437391; 34755185; 32741963; 35090489; 37459509 |
| MSH5 | 6p21.33 | 603382 | AR AR | 617442  619937 | ?Premature ovarian failure 13? Spermatogenic failure 74 |  | 34755185;36259570;35742973;35172124;34980881;22594646; |
| NANOS1 | 10q26.11 | 608226 | AD | 615413 | Spermatogenic failure 12 (Azoospermia, sertoli cell-only seminiferous tubule histology, oligoasthenoteratozoospermia (noted in 1 patient)) | n.r. | 23315541; 32155011; 32242295; 32655042; 35922383 |
| NANOS2 | 19q13.32 | 608228 | AR |  | n.r.  Nanos2-null mice were viable and showed no apparent abnormalities, but Nanos2-null testes had defects in spermatogenesis. | n.r. | 23315541; 29790874 |
| NGFRAP3 | Xq22.2 | 300361 | Undetermin ed | NA - PS305700 | Non-obstructive azoospermia per Houston et. al. 2020 |  | 36943184;14531651 |
| NLRP3 | 1q44 | 606416 | AD AD AD AD AD | 607115  617772  120100  148200  191900 | Muckle-Wells syndrome (Infertility secondary to inflammasome activation - Tavalaee et. al. 2022) | CINCA syndrome Deafness, autosomal dominant 34, with or without inflammation  Familial cold inflammatory syndrome 1 Keratoendothelitis fugax hereditaria | 22512814;37491934;37476898;36177957;35637440;35563625 |
| NOTCH1 | 9p34.3 | 190198 | AD AD  Undetermin  ed | 616028  109730  NA - PS258150 | NOA | Adams-Oliver syndrome 5 Aortic valve disease 1 | 36572685;27502037;33208564 |
| NPAS2 | 2q11.2 | 603347 |  |  | associations pending confirmation nonobstructive azoospermia, small testes observed in 3 Turkish brothers homozygous for P455AS missense variation in NPAS2 |  | 25956372 |
| NR0B1 (DAX1) | Xp21.2 | 300473 | XL | PS -NA - PS 305700 | Non-obstructive azoospermia per Houston et. al. 2020 |  | 28741070; 27648561; 26260363; 26207377; 25529318; 23384712; 21227944; 16645015; 16556678; 16275267; 11788621;  10675358; 29176027; 28924487; 28284037; 28075027; 27711951; 27035099; 26537215; 26030781; 25993682; 25968435;  25064402; 25003377; |
| NR5A1 | 9q33.3 | 184757 | AD | 613957 | Spermatogenic failure 8 | adrenocortical insufficiency | 25989977; 24750329; 23299922; 20887963; 24067197 |
| OFD1 | Xq22.2 | 300170 | XLR | ?300424?  300804  311200  300209 | Joubert syndrome 10 (cystic renal disease, increased renal echogenicity)  Orofaciodigital syndrome 1 (ovarian cysts, adult onset polycystic kidney (50%)) Simpson-Golabi-Behmel syndrome, type 2 (inguinal hernia in male) | ?Retinitis pigmentosa 23?  Joubert syndrome 10  Orofaciodigital syndrome 1 Simpson-Golabi-Behmel syndrome, type 2 | 31373179 |

| Gene symbol | Locus | OMIM | Inheritance | Phenotype  MIM | GU Phenotype(s) | Other associated  phenotypes | Reference(s) |
| --- | --- | --- | --- | --- | --- | --- | --- |
| OFD1 | Xq22.2 | 300170 | XLR | ?300424?  300804  311200  300209  NA - PS244400 | Joubert syndrome 10 (cystic renal disease, increased renal echogenicity)  Orofaciodigital syndrome 1 (ovarian cysts, adult onset polycystic kidney (50%)) Simpson-Golabi-Behmel syndrome, type 2 (inguinal hernia in male)  not reported on OMIM - primary ciliary dyskinesia | ?Retinitis pigmentosa 23?  Joubert syndrome 10  Orofaciodigital syndrome 1 Simpson-Golabi-Behmel syndrome, type 2 | 31373179; 31366608; 27957444 |
| PACRG | 6q26 | 608427 | AR | NA - PS258150 | NOA |  | 36572685;19268936;34089056 |
| PCDHB3 | 5q31.3 | 606329 | Undetermin  ed | NA -  PS258150 | NOA |  | 36572685 |
| PDHA2 | 4q22.3 | 179061 | AR | 619828 | Spermatogenic failure 70 |  | 29581481;35172124 |
| PICK1 | 22q13.1 | 605926 |  | n.r. | Globozoospermia  male PICK1 -/- mice completely infertile, phenotype resembled human globozoospermia | n.r. | 20562896; 24265589 |
| PIHID3 | Xq22.3 | 300933 | XLR | 300991 | Ciliary dyskinesia, primary, 36, X-linked (immotile sperm) | Ciliary dyskinesia, primary, 36, X-linked | 32170493;28041644;28176794;33635866 |
| PIWIL1 | 12q24.33 | 605571 |  |  |  |  | 33861958;36017582;31377750;28552346 |
| PKD1 | 16p13.3 | 601313 | AD | 173900 | Polycystic kidney disease 1 (polycystic kidney disease, renal failure)  Asthenozoospermia - per  Houston Review |  | 30333007; 30651829; 34739738; 37152951; 37540677 |
| PKD1 | 16p13.3 | 601313 | AD | 173900 | Polycystic kidney disease 1 (polycystic kidney disease, renal failure)  Azoospermia - per Houston  Review |  | 30333007; 30651829; 34739738; 37152951;37540677 |
| PLA2G6 | 22q13.1 | 603604 | AR AR AR AR | 256600  610217  612953  NA - PS258150 | Asthenoteratozoospermia - per Houston et. al. 2020 | Infantile neuroaxonal dystrophy 1 Neurodegeneration with brain iron accumulation 2B Parkinson disease 14, autosomal recessive | 36572685;21255775 |
| PLCZ1 | 12p12.3 | 608075 | AR | 617214 | Spermatogenic failure 17 (oocyte activation failure, acrosome detached from nuclear envelope, crumpled acrosome, perinuclear theca irregularly thickened) | n.r. | 18924610; 19584136; 22095789; 26721930; 32048714; 32142120; 32146562; 36593593; 36529831; 37261077 |
| PLD6 | 17p11.2 | 614960 | Undetermin  ed | NA -  PS258150 | NOA - 2 carriers within GEMINI  cohort |  | 36572685;36257818;32141383 |
| PLK4 | 4q28.1 | 605031 | AD | 616171 | n.r. | Microcephaly and chorioretinopathy | 26452337; 35366911 |
| PMFBP1 | 16q22.2 | 618085 | AR | 618112 | Spermatogenic failure 31 | n.r. | 36579083; 36017582; 35860846; 33484382; 30032984; 30298696; 32285443 |
| PNLDC1 | 6q25.3 | 619529 | AR | 619528 | Spermatogenic failure 57 (oligoasthenoteratozoospermia, reduced testicular volume, spermatogenic arrest at late pachytene stage, low testosterone in some patients) |  | 37458503; 35476664; 34347949; 37261077; 36572685 |
| POLG | 15q26.1 | 174763 |  |  |  |  |  |
| PPP2R3C | 14q13.2 | 615902 | AR AD | 618419  618420 | Myoectodermal gonadal dysgenesis syndrome (complete gonadal dysgenesis, complete 46,XY female, intrafamilial variability seen - range of ambigous genitalia, unilateral renal agenesis has been seen) Spermatogenic failure 36 | Myoectodermal gonadal dysgenesis syndrome | 30893644;34714774;34750818;35812758;37147882 |
| PRM2 | 16p13.13 | 182890 |  | n.r. | oligozoospermia or azozoospermia | n.r. | 10464636; 12569175; 16989827; 17644991; 18309899; 19602509; 19863670; 21425891; 22104739; 31377750; 36197138 |
| PRM2 | 16p13.13 | 182890 |  | n.r. | teratozoospermia | n.r. | 10464636; 12569175; 16989827; 17644991; 18309899; 19602509; 19863670; 21425891; 22104739; 31377750; 36197138 |

| Gene symbol | Locus | OMIM | Inheritance | Phenotype  MIM | GU Phenotype(s) | Other associated  phenotypes | Reference(s) |
| --- | --- | --- | --- | --- | --- | --- | --- |
| PSMC3IP | 17q21.2 | 608665 | AR | 614326 | Ovarian dysgenesis  "In a consanguineous Yemeni family, a homozygous PSMC3IP stop gain mutation deleting the C-terminal portion of the protein has been found to cosegregate with POI and NOA phenotypes" cioppi et al | Ovarian dysgenesis | 29240891 |
| QRICH2 | 17q25.1 | 618304 | AR | 618341 | Spermatogenic failure 35 (MMAF) | n.r. | 30683861; 31292949; 34089056; 36017582 |
| QRICH2 | 17q25.1 | 618304 | AR | n.r. | Azoospermia - per Houston Review PS258150 | n.r. | 30683861; 31292949; 34089056; 36017582 |
| RAD21L1 | 20p13 | NA | AR |  | n.r.  RAD21L1 -/- zebrafish were predominantly male due to late female to male sex reversal | n.r. | 32741963;28635411;34138874 |
| RBMXL3 | Xq23 | NA | XL | NA - PS 305700 | Spermatogenic failure - 2 carriers identified by WGS of men with NOA |  | 36017582 |
| REC8 | 14q12 | 608193 | Undetermin  ed | NA - PS  258150 | Spermatogenic failure |  | 35172124;18570052;31479588 |
| RNF17 | 13q12.12 | 605793 | Undetermin ed | NA - PS 258150 | NOA - 1 case identified by WES of GEMINI cohort |  | 36572685;25739334 |
| RNF212 | 4p16.3 | 612041 | AR | ?619673?  612042 | ?spermatogenic failure 62?  Recombination rate QTL 1 | n.r. | 29277047; 31125047; 37124137 |
| RPL10L | 14q21.2 | 619689 | AR | 619689 | ?Spermatogenic failure 63? | n.r. | 32111475 |
| RSPH1 | 21q22.3 | 609314 | AR | 615481 | Ciliary dyskinesia, primary, 24 male and female infertility Dysmotile sperm - abnormal radial spoke head formation - according to Aprea et. al 2023 | Ciliary dyskinesia, primary, 24, without situs inversus, and associated sinopulmonary phenotypes | 23993197; 24518672; 36873931; 25877373 |
| RSPH3 | 6q25.2 | 615876 | AR | 615876 | Ciliary dyskinesia, primary, 32 | Ciliary dyskinesia, primary, 32, without situs inversus, and associated  sinopulmonary phenotypes | 26073779; 32124190 |
| RSPH4A | 6q22.1 | 612647 | AR | 612649 | Ciliary dyskinesia, primary, 11, without situs inversus  (male infertility not confirmed) | Ciliary dyskinesia, primary, 11, without situs inversus, and associated sinopulmonary phenotypes | 19200523; 23993197; 23798057; 34513534; 35812741 |
| RSPH9 | 6q21.1 | 612648 | AR | 612650 | Ciliary dyskinesia, primary, 12, without situs inversus  male (dysmotile sperm) and female infertility  Dysmotile sperm - abnormal  radial spoke head formation - according to Aprea et. al 2023 | Ciliary dyskinesia, primary, 12, without situs inversus, and associated sinopulmonary phenotypes | 19200523; 31285900; 23993197; 36873931 |
| SEMA5A | 5p15.31 | 609297 | Undetermin ed | NA - PS 258150 | 1 carrier (de novo mutation) identified by trio-WES of men with idiopathic azoospermia and their parents |  | 32860660 |
| SEPTIN12 | 16p13.3 | 611562 | AD | 614822 | Spermatogenic failure 10 (teratozoospermia (in some patients), asthenoteratozoospremia (in some patients), oligoasthenozoospermia (in some patients)  MMAF - per Houston Review | n.r. | 21636737; 22116646; 22479503; 22275165; 30488758; 30513371; 31377750; 31880374; 32242295; 34057684; 34791246;  35547809 |
| SEPTIN12 | 16p13.3 | 611562 | AD |  | NOA - per Houston Review | n.r. | 21636737; 22116646; 22479503; 22275165; 30488758; 30513371; 31377750; 31880374; 32242295; 34057684; 34791246;  35547809 |
| SETX | 9q34.13 | 608465 | AR | 602433  606002 | n.r.  "In the two articles in which testis histology has been described, AOA2 male patients exhibited MA at primary spermatocyte stage" cioppi et al | Amyotrophic lateral sclerosis 4, juvenile; Spinocerebellar ataxia, autosomal recessive, with axonal neuropathy 2 | 24637776; 30778901; 30642639 |
| SH3TC2 | 5q32 | 608206 | AR AD  Undetermin  ed | 601596  613353  NA - PS258150 | NOA - 2 carriers within GEMINI cohort | Charcot-Marie-Tooth disease, type 4c Mononeuropathy of the median nerve, mild | 36572685 |
| SHOC1 | 9q31.3 | 618038 | AR | 619949 | Spermatogenic failure 75 | n.r. | 32741963; 32900840; 35485979 |

| Gene symbol | Locus | OMIM | Inheritance | Phenotype  MIM | GU Phenotype(s) | Other associated  phenotypes | Reference(s) |
| --- | --- | --- | --- | --- | --- | --- | --- |
| SIN3A | 15q24.2 | 607776 | AD | NA - PS 258150 | azoospermia |  | 25395209;22820070;34009138;36758531 |
| SLC10A7 | 4q31.22 | 611459 | AR  Undetermin ed | 618363  NA | Hypergonadotropic hypogonadism | Short stature, amelogenesis imperfecta, and skeletal  dysplasia with scoliosis | 18042180 |
| SLC26A8 | 6p21.31 | 608480 | AD, AR | 606766 | Spermatogenic failure 3 (asthenozoospermia) | n.r. | 15579655; 23582645; 34923715; 35181959 |
| SLC29A3 | 10q22.1 | 612373 | AR | 602782 | Histiocytosis-lymphanopathy plus syndrome, phenotype highly variable  (hypergonadotropic hypogonadism, hypogonadotropic  hypogonadism (rare)) | Histiocytosis-lymphanopathy plus syndrome, phenotype highly variable | 18940313; 20619369 |
| SLC4A2 | 7q36.1 | 109280 | AR  Undetermin ed | 620366  NA - PS258150 | NOA - 2 carriers within GEMINI cohort | ?osteoporosis, autosomal recessive 9? | 36572685 |
| SLX4 | 16p13.3 | 613278 | AR | 613951  NA - PS 258150 | NOA | Fanconi anemia | 36041235;36880652 |
| SOHLH1 | 9q34.3 | 610224 | AD | 617690  618115 | Spermatogenic failure 32  Ovarian dysgenesis 5 |  | 20506135; 25463635; 28718531; 32655042 |
| SOHLH2 | 13q13.3 | 616066 |  |  | n.r.  Sohlh2-null mice were infertile due to a block in spermatogenesis. Although normal prior to birth, male Sohlh2-null mice had reduced  numbers of intermediate and type B spermatogonia by postnatal day 7. By day 10, progression to the preleptotene spermatocyte stage was severely disrupted, resulting in seminiferous tubules with only Sertoli cells, undifferentiated spermatogonia, and degenerating colonies of  differentiating spermatogonia | n.r. | 25463635; 18339773 |
| SOX10 | 22q13.1 | 602229 | AD AD AD  Undetermin  ed | 609136  611584  613266  NA - PS258150 | Non-obstructive azoospermia - per Houston et. al. 2020 | PCWH syndrome Waardenburg syndrome, type 2E, with or without neurologic involvement Waardenburg syndrome, type 4C (olfactory bulb agenesis in some patients) | 25529318; 29678855; 26228106; 24769923; 23643381; 31377750; 30914325; 30669598; 32400067; 37814704; 37799300;  36927561; 35133534; 34766489; 34095692; 33913437; 33597923; 33442024; 32908489; 32763379 |
| SPACDR | 7q22.1 | 619782 |  | NA - PS 258150 | Variant of unknown significance - spermatogenic failure (globozoospermia and acrosomal hypoplasia) |  | 31985809 |
| SPAG1 | 8q22.2 | 603395 | AR | 615505 | Ciliary dyskinesia, primary, 28 (not directy linked to poor sperm mobility via OMIM)  may cause sperm agglutination  and/or immobilization | Ciliary dyskinesia, primary, 28, with or without situs inversus, and associated sinopulmonary phenotypes | 24055112 |
| SPAG17 |  |  |  |  | ?Spermatogenic failure 55 ? |  | 28548327 |
| SPAST | 2p22.3 | 604277 | AD  Undetermin ed | 182601  NA | SPAST knockout mice showed complete loss of functional germ cells, sperm phenotype was consistent with meiotic failure | Spastic paraplegia 4, autosomal dominant | 36971361 |
| SPATA16 | 3q26.31 | 609856 | AR | 102530 | ?Spermatogenic failure 6? (Oligoasthenoteratozoospermia, reduced or absent acrosin in acrosome) | n.r. | 17847006; 21857011; 24265589; 24825417; 27086357; 30912172; 33877510 |
| SPATA20 | 17q21.33 | 613939 | Undetermin  ed | NA -  PS258150 | Spermatogenic failure - 2 carriers  within GEMINI cohort |  | 36572685;36537262;36415156 |
| SPEF2 | 5p13.2 | 610172 | AR | 618751 | Spermatogenic failure 43  MMAF asthenoteratozoospermia | n.r. | 31048344; 31151990; 31278745; 31942643; 34755699; 36873931 |
| SPEF2 | 5p13.2 | 610172 | AR | 618751 | Primary ciliary dyskinesia with MMAF | n.r. | 31048344; 31151990; 31278745; 31942643; 34755699; 36873931 |
| SPINK2 | 4q12 | 605753 | AR | ?618091? | ?Spermatogenic failure 29? | n.r. | 9851748; 28554943 |

| Gene symbol | Locus | OMIM | Inheritance | Phenotype  MIM | GU Phenotype(s) | Other associated  phenotypes | Reference(s) |
| --- | --- | --- | --- | --- | --- | --- | --- |
| SPO11 | 20q13.31 | 605114 | AR |  | n.r.  Mice homozygous for disruption in Spo11 had spermatocytes which arrested prior to the pachytene stage with little or no synapsis and then underwent apoptosis | n.r. | 29790874; 38287033 |
| SPPL2C | 17q21.31 | 608284 | Undetermin ed | NA - PS 617576 | Multiple morphological abnormalities of sperm |  | 34089056 |
| SRY | Yp11.2 | 480000 | YL, XLD | NA - PS 258150 | Isolated oligoasthenoteratozoospermia or azoospermia; OMIM:NA (PS258150) - per Houston et. al. 2020 | tall stature, gynecomastia, gonadoblastoma | 28379671; 27882599; 27899089; 27648416; 26690523; 25374395; 25169080; 24496683; 24379036; 23998093; 23380608;  23378127; 23290744 |
| SSX3 | Xp11.3 | 300325 | XL | NA - PS305700 | Spermatogenic failure - 2 carriers identified by WGS of men with  NOA |  | 36017582 |
| STAG3 | 7q22.1 | 608489 | AR | 615723  619672 | Premature ovarian failure 8 Spermatogenic failure 61 | n.r. | 32741963; 31125047; 31115363; 32634216; 31682730 |
| STK36 | 2q35 | 607652 | AR | 619436 | ?Ciliary dyskinesia, primary, 46? | n.r. | 28543983 |
| STX2 | 12q24.33 | 132350 | AR | NA | Association pending confirmation  - maturation arrest with multinucleated spermatocytes in 1 23 year old male with bilateral small testes, normal testosterone and LH, mildly elevated FSH - NOTE - this same patient was also found to carry 2 rare variants in the SPATA7 and MEI1 genes STX2 -/- mice were sterile due to abnormal testicular development and impaired spermatogenesis | n.r. | 29570232 |
| SUN5 | 20q11.21 | 613942 | AR | 617187 | Spermatogenic failure 16 (oligozoospermia, asthenospermia, acephalic sperm) | n.r. | 27912045; 28541472; 29298896; 29329387; 29331481; 32285443; 33671757; 34159570 |
| SYCE1 | 10q26.3 | 611486 | AR | 616947  616950 | ?Premature ovarian failure 12?  ?Spermatogenic failure 15? | n.r. | 25899990; 30478911; 32741963; 31916078 |
| SYCE1L | 16q23.1 | 619954 |  |  | Non obstructive azoospermia per Houston Review |  | 31479588 |
| SYCP2 | 20q13.33 | 604105 | AD | 258150 | Spermatogenic failure 1 | n.r. | 31866047; 37337432 |
| SYCP3 | 12q23.2 | 604759 | AD | 270960 | Pregnancy loss, recurrent, 4 (females)  Spermatogenic failure 4 (males) | n.r. | 14643120; 16213863; 17434513; 21126912; 22670862; 28801929; 32242295; 32655042 |
| TAF4 | 20q13.33 | 601796 | AD | 620450  NA - PS258150 | Spermatogenic failure - 1 carrier identified by WGS of men with NOA |  | 36017582 |
| TAF4B | 18q11.2 | 601689 | AR | ?615841? | ?Spermatogenic failure 13? | n.r. | 24431330; 31377750; 32502024 |
| TDRD12 | 19q13.11 | N/A | unclear | PS - 258150 | NOA - 1 carrier identified by WES of GEMINI cohort |  | 36572685;32059713;30257204 |
| TDRD7 | 9q22.33 | 611258 | AR | 613887 | n.r.  Tdrd7 - null mice had arrest of spermatogenesis | Cataract 36 | 29532805; 31048812 |
| TDRD9 | 14q32.33 | 617963 | AR | ?618110? | ?Spermatogenic failure 30? | n.r. | 28536242;35172124;36572685;32059713 |
| TDRKH | 1q21.3 | 609501 | Undetermin ed | PS - 258150 | NOA |  | 36572685;35172124 |
| TEKT5 | 16p13.13 | 618686 | AR (likely - according to  DOMINO) | PS - 258150 | NOA |  | 36041235 |
| TERB1 | 16q22.1 | 617332 | AR | 619646 | Spermatogenic failure 60 | n.r. | 32741963; 32719396; 33211200; 35172124 |
| TERB2 | 15q21.1 | 617131 | AR | ?619645? | ?spermatogenic failure 59? | n.r. | 33211200 |
| TEX11 | Xq13.1 | 300311 | XLR | 309120 | Sermatogenic failure, X-linked 2 | n.r. | 32741963; 29661171; 26136358; 25970010 |
| TEX13A | Xq22.3 | 300312 | XL | NA - PS305700 | Spermatogenic failure, X-linked |  | 37594251;36017582;14531651 |
| TEX14 | 17q22 | 605792 | AR | 617707 | Spermatogenic failure 23 | n.r. | 32741963; 29790874; 28206990; 31479588; 33728612; 36017582 |

| Gene symbol | Locus | OMIM | Inheritance | Phenotype  MIM | GU Phenotype(s) | Other associated  phenotypes | Reference(s) |
| --- | --- | --- | --- | --- | --- | --- | --- |
| TEX15 | 8p12 | 605795 | AR | 617960 | Spermatogenic failure 25 | n.r. | 28355598; 26199321; 37234866; 36589743; 32655042 |
| TKTL1 | Xq28 | 300044 | Undetermin ed | NA - PS258150 | NOA - 2 carriers identified by WGS - 1 with MA, 1 with Premeiotic arrest |  | 36017582 |
| TPTE2 | 13q12.11 | 606791 | Undetermin  ed | NA - PS  617576 | Multiple morphological  abnormalities of sperm |  | 34089056 |
| TRIM32 | 9q33.11 | 602290 | AR AR | ?615988?  254110 | ?**Bardet-Biedl syndrome** 11? (hypogonadism, renal anomalies) | Muscular dystrophy, limb-girdle, autosomal recessive 8 | 16606853 |
| TRIM37 | 17q22 | 605073 | AR | 253250 | Mulibrey nanism | Mulibrey nanism | 21865362;29860321 |
| TSGA10 | 2q11.2 | 607166 | AR | 617961 | ?Spermatogenic failure 26? (Low sperm motility, acephalic spermatozoa  , beakage in midpiece of sperm, microcephalic spermatozoa (rare), round-headed  spermatozoa (rare)) |  | 28905369; 32285443; 32410354; 34089195; 34232471; 34409526 |
| TSSK4 | 14q12 | 610711 | Undetermin ed | NA - PS258150 | Azoospermia and aoligozoospermia - per Houston et. al. 2020 |  | 18390560;36572685;36048845 |
| TTC12 | 11q23.2 | 610732 | AR | 618801 | Ciliary dyskinesia, primary, 45  (with infertility) | n.r. | 31978331; 37325566 |
| TTC21A | 3p22.2 | 611430 | AR | 618429 | Spermatogenic failure 37 (MMAF) | n.r. | 30929735; 35920310 |
| TTC29 | 4q31.22 | 618735 | AR | 618745 | Spermatogenic failure 42 (MMAF) | n.r. | 31735292; 31735294; 34529793; 36346162 |
| TTC8 | 14q31.3 | 608132 | AR | ?613464?  615985 | Bardet-Biedle syndrome 8 (hypogodanism, hypospadias, renal dysplasia (in some patients)) | ?Retinitis pigmentosa 51?  Bardet-Biedl syndrome 8 | 14520415; 19402160; 16308660; 16877420; 21044901; 26518167; 28761321 |
| TTLL9 | 20q11.21 | 619838 | Undetermin  ed | NA -  PS258150 | Spermatogenic failure - 2 carriers  within GEMINI cohort |  | 36572685;27257088;3402806 |
| TUBA3C | 13q12.11 | 602528 | Undetermin ed | NA - PS258150 | Spermatogenic failure - 1 carrier within GEMINI cohort; 1 carrier from MERGE |  | 36572685;24268707;22125161 |
| U2AF1 | 21q22.3 | 191317 | Undetermin  ed | NA -  PS258150 | Spermatogenic failure - 2 carriers  within GEMINI cohort |  | 36572685 |
| USP26 | Xq26.2 | 300309 | XL | 301101 | Spermatogenic failure, X-linked, 6 (MMAF, reduced progressive motility of sperm)  ICSI successful |  | 15562280;15970005; 16888075; 17121659; 17968467;18377898; 18927127; 21147082; 25739334; 27089915; 27726449;  31377750; 32202304; 32410375; 33978233; 35103426; 35857630 |
| USP9Y | Yq11.221 | 400005 | YL | 415000 | Spermatogenic failure, Y-linked,2 | n.r. | 10581029; 11420393; 16893908; 19246359; 25808090 |
| VCF1 (FAM104A) | 17q25.1 | NA | Undetermin ed | NA - PS258150 | NOA - identified by WES from GEMINI cohort |  | 36572685 |
| VCX3A | Xp22.31 | 300533 | Undetermin  ed | NA -  PS258150 | NOA - identified by WGS of men  with NOA |  | 28122887;36017582 |
| WDR19 | 4p14 | 608151 | AR AR AR AR AR | 619867  614378  614377  616307  614367 | ?Spermatogenic failure 72? | Cranioectodermal dysplasia 4  Nephronopthisis 13  Senior-Loken syndrome 8 Short-rib thoracic dysplaisa 5 with or without polydactyly | 32323121 |
| WNK3 | Xp11.22 | 300358 | XLR |  | n.r. | n.r. | 29790874 |
| WWC2 | 4q35.1 | 620110 | Undetermin ed | NA - 258150 | unexplained severe spermatogenic failure | n.r. | 37086090 |
| WWOX | 16q23.1-q23.2 | 605131 | AR AR | 616211  133239  614322 | n.r. | Developmental and epileptic encephalopathy 28 Esophageal squamous cell carcinoma, somatic Spinocerebellar ataxia, autosomal recessive 12 | 33565365 |
| XRCC2 | 7q36.1 | 600375 | AR | ?617247?  ?619146?  619145 | ?Premature ovarian failure 17? Spermatogenic failure 50 | ?Fanconi Anemia, complementation group U? | 23630330; 30042186; 30489636 |
| YY2 | Xp22.12 | 300570 | Undetermin ed | NA - PS305700 | Spermatogenic failure - 1 carrier within GEMINI cohort; 1 carrier from MERGE; 1 carrier from NewRad |  | 36572685 |

| Gene symbol | Locus | OMIM | Inheritance | Phenotype  MIM | GU Phenotype(s) | Other associated  phenotypes | Reference(s) |
| --- | --- | --- | --- | --- | --- | --- | --- |
| ZFPM2 | 8q23.1 | 603693 | AD  AD  Undetermin ed | 616067  610187  187500  PS - 258150 | Spermatogenic failure - 3 carriers identified by WGS | 46,XY sex reversal 9 Diaphragmatic hernia 3 Tetralogy of Fallot | 24549039;36017582 |
| ZFX | Xp22.11 | 314980 | XL | NA - PS305700 | Spermatogenic failure |  | 32469048;36017582 |
| ZMYM3 | Xq13.1 | 300061 | XLR  Undetermin ed | 301111  NA - PS305700 | Intellectual developmental disorder, X-linked 112 (hypospadias, cryptorchidism, vesicoureteral reflux, enuresis) Spermatogenic failure - 1 carrier identified by WGS of men with NOA |  | 36017582;28661483 |
| ZMYND10 | 17p13.2 | 614312 | AR | 615444 | Ciliary dyskinesia, primary, 22 | Ciliary dyskinesia, primary, 22, with and without situs inversus, associated  sinopulmonary phenotypes, | 23891469; 23891471; 33635866 |
| ZMYND15 | 17p13.2 | 614312 | AR | ?615842? | ?Spermatogenic failure 14? | n.r. | 20675388; 24431330; 32655042; 33169450; 35017390; 35973810 |
| ZNF610 | 19q13.41 | NA | Undetermin ed | NA - PS258150 | Spermatogenic failure - 2 carriers within GEMINI cohort |  | 36572685 |
| ZPBP | 7p12.2 | 608498 | AR | ?619799? | ?Spermatogenic failure 66? | n.r. | 17664285; 31985809 |

| Gene symbol | Locus | OMIM | Inheritan  ce | Phenotype  MIM | GU (or endocrine) Phenotype(s) | Other associated phenotypes | Reference(s) |
| --- | --- | --- | --- | --- | --- | --- | --- |
| **Hypothalamic-Pituitary Defect** | | | | | | | |
| AMN1 | 12p11.21 | 620564 | AR | NA - PS 147950 | norosmic hypogonadotropic hypogonadism - 1 carrier identified by NGS of patients with nHH/KS |  | 27502037 |
| ANOS1 (KAL1) | Xp22.31 | 300836 | XLR | 308700 | Kallmann syndrome 1 |  | 29211946; 25739677; 22035731; 21168128; 18259106; 17213338; 16500342; 28122887; 28833369; 28780519;  26862482; 26629483; 26278626; 26199944; 26051373; 26031747; 25636053; 25597551; 25328414; 25064402;  24732674; 24232061; 24204987; 23721716; 23533228; 23410897; 23200691; 22724017; 21717404; 21682876;  20530987; 20591981; 19734936; 18463157; 18160472; 17603054; 17322486; 17223984; 16423815; 16042321;  15605412; 15001591; 14689055; 12773801; 12727945; 12050219; 11456260; 11124862; 11044805; 10944855;  10670750; 9787096; 9589672; 9554756; 8989261; 8504298; 8473391; 1518845; 1594017; 1922361; 30098700;  31996231; 31669640; 31781422; 31200363; 30669598; 32400067; 36039580; 35669683; 35432193; 35133534;  32982993; 34348883; 32763379; 31996231; 2687610; 1922361; 1913827; 7545624; 8842728; 8832397; 11297579;  15001591; 18034870; 18160472; 20696889; 21682876; 22927827; 24002956; 24732674; 30921766; 23533228; 30669598 |
| ANOS1 (KAL1) | Xp22.31 | 300836 | XLR | 308700 | Isolated hypogonadotropic hypogonadism 1 |  | 29211946; 25739677; 22035731; 21168128; 18259106; 17213338; 16500342; 28122887; 28833369; 28780519;  26862482; 26629483; 26278626; 26199944; 26051373; 26031747; 25636053; 25597551; 25328414; 25064402;  24732674; 24232061; 24204987; 23721716; 23533228; 23410897; 23200691; 22724017; 21717404; 21682876;  20530987; 20591981; 19734936; 18463157; 18160472; 17603054; 17322486; 17223984; 16423815; 16042321;  15605412; 15001591; 14689055; 12773801; 12727945; 12050219; 11456260; 11124862; 11044805; 10944855;  10670750; 9787096; 9589672; 9554756; 8989261; 8504298; 8473391; 1518845; 1594017; 1922361; 30098700;  31996231; 31669640; 31781422; 31200363; 30669598; 32400067; 36039580; 35669683; 35432193; 35133534;  32982993; 34348883; 32763379; 31996231; 2687610; 1922361; 1913827; 7545624; 8842728; 8832397; 11297579;  15001591; 18034870; 18160472; 20696889; 21682876; 22927827; 24002956; 24732674; 30921766; 23533228; 30669598 |
| AXL | 19q13.2 | 109135 | AD | NA - PS308700 | Kallman syndrome per Houston et. al. 2020 |  | 24476074;30098700;31748124 |
| B4GAT1 | 11q13.2 | 605517 | AR  Undeter mined | 615287  NA - PS147950 | Muscular dystrophy-dystroglycanopathy (congenital with brain and eye anomalies), type A, 13  Hypogonadotropic hypogonadism per Houston et.  al. 2020 |  | 30098700;36123965 |
| CASR | 3q13.33-q21.1 | 601199 | AD AD, AR AD  AD AD  Undeter  mined | 612899  239200  601198  601198  145980  NA - PS147950 | Hypogonadotropic hypogonadism per Houston et. al. 2020 | [?epilepsy idiopathic generalized, susceptibility to, 8] Hyperparathyroidism, neonatal Hypocalcemia, autosomal dominant Hypocalcemia, autosomal dominant, with Bartter syndrome  Hypocalciuric hypercalcemia | 30098700;37964948 |
| CCDC141 | 2q31.2 | 616031 | AR, olig | NA - PS616030 | Association pending confirmation - hypogonadotropic hypogonadism with or without anosmia |  | 28324054;27014940;32520725;25192046;34930920;33208564 |
| CCKAR | 4p15.2 | 118444 | AR | NA - PS147950 | Hypogonadotropic hypogonadism per Houston et. al. 2020 |  | 30098700;36123965;28030855 |
| CCKBR | 11p15.4 | 118445 | Undeter mined | NA - PS147950 | Hypogonadotropic hypogonadism with or without anosmia per Houston et. al. 2020 |  | 27502037 |
| CHD7 | 8q12.2 | 608892 | AD AD | 214800  612370 | Kallmann syndrome 5 without CHARGE phenotype | CHARGE syndrome | 16155193;25739677;22035731;28833369;28209183;26199944;26031747;25383892;25472840;25064402;24732674;  23533228;22724017;22399515;21682876;18834967;19021638;30098700;31219235;31200363;31200363;30733481;  30669598;30669598;32573075;37814704;37799300;37368388;37305875;37231428;37108593;36794641;36245975;  35047002;34914268;34563184;33680884;33208564;32982993;32763379;32724172 |
| CHD7 | 8q12.2 | 608892 | AD AD | 214800  612370 | Isolated hypogonadotropic hyponadism 5 without CHARGE phenotype | CHARGE syndrome | 16155193;25739677;22035731;28833369;28209183;26199944;26031747;25383892;25472840;25064402;24732674;  23533228;22724017;22399515;21682876;18834967;19021638;30098700;31219235;31200363;31200363;30733481;  30669598;30669598;32573075;37814704;37799300;37368388;37305875;37231428;37108593;36794641;36245975;  35047002;34914268;34563184;33680884;33208564;32982993;32763379;32724172 |
| CNTN2 | 1q32.1 | 190197 | AR  Undeter mined | 615400  NA - PS147950 | Hypogonadotropic hypogonadism per Houston et. al. 2020 | ?Epilepsy, myoclonic, familial adult, 5? | 30098700 |
| CXCR4 | 2q22.1 | 162643 | AD AD  Undeter  mined | 193670  193670  NA - PS147950 | Hypogonadotropic hypogonadism with or without anosmia per Houston et. al. 2020 | Myelokathexis, isolated WHIM syndrome 1 | 27502037 |
| DCAF17 | 2q31.1 | 612515 | AR | 241080 | Woodhouse-Sakati syndrome |  | 30098700;20507343;33208564 |
| DCAF17 | 2q31.1 | 612515 | AR | NA - PS147950 | Hypogonadotropic hypogonadism per Houston et. al. 2020 |  | 30098700;20507343;33208564 |
| DCC | 18q21.2 | 120470 | AR AD  HH: AD,  olig | 114500  133239  617542  157600  NA - PS147950 | Association pending confirmation - Kallmann syndrome | Colorectal cancer, somatic Esophageal carcinoma, somatic Gaze palsy, familial horizontal, with progressive scoliosis, 2  Mirrow movements 1 and/or agenesis of the corpus callosum | 29202173;30098700;22457493;14750959;33208564 |
| DLX5 | 7q21.3 | 600028 | AR AD  Undeter  mined | 220600  183600 | Hypogonadotropic hypogonadism per Houston et. al. 2020 | ?Split-hand/foot malformation 1 with sensorineural hearing loss?  Split-hand/foot malformation 1 | 30098700 |

| Gene symbol | Locus | OMIM | Inheritan  ce | Phenotype  MIM | GU (or endocrine) Phenotype(s) | Other associated phenotypes | Reference(s) |
| --- | --- | --- | --- | --- | --- | --- | --- |
| DMXL2 | 15q21.2 | 612186 | AD AR AR | 617605  616113  618663 | ?Deafness, autosomal dominant 71?  ?Polyendocrine-polyneuropathy syndrome? Developmental and epileptic encephaloptathy 81 |  | 33208564;25248098;31200363;36700485 |
| DMXL2 | 15q21.2 | 612186 | AR | NA - PS147950 | Kallmann syndrome per Houston et. al. 2020 |  | 33208564;25248098;31200363;36700485 |
| DMXL2 | 15q21.2 | 612186 | AR | NA - PS147950 | Isolated hypogonadotropic hypogonadism per Houston et. al. 2020 |  | 33208564;25248098;31200363;36700485 |
| DUSP6 | 12q21.33 | 602748 | AD, Olig | 615269 | Hypogonadotropic hypogonadism 19 with or without anosmia - Kallmann syndrome per Houston et. al. 2020 |  | 25636053;23643382;30669598;32389901;17164422;37108593 |
| EBF2 | 8p21.2 | 609934 | Undeter mined | NA - PS147950 | Hypogonadotropic hypogonadism with or without anosmia |  | 16423815;30098700 |
| EDNRB | 13q22.3 | 131244 | Undeter mined | NA - PS147950 | Hypogonadotropic hypogonadism | ABCD syndrome  Hirschsprung disease, susceptibility to, 2  Waardenburg syndrome, type 4A | 30098700 |
| EFNA5 | 5q21.3 | 601535 | AD | NA - PS147950 | Hypogonadotropic hypogonadism |  | 30098700 |
| EGF | 4q25 | 131350 | AR  undeterm ined | 611718  NA - PS147950 | ?Hypomagnesemia 4, renal? Hypogonadotropic hypogonadism |  | 30098700;33208564 |
| EGFR | 7p11.2 | 131550 | AR  AD, SMu AD, SMu AD, SMu  undeterm  ined | 616069  211980  211980  211980  NA - PS147950 | Hypogonadotropic hypogonadism | ?Inflammatory skin and bowel disease, neonatal, 2?  {Nonsmall cell lung cancer, susceptibility to}  Adenocarcinoma of lung, response to tyrosine kinase inhibitor in  Nonsmall cell lung cancer, response to tyrosine kinase iinhibitor in | 30098700 |
| EMX2 | 10q26.11 | 600035 | AD | 269160  NA - PS147950 | Hypogonadotropic hypogonadism | Schizencephaly | 10560999 |
| EPHA5 | 4q13.1-q13.2 | 600004 | Undeter mined | NA - PS147950 | Hypogonadotropic hypogonadism |  | 30098700 |
| ERBB4 | 2q34 | 600543 | AD AD | 615515  NA - PS157950 | Isolated hypogonadotropic hypogonadism | Amyotrophic lateral sclerosis 19 | 30098700;36123965 |
| FEZ1 | 11q24.2 | 604825 |  |  | Hypogonadotropic hypogonadism |  | 30098700 |
| FEZF1 | 7q31.32 | 613301 | AR, Olig | 616030 | Hypogonadotropic hypogonadism 22, with or without anosmia - Kallmann syndrome per Houston et. al. 2020 |  | 16540508;19479999;25636053;25192046;30669598;32400067;28324054 |
| FGF17 | 8p21.3 | 603725 | AD, Olig | 615270 | Kallmann syndrome 20 |  | 23643382;16384934;25636053;25192046;30669598;37799300;37108593;31748124;31200363 |
| FGF17 | 8p21.3 | 603725 | AD, Olig | 615270 | Isolated hypogonadotropic hypogonadism 20 |  | 23643382;16384934;25636053;25192046;30669598;37799300;37108593;31748124;31200363 |
| FGF8 | 10q24.32 | 600483 | Olig | 612702 | Hypogonadotropic hypogonadism 6 with or without anosmia  Kallman Syndrome |  | 34276780; 31748124; 31200363; 30669598; 26199944; 26031747; 25739677; 25636053; 25064402; 24732674;  23533228; 23200691; 22724017; 21832120; 22035731; 21682876; 20463092; 20553372; 18596921; 17264867; 17235395 |
| FGF8 | 10q24.32 | 600483 | Olig | 612702 | Hypogonadotropic hypogonadism 6 with or without anosmia  Isolated (norosmic) per Houston Review |  | 34276780; 31748124; 31200363; 30669598; 26199944; 26031747; 25739677; 25636053; 25064402; 24732674;  23533228; 23200691; 22724017; 21832120; 22035731; 21682876; 20463092; 20553372; 18596921; 17264867; 17235395 |
| FGFR1 | 8p11.23 | 136350 | AD  AD, AR,  dig, olig AD  AD  AD AD | 613001  615465  147950  123150  166250  101600  190440 | Kallmann syndrome 2 | Encephalocraniocutaneous lipomatosis, somatic mosaic Hartsfield syndrome  Jackson-Weiss syndrome Osteoglophonic dysplasia Pfeiffer syndrome Trigonocephaly 1 | 25739677;22035731;17200176;16764984;15845591;15613419;29658329;29228280;28833369;28411082;28209183;  28195315;28087897;28008864;26277103;26199944;26051373;26031747;25636053;25394172;25383892;25064402;  24732674;24522099;24204987;23643382;23533228;23200691;23154428;22724017;21682876;21664240;21292259;  20591981;20536592;19820032;18723471;18463157;18160472;17235395;17154279;16882753;16757108;16606836;  16418210;16061567;15605412;15001591;12627230;30098700;31996231;31996231;31910188;31748124;31748124;  31781422;31200363;31200363;30669598;30669598;32485746;32485746;37814704;37805574;36859276;36407308;  36384729;36138264;35669683;35668409;35457241;35133534;34348883;33983622;33775534;33819414;33680884 |
| FGFR1 | 8p11.23 | 136350 | AD  AD, AR,  dig, olig AD  AD  AD AD | 613001  615465  147950  123150  166250  101600  190440 | Isolated hypogonadotropic hypogonadism 2 | Encephalocraniocutaneous lipomatosis, somatic mosaic Hartsfield syndrome  Jackson-Weiss syndrome Osteoglophonic dysplasia Pfeiffer syndrome Trigonocephaly 1 | 25739677;22035731;17200176;16764984;15845591;15613419;29658329;29228280;28833369;28411082;28209183;  28195315;28087897;28008864;26277103;26199944;26051373;26031747;25636053;25394172;25383892;25064402;  24732674;24522099;24204987;23643382;23533228;23200691;23154428;22724017;21682876;21664240;21292259;  20591981;20536592;19820032;18723471;18463157;18160472;17235395;17154279;16882753;16757108;16606836;  16418210;16061567;15605412;15001591;12627230;30098700;31996231;31996231;31910188;31748124;31748124;  31781422;31200363;31200363;30669598;30669598;32485746;32485746;37814704;37805574;36859276;36407308;  36384729;36138264;35669683;35668409;35457241;35133534;34348883;33983622;33775534;33819414;33680884 |

| Gene symbol | Locus | OMIM | Inheritan  ce | Phenotype  MIM | GU (or endocrine) Phenotype(s) | Other associated phenotypes | Reference(s) |
| --- | --- | --- | --- | --- | --- | --- | --- |
| FGFR1 | 8p11.23 | 136350 | AD  AD, AR,  dig, olig AD  AD AD AD | 613001  615465  147950  123150  166250  101600  190440  NA | Late onset hypogonadotropic hypogonadism - per Houston et. al. 2020 | Encephalocraniocutaneous lipomatosis, somatic mosaic Hartsfield syndrome  Jackson-Weiss syndrome Osteoglophonic dysplasia Pfeiffer syndrome Trigonocephaly 1 | 25739677;22035731;17200176;16764984;15845591;15613419;29658329;29228280;28833369;28411082;28209183;  28195315;28087897;28008864;26277103;26199944;26051373;26031747;25636053;25394172;25383892;25064402;  24732674;24522099;24204987;23643382;23533228;23200691;23154428;22724017;21682876;21664240;21292259;  20591981;20536592;19820032;18723471;18463157;18160472;17235395;17154279;16882753;16757108;16606836;  16418210;16061567;15605412;15001591;12627230;30098700;31996231;31996231;31910188;31748124;31748124;  31781422;31200363;31200363;30669598;30669598;32485746;32485746;37814704;37805574;36859276;36407308;  36384729;36138264;35669683;35668409;35457241;35133534;34348883;33983622;33775534;33819414;33680884 |
| FLRT3 | 20p12.1 | 604808 | Olig | 615271 | Kallmann syndrome 21 |  | 25636053;23643382;30669598;14688794;15485775;37108593;36700485;31200363 |
| FLRT3 | 20p12.1 | 604808 | Olig | 615271 | Isolated hypogonadotropic hypogonadism 21 |  | 25636053;23643382;30669598;14688794;15485775;37108593;36700485;31200363 |
| FSHB | 11p14.1 | 136530 | AR | 229070 | Hypogonadotropic hypogonadism 24 without anosmia |  | 15846219;12568861;12161499;9806482;25064402;9624193;31439307;32242295 |
| GAP43 | 3q13.31 | 162060 | Undeter mined | NA - PS147950 | Kallmann syndrome per Houston et. al. 2020 |  | 27502037 |
| GATA2 | 3q21.3 | 137295 | AD AD  AD, SMu | 614038  614172  601626  614286 |  | Emberger syndrome Immunodeficiency 21  {Leukemia, acute myeloid, susceptibility to}  {Myelodysplastic syndrome,  susceptibility to} | 11807026; 16543408; 21571865; 23211524 |
| GH1 | 17q23.3 | 139250 | AR  AD AR | 262400  612781  173100  262650 | Isolated growth hormone deficiency, type 1A Isolated growth hormone deficiency, type 1B Isolated growth hormone deficiency type II | Kowarski syndrome | 17132747;30098700 |
| GHR | 5p13.1-  p12 | 600946 | AD, AR AD  AD AR  Undeter  mined | NA - PS147950 | Hypogonadotropic hypogonadism | {Hypercholesterolemia, familial, modifier of}  Growth hormone insensitivity, partial Increased responsiveness to growth hormone  Laron dwarfism | 30098700 |
| GLCE | 15q23 | 612134 | Undeter mined | NA - PS147950 | Hypogonadotropic hypogonadism |  | none found... |
| GLI2 | 2q14.2 | 165230 | AD AD | 615849  610829 | Culler-Jones syndrome Holoprosencephaly 9 |  | 22106008 |
| GLI3 | 7p14.1 | 165240 | AD AD AD AD | 175700  146510  174200  174700 | Pallister-Hall syndrome (hypothalamic hamartoma, pituitary aplasia or dysplasia, panhypopituitarism, micropenis, cryptorchidism, testicular hypoplasia, renal dysplasia, renal ectopia) | Greig cephalopolysynactyly syndrome  Polydactyly, postaxial, type a1 Polydactyly, preaxial IV | 27502037; 31219235; 9054938; 10441570; 12545275; 10945658 |
| GLI3 | 7p14.1 | 165240 | AD | NA - PS 308700 | Kallman syndrome per Houston et. al 2020 (literature suggests KS or nCHH) |  | 27502037; 31219235; 9054938; 10441570; 12545275; 10945658 |
| GNRH1 | 8p21.2 | 152760 | AR olig | 614841 | Hypogonadotropic hypogonadism 12 with or without anosmia (isolated per Houston et. al. 2020) |  | 27544332;25739677;23936060;22035731;15846219;12788881;26199944;26031747;25636053;25064402;22766261;  20591981;19567835;19535795;18463157;17179725;14689055;30098700;32134721;30669598;26595427;37798680;  37372384;35805171;35133534;34923491;33819414;32813678 |
| GNRH2 | 20p13 | 602352 | Undeter mined | NA - PS147950 | Kallmann syndrome - per Houston et. al. 2020 |  | 30669598 |
| GNRH2 | 20p13 | 602352 | Undeter mined | NA - PS147950 | Isolated hypogonadotropic hypogonadism - per Houston et. al. 2020 |  | 30669598 |
| GNRHR | 4q13.2 | 138850 | AR, olig | 146110 | Hypogonadotropic hypogonadism 7 without anosmia (isolated per Houston et. al. 2020) (phenotypic variability - partial or complete hypogonadotropic hypogonadism) |  | 27544332;25739677;22035731;21717411;16968799;16500342;16213849;15846219;12788881;12477532;11397842;  29182666;28611058;26199944;26031747;25636053;25531638;25064402;24732674;24204987;22788855;23295295;  22766261;22724017;21664240;21292259;18463157;17235395;17223984;17074994;16868131;16423815;16322390;  14689055;12568864;11397871;11318785;11297587;10698591;10084584;10022417;9425890;9418701;9371856;1756212;  2546961;29777911;30415482;30098700;31200363;31200363;30947225;30669598;30575316;37338467;36700485;  36407308;36371229;36138264;35798295;35133534;33968656;33680884;33592857;32763379;32134721 |
| HESX1 | 3p14.3 | 601802 | AD, AR AD, AR AD, AR AD | 182230  182230  182230  NA - PS147950 | Growth hormone deficiency with pituitary anomalies  Pituitary hormone deficiency, combined, 5 Septooptic dysplasia  Kallmann syndrome - per Houston et. al. 2020 |  | 23465708;15846219;25064402;30888394;30669598;36700485;35805171;33270637;33098107 |

| Gene symbol | Locus | OMIM | Inheritan  ce | Phenotype  MIM | GU (or endocrine) Phenotype(s) | Other associated phenotypes | Reference(s) |
| --- | --- | --- | --- | --- | --- | --- | --- |
| HFE | 6q22.2 | 613609 | AR AD  AD, AR  AD | 235200  614193  104300  612635  176100  176200 | hemochromatosis, type 1, associated with impotence, testicular atrophy, azoospermia, hypogonadotripic hypogonadism | Hemochromatosis  [Transferrin serum level QTL2]  {Alzheimer disease, susceptibility to}  {Microvascular complications of diabetes 7}  {Porphyria cutanea tarda, susceptibility to}  {Porphyria variegata, susceptibility to} | 18846434 |
| HGF | 7q21.11 | 142409 | AR | 608265  NA - PS147950 | Hypogonadotropic hypogonadism - per Houston et. al. 2020 | Deafness, autosomal recessive 39 | 30098700 |
| HS6ST1 | 2q14.3 | 604846 | AD,Olig | 614880 | Kallmann syndrome15 |  | 25739677;21700882;26031747;25064402;23533228;29931354;31377750;30669598;30669598;35805171;35669683;  33819414;30467832 |
| HS6ST1 | 2q14.3 | 604846 | AD,Olig | 614880 | Isolated hypogonadotropic hypogonadism15 |  | 25739677;21700882;26031747;25064402;23533228;29931354;31377750;30669598;30669598;35805171;35669683;  33819414;30467832 |
| IGF1 | 12q23.2 | 147440 | AR  Undeter mined | 147440  NA - PS147950 | Hypogonadotropic hypogonadism - per Houston et. al. 2020 |  | 30098700 |
| IGFALS | 16p13.3 | 601489 | AR AR | 615961  NA - PS147950 | Kallmann syndrome - per Houston et. al. 2020 | Acid-labile subunit, deficiency of | 31200363 |
| IGSF10 | 3q25.1 | 617351 | AD | N/A (PS147950  ) | Association pending confirmation - Hypogonadotropic hypogonadism 2 with or without anosmia; delayed puberty |  | 27137492;31200363;35133534;35047120;33208564;32612575;31726455 |
| IL17RD | 3p14.3 | 606807 | AD, AR, DD | 615267 | Kallmann syndrome 18 |  | 28833369;25636053;23643382;30098700;31377750;31200363;31200363;30669598;30669598;32389901;37108593;  36138264;36123965;35805171;35669683;35133534;33270637 |
| IL17RD | 3p14.3 | 606807 | AD, AR, DD | 615267 | Isolated hypogonadotropic hypogonadism 18 |  | 28833369;25636053;23643382;30098700;31377750;31200363;31200363;30669598;30669598;32389901;37108593;  36138264;36123965;35805171;35669683;35133534;33270637 |
| JAG1 | 20p12.2 | 601920 | AD AD AD AD AD | 617992  118450  619574  187500  NA -PS 147950 | Isolated hypogonadotropic hypogonadism - per Houston et. al. 2020 | ?Deafness, congenital heart defects, and posterior embryotoxon?  Alagille syndrome 1  Charcot-Marie-Tooth disease, axonal, type 2HH  Tetralogy of Fallot | 27502037;36729644 |
| KISS1 | 1q32.1 | 603286 | AR | 614842 | ?Hypogonadotropic hypogonadism 13 with or without anosmia? |  | 27544332;25739677;26199944;26031747;25783047;25636053;25064402;22724017;20237166;17179725 |
| KISS1R | 19p13.3 | 604161 | AR | 176400  614837 | ?precocious puberty, cetnral, 1? Kallmann syndrome 8 |  | 27544332;25739677;22035731;29452377;29264451;28833369;27094476;26199944;26031747;25636053;25262569;  25064402;24732674;24522099;23643382;23349759;22766261;22724017;22619348;21193544;20371656;18463157;  17164310;17179725;17074994;16322390;12944565;14573733;30098700;31885997;31821609;31377750;31073722;  30669598;30669598;15598687;31821609;37814704;35735778;35133534;33819414;33270637 |
| KISS1R | 19p13.3 | 604161 | AR | 614837 | Isolated hypogonadotropic hypogonadism 8 |  | 27544332;25739677;22035731;29452377;29264451;28833369;27094476;26199944;26031747;25636053;25262569;  25064402;24732674;24522099;23643382;23349759;22766261;22724017;22619348;21193544;20371656;18463157;  17164310;17179725;17074994;16322390;12944565;14573733;30098700;31885997;31821609;31377750;31073722;  30669598;30669598;15598687;31821609;37814704;35735778;35133534;33819414;33270637 |
| KLB | 4p14 | 611135 | AR | NA - PS147950 | Hypogonadotropic hypogonadism |  | 28754744;35047120 |
| LEP | 7q32.1 | 164160 | AR | 614962 | Obesity, morbid, due to leptin deficiency (with hypothalamic hypogonadism) |  | 29101506; 12087499; 15342807; 7984236; 9537324; 10486419; 19179437; 21296922; 23505551; 25084037; 25064402;  26031747; 26586796; 28209183; 29905528 |
| LEPR | 1p31.3 | 601007 | AR | 614963 | Obesity, morbid, due to leptin receptor deficiency (hypogonadotropic hypogonadism) |  | 7984236; 9537324; 17229951; 16503925; 25064402; 26031747; 30098700; 30778850 |
| LHB | 19q13.33 | 152780 | AR | 228300 | Hypogonadotropic hypogonadism 23 without anosmia |  | 17761593; 19126631; 22723313 |
| LHX3 | 9q34.3 | 600577 | AR | 221750 | Pituitary hormone deficiency, combined, 3 (Kallmann syndrome per Houston et. al. 2020) |  | 25064402;22286346;21249393;17327381;16394081;35805171;33098107 |
| LHX4 | 1q25.2 | 602146 | AD | 262700 | Pituitary hormone deficiency, combined, 4 |  | 25064402;31377750;30888394;35805171;33098107 |
| LIF | 22q12.2 | 159540 | Undeter mined | NA - PS147950 | Hypogonadotropic hypogonadism - per Houston et. al. 2020 |  | 30098700 |
| MASTL | 10q12.1 | 608221 | AR | NA - PS147950 | Hypogonadotropic hypogonadism - per Houston et. al. 2020 |  | 27502037 |
| MC4R | 18q21.32 | 155541 | AD, AR AD, AR AR | 618406  618406  NA | Obesity with hypogonadotropic hypogonadism - per Houston et. al. 2020 |  | 21921657 |

| Gene symbol | Locus | OMIM | Inheritan  ce | Phenotype  MIM | GU (or endocrine) Phenotype(s) | Other associated phenotypes | Reference(s) |
| --- | --- | --- | --- | --- | --- | --- | --- |
| MET | 7q31.2 | 164860 | AD AR AD  AD | 620019  616705  607278  114550  605074  NA - PS147950 | Hypogonadotropic hypogonadism - per Houston et. al. 2020 | ?Arthrogryposis, distal, type 11?  ?Deafness, autosomal recessive 97?  {Osteofibrous dysplasia, susceptibility to}  Hepatocellular carcinoma, childhood type, somatic  Renal cell carcinoma, papillary, 1,  familial and somatic | 30098700 |
| NDNF | 4q27 | 616506 | AD | 618841 | Hypogonadotropic hypogonadism 25 with anosmia |  | 31883645;36454653;36245975;33531459 |
| NOTCH1 | 9p34.3 | 190198 | AD AD  Undeter  mined | 616028  109730  NA - PS147950 | Isolated Hypogonadotropic hypogonadism - per Houston et. al. 2020 | Adams-Oliver syndrome 5 Aortic valve disease 1 | 36572685;27502037;33208564 |
| NPY1R | 4q32.2 | 162641 | Undeter mined | NA - PS147950 | Hypogonadotropic hypogonadism - per Houston et. al. 2020 |  | 17140570 |
| NR0B1 (DAX1) | Xp21.2 | 300473 | XLR | 300200 | Adrenal hypoplasia, congenital (with HH) |  | 28741070;27648561;26260363;26207377;25529318;23384712;21227944;16645015;16556678;16275267;11788621;  10675358;29176027;28924487;28284037;28075027;27711951;27035099;26537215;26030781;25993682;25968435;  25064402;25003377;24197767;24232823;24140641;23585174;23295288;23018754;22761912;22562240;21925982;  21632081;21270512;20975255;21340153;20573681;19508677;19773398;19672728;19129717;18607630;18604556;  18941128;18380948;18202527;18056774;17054473;16553032;16355812;15884018;15860922;15841486;14689055;  14689056;12773801;12629128;11443184;11113848;10931108;10599709;10599708;10522996;10361383;10341858;  10022408;9508067;9415399;9195207;9003500;8855822;8770879;8675564;8636263;8844218;7990958;28483799;  30179867;30129976;32129306;32028936;31917682;31700544;31642359;31377750;31280422;31219797;30620004;  32166680;35849255;35432221;32860660;37237297;37118935;36160878;37906859;35848959;35784540;35417110;  35230670;34938333;34373561;34130666;33381670;32460754;32129306;32028936; 31280422;31219797;31164167 |
| NR0B1 (DAX1) | Xp21.2 | 300473 | XL | NA - PS147950 | Late-onset adrenal failure or isolated hypogonadotropic hypogonadism - per Houston et. al. 2020 |  | 28741070;27648561;26260363;26207377;25529318;23384712;21227944;16645015;16556678;16275267;11788621;  10675358;29176027;28924487;28284037;28075027;27711951;27035099;26537215;26030781;25993682;25968435;  25064402;25003377;24197767;24232823;24140641;23585174;23295288;23018754;22761912;22562240;21925982;  21632081;21270512;20975255;21340153;20573681;19508677;19773398;19672728;19129717;18607630;18604556;  18941128;18380948;18202527;18056774;17054473;16553032;16355812;15884018;15860922;15841486;14689055;  14689056;12773801;12629128;11443184;11113848;10931108;10599709;10599708;10522996;10361383;10341858;  10022408;9508067;9415399;9195207;9003500;8855822;8770879;8675564;8636263;8844218;7990958;28483799;  30179867;30129976;32129306;32028936;31917682;31700544;31642359;31377750;31280422;31219797;30620004;  32166680;35849255;35432221;32860660;37237297;37118935;36160878;37906859;35848959;35784540;35417110;  35230670;34938333;34373561;34130666;33381670;32460754;32129306;32028936; 31280422;31219797;31164167 |
| NRP1 | 10p11.22 | 602069 | AD | NA - PS147950 | Hypogonadotropic hypogonadism - per Houston et. al. 2020 |  | 30098700;34636164 |
| NRP2 | 2q33.3 | 602070 | Undeter mined | NA - PS147950 | Hypogonadotropic hypogonadism - per Houston et. al. 2020 |  | 30098700;34636164;32171629;27502037 |
| NSMF (NELF) | 9q34.3 | 608137 | AR, olig | 614838 | Kallmann syndrome 9 |  | 25739677;26199944;26031747;25636053;25064402;23533228;20591981;18463157;17235395;16423815;15362570;  22035731;21300340;15846219;30098700;31200363;30669598;30669598;34348883;33270637;35316923;31220265 |
| NSMF (NELF) | 9q34.3 | 608137 | AR, olig | 614838 | Isolated hypogonadotropic hypogonadism 9 |  | 25739677;26199944;26031747;25636053;25064402;23533228;20591981;18463157;17235395;16423815;15362570;  22035731;21300340;15846219;30098700;31200363;30669598;30669598;34348883;33270637;35316923;31220265 |
| NTN1 | 17p13.1 | 601614 | AD, olig | NA - PS147950 | Per Bouilly et. al. 2018, two individuals with CHH had variants in both DCC and NTN1 |  | 29202173 |
| OTUD4 | 4q31.21 | 611744 | AR, olig | NA: PS212840 | Association pending confirmation- Gordon Homes syndrome (Cerebellar ataxia and hypogonadotropic hypogonadism) |  | 11932290; 23656588; 24113144 |
| OTUD4 | 4q31.21 | 611744 | Undeter mined | NA - PS147950 | Kallmann syndrome - per Houston et. al. 2020 |  | 31748124 |
| OTX2 | 14q22.3 | 600037 | AD AD AD | 610125  613986  610125 | Pituitary hormone deficiency, combined, 6 | Micropthalmia, syndromic 5  Retinal dystrophy, early-onset, with or without pituitary dysfunction | 25064402; 15201224; 18781617; 19956411; 20486942; 22715480; 23990694; 21436260; 27299576 |
| PALM2AKAP2 | 9q31.3 | 604582 | Undeter mined | NA - PS 147950 | Kallmann syndrome; hypogonadotropic hypogonadism, delayed puberty, cryptorchidism, small testes per Houston et. al. 2021 | unknown | 17273791;33909591 |
| PAX6 | 11p13 | 607108 | AD AD AD AD AD AD AD AD AD AD | 120430  120200  120430  106210  604229  106210  136520  148190  165550  NA - PS147950 | Hypogonadotropic hypogonadism - per Houston et. al. 2020 | ?coloboma of optic nerve?  ?coloboma, ocular?  ?morning glory disc anomaly? Aniridia  Anterior segment dysgenesis 5, multiple subtypes  Cataract with late-onset corneal dystrophy  Foveal hypoplasia 1 Keratitis  Optic nerve hypoplasia | 30098700 |

| Gene symbol | Locus | OMIM | Inheritan  ce | Phenotype  MIM | GU (or endocrine) Phenotype(s) | Other associated phenotypes | Reference(s) |
| --- | --- | --- | --- | --- | --- | --- | --- |
| PCSK1 | 5q15 | 162150 | AR | 600955  612362 | Endocrinopathy due to proprotein convertase 1/3 deficiency  {Obesity, susceptibility to, BMIQ12} |  | 9207799; 17595246 |
| PDE3A | 12p12.2 | 123805 | AD AD | 112410  NA - PS147950 | Kallmann syndrome - per Houston et. al. 2020 | Hypertension and brachydactyly syndrome | 27502037;33208564 |
| PIN1 | 19p13.2 | 601052 | Undeter mined | NA - PS147950 | Hypogonadotropic hypogonadism - per Houston et. al. 2020 |  | 23096908 (withdrawn?) |
| PITX2 | 4q25 | 601542 | AD AD AD | 137600  180500  180550 | Axenfeld-Rieger syndrome, type1 (Cangiano et. al. suggest phenotype could include CHH) | Anterior segment dysgenesis 4 Ring dermoid of cornea | 10852367; 11807026; 28972279 |
| PLEKHA5 | 12p12.3 | 607770 | AD | NA -  PS147950 | Kallmann syndrome - per Houston et. al. 2020 |  | 27502037 |
| PLXNA1 | 3q21.3 | 601055 | AR, olig | 619955 | Association pending confirmation - hypogonadotropic hypogonadism with or without anosmia | Dworschak-Punetha neurodevelopmental syndrome | 30467832;30098700;28334861;24065959;31690636;34636164;32171629 |
| PLXNA3 | Xq28 | 300022 | Undeter mined | NA - PS308700 | possibly involved in pathogenesis of idiopathic hypogonadotropic hypogonadismper Kotan et. al. 2021 |  | 33495532;36110220 |
| PLXNB1 | 3p21.31 | 601053 | Undeter mined | NA - PS147950 | Hypogonadotropic hypogonadism - per Houston et. al. 2020 |  | 30098700;35170806 |
| PNPLA6 | 19p13.2 | 603197 | AR AR AR AR | 245800  215470  275400  612020 | ?Laurence-Moon syndrome? Boucher-Neuhauser syndrome Oliver-McFarlane syndrome | Spastic paraplegia 39, autosomal recessive | 24355708; 25033069 |
| POLR3A | 10q22.3 | 614258 | AR AR | 607694  264090 | Leukodystrophy, hypomyelinating, 7, with or without oligodontia and/or hypogonadotropic hypogonadism  Wiedemann-Rautenstrauch syndrome |  | 21855841; 23355746 |
| POLR3B | 12q23.3 | 614366 | AD AR | 619742  614381 | Charcot-Marie-Tooth disease, demyelinating, type 1I  Leukodystrophy, hypomyelinating, 8, with or  without oligodontia and/or hypogonadotropic hypogonadism |  | 22036172; 23355746 |
| POU1F1 | 3p11.2 | 173110 | AD, AR | 613038 | Pituitary hormone deficiency, combined or isolated, 1 |  | 25064402;30888394, 37948564, 37165954, 34815942, 34270938, 33886498, 33742319 |
| PROK2 | 3p13 | 607002 | AR, AD,  olig | 610628 | Hypogonadotropic hypogonadism 4 with or without anosmia (Kallmann syndrome per Houston et. al. 2020) |  | 25739677;22035731;28209183;26199944;26031747;25636053;25531638;25064402;24732674;23533228;23386640;  23200691;22724017;21682876;21664240;20591981;20022991;18723471;18682503;18559922;18160472;17959774;  17054399;30098700;31781422;31200363;30669598;37108593;36700485;36138264;35133534;35366854;33819414;  32400067 |
| PROKR2 | 20p12.3 | 607123 | AR, AD,  olig | 244200 | Hypogonadotropic hypogonadism 3 with or without anosmia (Kallmann syndrome per Houston et. al. 2020) |  | 25739677;22773735;22035731;18596028;28858133;28833369;28611058;28209183;26199944;26031747;25636053;  25064402;24732674;24204987;24031091;23533228;23386640;23200691;22724017;21682876;21664240;20591981;  20022991;18826963;18723471;18682503;18559922;18285834;18160472;17054399;24276467;30098700;31748124;  31781422;31377750;31219235;31200363;30669598;30669598;37814704;37799300;37338295;36317218;36694982;  36245975;36138264;36123965;36110220;35922219;35236788;35133534;35090434;34348883;33680884;33208564;  35316923;32763379;32400067 |
| PROP1 | 5q35.3 | 601538 | AR | 262600 | Pituitary hormone deficiency, combined, 2 |  | 11283314; 16678101; 15963055; 19128366 |
| RNF216 | 7p22.1 | 609948 | AR, olig | 212840 | Gordon Homes syndrome (Cerebellar ataxia and hypogonadotropic hypogonadism) |  | 11932290; 23656588; 24113144 |
| SEMA3A | 7p12.1 | 603961 | AD, olig | 614897 | Hypogonadotropic hypogonadism 16 with or without anosmia |  | 28833369;26199944;26031747;25985275;25636053;25064402;24522099;22927827;22416012;30098700;32060892;  32060892;31748124;31200363;30669598;36245975;36138264;34721574;35316923;32612575 |
| SEMA3E | 7q21.11 | 608166 | Olig | ?147950? | Association pending confirmation - hypogonadotropic hypogonadism with anosmia |  | 30669598;37814704;33270637;30467832 |
| SEMA4D | 9q22.2 | 601866 | Olig? | NA - PS147950 | Hypogonadotropic hypogonadism - per Houston et. al. 2020 |  | 30098700;37013058;36123965 |
| SEMA7A | 15q24.1 | 607961 | AR  Olig? | 619874  614745  NA-PS147950 | Kallmann syndrome - per Houston et. al. 2020 | ?cholestasis, progressive familial intrahepatic, 11?  [Blood group, John-Milton-Hagen  system] | 24522099;30098700;31200363;31200363;30669598;34539727;35316923 |
| SEMA7A | 15q24.1 | 607961 | AR  Olig? | 619874  614745  NA-PS147950 | Isolated hypogonadotropic hypogonadism - per Houston et. al. 2020 | ?cholestasis, progressive familial intrahepatic, 11?  [Blood group, John-Milton-Hagen  system] | 24522099;30098700;31200363;31200363;30669598;34539727;35316923 |
| SLIT2 | 4p15.31 | 603746 | Olig? | NA - PS147950 | Isolated hypogonadotropic hypogonadism - per Houston et. al. 2020 |  | 35797970;33270637;33208564; |
| SMCHD1 | 18p11.32 | 614982 | AD DD | 603457  158901 | Bosma arhinia microphthalmia syndrome | Fascioscapulohumeral muscular dystrophy 2, digenic | 18425126; 22252456; 28067909 |

| Gene symbol | Locus | OMIM | Inheritan  ce | Phenotype  MIM | GU (or endocrine) Phenotype(s) | Other associated phenotypes | Reference(s) |
| --- | --- | --- | --- | --- | --- | --- | --- |
| SOX10 | 22q13.1 | 602229 | AD AD AD AD | 609136  611584  613266  NA - PS147950 | Kallmann syndrome - per Houston et. al. 2020 | PCWH syndrome  Waardenburg syndrome, type 2E, with or without neurologic involvement Waardenburg syndrome, type 4C (olfactory bulb agenesis in some patients) | 25529318;29678855;26228106;24769923;23643381;31377750;30914325;30669598;32400067;37814704;37799300;  36927561;35133534;34766489;34095692;33913437;33597923;33442024;32908489;32763379 |
| SOX10 | 22q13.1 | 602229 | AD AD AD | 609136  611584  613266  NA - PS147950 | Isolated hypogonadotropic hypogonadism - per Houston et. al. 2020 | PCWH syndrome  Waardenburg syndrome, type 2E, with or without neurologic involvement Waardenburg syndrome, type 4C (olfactory bulb agenesis in some patients) | 25529318;29678855;26228106;24769923;23643381;31377750;30914325;30669598;32400067;37814704;37799300;  36927561;35133534;34766489;34095692;33913437;33597923;33442024;32908489;32763379 |
| SOX2 | 3q26.33 | 184429 | AD | 206900 | Microphthalmia, syndromic 3 (with hypogonadotropic hypogonadism) |  | 21326281;28659543;25064402;24457197;24211324;16932809;36602867 |
| SOX2 | 3q26.33 | 184429 | AD | NA - PS147950 | Isolated hypogonadotropic hypogonadism (normosmic) |  | 21326281;28659543;25064402;24457197;24211324;16932809;36602867 |
| SOX3 | Xq27.1 | 313430 | XL XL XL | 300123  312000  307700 | Panhypopituitarism, X-linked | Intellectual developmental disorder, X-linked, with isolated growth hormone deficiency  Hypoparathyroidism, X-Linked | 26260363;15292361;25781358;25064402;22678921;21183788;27260338;29582157;36416214;36189645;36064700;  35164824;34178900;31523625;35295983;35133534;30216942 |
| SOX3 | Xq27.1 | 313430 | XL | NA - PS147950 | Isolated Hypogonadotropic hypogonadism |  | 26260363;15292361;25781358;25064402;22678921;21183788;27260338;29582157;36416214;36189645;36064700;  35164824;34178900;31523625;35295983;35133534;30216942 |
| SPRY4 | 5q31.3 | 607984 | Olig | 615266 | Kallmann syndrome 17 |  | 28833369;25636053;23643382;30669598;30669598;32389901;37394858;35316923;31781046;31200363;31726455 |
| SPRY4 | 5q31.3 | 607984 | Olig | 615266 | Isolated hypogonadotropic hypogonadism 17 |  | 28833369;25636053;23643382;30669598;30669598;32389901;37394858;35316923;31781046;31200363;31726455 |
| SRA1 | 5q31.3 | 603819 | AR | NA - PS147950 | Isolated hypogonadotropic hypogonadism - per Houston et. al. 2020 |  | 27086651;35805171;32982993;35316923;30467832 |
| STS | Xp22.31 | 300747 | XLR XL | NA - PS308700 | Hypogonadotropic hypogonadism - per Houston et. al. 2020 | Ichthyosis, X-linked | 25597551;34987993;30352392;28122887;27264819 |
| STUB1 | 16p13.3 | 607207 | AD AR | 618093  615768 | Spinocerebellar ataxia 48  Spinocerebellar ataxia, autosomal recessive 16 |  | 24113144 |
| TAC3 | 12q13.3 | 162330 | AR | 614839 | Hypogonadotropic hypogonadism 10 with or without anosmia (Kallmann syndrome per Houston et. al. 2020) |  | 27544332;25739677;22035731;26199944;26031747;25636053;25064402;24732674;23329188;22766261;22724017;  22031817;21664240;21292259;20591981;20194706;35133534 |
| TACR3 | 4q24 | 162332 | AR, olig | 614840 | Hypogonadotropic hypogonadism 11 with or without anosmia (Kallmann syndrome per Houston et. al. 2020) |  | 27544332;25739677;22035731;26199944;26031747;25636053;25064402;24732674;23329188;22766261;22724017;  22031817;21664240;21292259;20591981;20194706;19755480;19079066;30390321;30098700;31200363;30669598;  35133534;33363893;30269813; 30216942 |
| TBX3 | 12q24.21 | 601621 | AD | 181450 | Ulnar-mammary syndrome |  | 12116211; 30550377; 36383654 |
| TUBB3 | 16q24.3 | 602661 | AD AD | 614039  600638 | Per Chew et al. 2013; Balasubramanian et a. 2015; Nakamura et al. 2018; a single missense mutation (p.E410K) of TUBB3 was also associated with KS and severe peripheral neuropathy, having with an AD inheritance pattern with strong genotype–phenotype correlation | Cortical dysplasia, complex, with other brain malformations 1  Fibrosis of extraocular muscles,  congenital, 3A | 26639658; 27428177; 29382549; 23378218; 25559402; 29289389 |
| WDR11 | 10q26.12 | 606417 | AD, olig AR | 614858  620237 | Isolated hypogonadotropic hypogonadism 14 | Intellectual developmental disorder, autosomal recessive 78 | 22035731;20887964;29263200;28209183;26199944;26031747;25636053;25064402;22724017;21682876;30098700;  31200363;30669598;37799300;36130823;35722485;35432193;34741523;33270637;32982993 |
| WDR11 | 10q26.12 | 606417 | AD, olig AR | 614858  620237 | Kallmann syndrome 14 | Intellectual developmental disorder, autosomal recessive 78 | 22035731;20887964;29263200;28209183;26199944;26031747;25636053;25064402;22724017;21682876;30098700;  31200363;30669598;37799300;36130823;35722485;35432193;34741523;33270637;32982993 |

| **Adrenal Defect** | | | | | | | |
| --- | --- | --- | --- | --- | --- | --- | --- |
| CYP11A1 | 15q24.11 | 118485 | AR | 613743 | Congenital adrenal insufficiency with partial 46,XY sex reversal (Prader stage 4; 5 or 6) | congential lipoid adrenal hyperplasia | 29178636;26492835;22968487;19116240;21159840;31917682;30299480 |
| CYP11B1 | 8q24.3 | 610613 | AR AD | 202010  193899 | 46,XX Disorders of Sex Development (Prader scale 4; 5 or 6) due to congenital adrenal hyperplasia (11-beta-hydroxylase deficiency) | hyperaldoesteronism, familial, type1; HALD1 | 19153514;18663314;30179867;12966519;8506298;9116420;11095433;11196457;16046588;17172090;17371482;  19567537;20089618;22964742;20523022;20331679;28962970;28228528;27821898;26956189;26476331 |
| CYP17A1 | 10q24.32 | 609300 | AR | 202110 | 46,XY Disorders of Sex Development (Prader stage 4, 5 or 6) due to 17-alpha-hydroxylase/17, 20-lyase deficiency |  | 14504283;20080843;1577471;9326943;1740503;23291414;9360545;37683689;37032508;36721956;36589847;36461073;  36339422;36210947;35682463;35032013;34724156;34627348;34524979 |
| CYP19A1 | 15q21.2 | 107910 | AD | 139300 | Aromatase excess syndrome (pseudohermaphrodism) |  | 24064691;29324451;24705274;12736278;24102311;25415177;25301327;23748068;31377750;21470988;17584767;  15811932;9543166;9211678;12466340;33742552;33351351;30968679;30530883;35837780 |
| CYP19A1 | 15q21.2 | 107910 | AR | 613456 | 46,XX Disorders of Sex Development (Prader scale 4; 5 or 6) due to aromatase deficiency |  | 24064691;29324451;24705274;12736278;24102311;25415177;25301327;23748068;31377750;21470988;17584767;  15811932;9543166;9211678;12466340;33742552;33351351;30968679;30530883;35837780 |
| CYP19A1 | 15q21.2 | 107910 | AR | 613456 | Male infertility in 46,XY men due to aromatase deficiency - per Houston et. al. 2020 |  | 24064691;29324451;24705274;12736278;24102311;25415177;25301327;23748068;31377750;21470988;17584767;  15811932;9543166;9211678;12466340;33742552;33351351;30968679;30530883;35837780 |

| Gene symbol | Locus | OMIM | Inheritan  ce | Phenotype  MIM | GU (or endocrine) Phenotype(s) | Other associated phenotypes | Reference(s) |
| --- | --- | --- | --- | --- | --- | --- | --- |
| CYP21A2 | 6p21.33 | 613815 | AR | 201910 | Adrenal hyperplasia, congenital, due to 21-hydroxylase deficiency (classic) |  | 28879515;28676275;24394723;21843885;21673469;21444649;17895312;17481616;17033937;11443169;26935236;  31571129;31377750;37728028;37548905;37324261;37324257;36992809 |
| CYP21A2 | 6p21.33 | 613815 | AR | 201910 | Hyperandrogenism, nonclassic type, due to 21-hydroxylase deficiency |  | 28879515;28676275;24394723;21843885;21673469;21444649;17895312;17481616;17033937;11443169;26935236;  31571129;31377750;37728028;37548905;37324261;37324257;36992809 |
| NNT | 5p12 | 607878 | AR | 614736 | Glucocorticoid deficiency 4, with or without mineralocorticoid deficiency |  | 27129361;25879317, 26523528 |
| STAR | 8p11.23 | 600617 | AR | 201710 | Lipoid adrenal hyperplasia | n.r. | 28467518;33966470;33536409;23175692;37255966;32867102;8634702;7892608;8948562;11061515;9141542;9215316;  10323391;10566637;14764819;15546900;16968793;17666473 |

| **Testicular Defect** | | | | | | | |
| --- | --- | --- | --- | --- | --- | --- | --- |
| FSHR | 2p16.3 | 136435 | AR AD AR AR | 233300  608115  276400  NA | Ovarian dysgenesis 1  Ovarian hyperstimulation syndrome Ovarian response to FSH stimulation  Hypergonadotropic hypogonadism - Houston et.  al. 2020 |  | 9027356; 9020851; 9202988; 9756276; 10022448; 11804326; 16338864; 23884663; 28591755; 32242295 |

| Gene symbol | Locus | OMIM | Inheritance | Phenotype MIM | GU Phenotype | Other associated phenotypes | Reference(s) |
| --- | --- | --- | --- | --- | --- | --- | --- |
| ADGRG2 | Xp22.13 | 300572 | XL | 300985 | Congenital bilateral absence of vas deferens, X-linked |  | 27476656; 28805948; 30389958; 30811104; 32314195; 32020786; 31845523; 34673937; 37273165; 37489040 |
| *AKR1C2* | 10p15.1 | 600450 | Undetermined | NA - PS 146450 | Hypospadias - houston et. al. 2020 |  | 24986825; 32732174; 21802064; 24793988; 10998348 |
| *AKR1C3* | 10p15.1 | 603966 | Undetermined | NA - PS 146450 | Hypospadias - houston et. al. 2020 |  | 24986825; 32732174 |
| *AKR1C4* | 10p15.1 | 600451 | Undetermined | NA - PS 146450 | Hypospadias - houston et. al. 2020 |  | 24986825; 30496128; 32732174; 10998348 |
| *AKR1D1* | 7q33 | 604741 | AR | 235555  NA - PS 146450 | Hypospadias - houston et. al. 2020 | Bilce acid synthesis defect, congenital, 2 | 18661284 |
| ATF3 | 1q32.3 | 603148 | Undetermined | NA - PS 146450 | Hypospadias - houston et. al. 2020 |  | 18804813; 20215396; 34276780 |
| BMP4 | 14q22.2 | 112262 | AD | 607932  600625 | Microphthalmia, syndromic 6 (micropenis, small scrotum, bifid scrotum, hypoplastic foreskin, hypospadias) | Orofacial cleft 11 | 17033630; 29922894; 31162858; 34129542; 34276780 |
| BMP7 | 20q13.31 | 112267 | AD - houston et. al. 2020 | NA - PS 146450 | Hypospadias - houston et. al. 2020 |  | 17033630; 17213860; 22121287; 30828706; 34276780 |
|  |  |  |  | 618612 | Lower urinary tract obstruction, congenital  (Renal hypodysplasia (in 1 patient), excessive daytime urination, nocturia, urinary incontinence, bladder descensus (in 1 patient), urethral stenosis, posterior urethral valve, vesicoureteral reflux)  - intrafamilial variability Hypospadias - houston et. al. 2020 |  |  |
| BNC2 | 9p22.3-p22.2 | 608669 | AD | NA - PS 146450 |  |  | 21368915; 25383892; 25605705 |
|  |  |  |  |  | none confirmed via omim |  |  |
|  |  |  |  | 143860 | Congenital bilateral absence of vas deferens |  |  |
| CA12 | 15q22.2 | 603263 | AR | NA - PS 277180 | speculated |  | 30632488; 34673937 |
|  |  |  |  |  |  |  | 28801929;28456595;28196530;27793385;26946416;27488005;27364092;26989879;26277102;25944622;25443471;25536748;25386751;25308578; |
|  |  |  |  |  |  |  | 25304080;25010724;24958810;24551851;24451227;24149827;23953609;23687349;23555973;23378603;25246892;22483971;22390181;22340520; |
|  |  |  |  |  |  |  | 22191729;22148899;22103471;21762191;21976147;21679131;21609195;21507732;21254931;21219377;21067729;20609027;20691141;20657600; |
|  |  |  |  |  |  |  | 20381036;20021716;19810821;19737283;19298730;19181743;18810634;18796364;18755906;18304229;18078365;17681314;17673436;17662673; |
|  |  |  |  |  |  |  | 17572159;17823699;17617039;17482604;17448246;17398169;17394391;17329263;17254580;17127107;17050329;16973827;16840743;16714368; |
|  |  |  |  |  |  |  | 16581722;16572913;16481891;16412743;16272798;16128988;15905293;15870824;15705389;15644056;15533383;15482777;15357566;15333598; |
|  |  |  |  |  |  |  | 15239534;15070876;14998948;14747162;15463840;12801574;12741342;12475673;12151438;12009340;11993238;11788091;11574497;11298840; |
|  |  |  |  |  |  | Cystic fibrosis | 11119745;11101688;10923036;10878476;10875853;10871656;10655317;10655318;10653141;28176072;10601093;10376575;10341008;10099982; |
|  |  |  |  | 277180 |  | Sweat chloride elevation without CF | 10066035;10050655;10200050;9797105;9736775;9678705;9630075;9620832;9620832;9598638;9591500;9345100;9334604;9272157;9196095;9067761; |
|  |  |  |  | 219700 |  | Bronchiectasis with or without elevated sweat chloride 1 | 9043501;8627844;8671256;8557264;8829643;8582970;7540706;7539210;7539342;7739684;7539448;7532150;7529962;8556303;7738909;7968122; |
|  |  |  |  | 211400 |  | Hypertrypsinemia, neonatal | 7513294;7516233;8301660;7505692;7692051;8100886;8473422;1545465;17975025;17413420;10653048;8834261;7573058;7542209;7691870;7686336; |
| CFTR | 7q31.2 | 602421 | AR | 167800 | CBAVD | pancreatitis, hereditary | 8421472;1975022;29864494;30450785;29986553;32020786;31823853;31709488;31377750;31357024;30811104;30389601;21875427;10651488 |
|  |  |  |  |  |  |  | 28801929;28456595;28196530;27793385;26946416;27488005;27364092;26989879;26277102;25944622;25443471;25536748;25386751;25308578; |
|  |  |  |  |  |  |  | 25304080;25010724;24958810;24551851;24451227;24149827;23953609;23687349;23555973;23378603;25246892;22483971;22390181;22340520; |
|  |  |  |  |  |  |  | 22191729;22148899;22103471;21762191;21976147;21679131;21609195;21507732;21254931;21219377;21067729;20609027;20691141;20657600; |
|  |  |  |  |  |  |  | 20381036;20021716;19810821;19737283;19298730;19181743;18810634;18796364;18755906;18304229;18078365;17681314;17673436;17662673; |
|  |  |  |  |  |  |  | 17572159;17823699;17617039;17482604;17448246;17398169;17394391;17329263;17254580;17127107;17050329;16973827;16840743;16714368; |
|  |  |  |  |  |  |  | 16581722;16572913;16481891;16412743;16272798;16128988;15905293;15870824;15705389;15644056;15533383;15482777;15357566;15333598; |
|  |  |  |  |  |  |  | 15239534;15070876;14998948;14747162;15463840;12801574;12741342;12475673;12151438;12009340;11993238;11788091;11574497;11298840; |
|  |  |  |  |  |  | Cystic fibrosis | 11119745;11101688;10923036;10878476;10875853;10871656;10655317;10655318;10653141;28176072;10601093;10376575;10341008;10099982; |
|  |  |  |  |  |  | Sweat chloride elevation without CF | 10066035;10050655;10200050;9797105;9736775;9678705;9630075;9620832;9620832;9598638;9591500;9345100;9334604;9272157;9196095;9067761; |
|  |  |  |  |  |  | Bronchiectasis with or without elevated sweat chloride 1 | 9043501;8627844;8671256;8557264;8829643;8582970;7540706;7539210;7539342;7739684;7539448;7532150;7529962;8556303;7738909;7968122; |
|  |  |  |  |  |  | Hypertrypsinemia, neonatal | 7513294;7516233;8301660;7505692;7692051;8100886;8473422;1545465;17975025;17413420;10653048;8834261;7573058;7542209;7691870;7686336; |
| CFTR | 7q31.2 | 602421 | AR |  | Low fertilization and pregnancy rate | pancreatitis, hereditary | 8421472;1975022;29864494;30450785;29986553;32020786;31823853;31709488;31377750;31357024;30811104;30389601;21875427;10651488 |
| CLDN2 | Xq22.3 | 300520 | XLR | 301060 | ?Azoospermia, obstructive, with nephrolithiasis? |  | 31320686 |
| DKK1 | 10q21.1 | 605189 | Undetermined | NA - PS 146450 | Hypospadias - houston et. al. 2020 |  | 25319845; 24479159 |
|  |  |  | AD | 180920 |  |  |  |
|  |  |  | Undetermined | 620193 |  | Aplasia of lacrimal and salivary glands |  |
| FGF10 | 5q12 | 602115 | Undetermined | NA - PS 146450 | Hypospadias - houston et. al. 2020 | LADD syndrome 3 | 17264867; 23727413; 34276780 |
| FGF8 | 10q24.32 | 600483 | Undetermined | NA - PS 146450 | Hypospadias - houston et. al. 2020 |  | 34276780; 31748124; 31200363; 30669598; 26199944; 26031747; 25739677; 25636053; 25064402; 24732674; 23533228; 23200691; 22724017; 21832120;  22035731; 21682876; 20463092; 20553372; 18596921; 17264867; 17235395 |
|  |  |  |  | 609579 |  | ?Scaphocephaly, maxillary retrusion, and impaired |  |
|  |  |  |  | 207410 |  | intellectual development? |  |
|  |  |  | AD | 101200 |  | Antley-Bixler syndrome without genital anomalies or |  |
|  |  |  | AD | 123790 |  | disordered steroidogenesis |  |
|  |  |  | AD | 614592 |  | Apert syndrome |  |
|  |  |  | AD | 101600 |  | Beare-Stevenson cutis gyrata syndrome |  |
|  |  |  | AD |  |  | Bent bone dysplasia syndrome |  |
|  |  |  |  | 123500 |  | Craniofacial-skeletal-dermatologic dysplasia |  |
|  |  |  | AD | 613659 |  | Craniosynostosis, nonspecific |  |
|  |  |  |  | 123150 |  | Crouzon syndrome |  |
|  |  |  | AD | 149730 |  | Gastric cancer, somatic |  |
|  |  |  | AD | 101600 | LADD syndrome 1 (coronal hypospadias) | Jackson-Weiss syndrome |  |
|  |  |  | AD | 101400 |  |  |  |
|  |  |  | AD |  |  | Pfeiffer syndrome |  |
|  |  |  |  |  |  | Saethre-Chotzen syndrome |  |
| FGFR2 | 10q26.13 | 176943 | undetermined | NA - PS 146450 | Isolated hypospadias - per houston et. al. 2020 | Scaphocephaly and Axenfeld-Rieger anomaly | 23727413; 17264867 |
| FKBP4 | 12p13.33 | 600611 | Undetermined | NA - PS 146450 | Hypospadias - houston et. al. 2020 |  | 17343741; 20605780 |
| GLI3 | 7p14.1 | 165240 | AD | NA - PS 146450 | Hypospadias without infertility - houston et. al. 2020 |  | 27502037; 31219235; 9054938; 10441570; 12545275; 10945658 |
| HOXB6 | 17q21.32 | 142961 | Undetermined | NA - PS 146450 | Hypospadias - houston et. al. 2020 |  | 17003840; 25605705 |
|  |  |  |  | NA - PS 146450 | Hypospadias - houston et. al. 2020 |  |  |
|  |  |  |  | 613659 |  |  |  |
| KLF6 | 10p15.2 | 602053 | Undetermined | 176807 | Prostate cancer, somatic | Gastric cancer, somatic | 24986825 |
|  |  |  |  |  | Azoospermia seen in homozygous male variant mice, |  |  |
|  |  |  |  | 607236 | not confirmed in humans |  |  |
|  |  |  | AR | 234200 | Congenital bilateral absence of vas deferens per | HARP syndrome |  |
| PANK2 | 20p13 | 606157 | AR | NA - PS 277180 | Houston Review | Neurodegeneration with brain iron accumulation 1 | 15525657; 19095672 |
| PTN | 7q33 | 162095 | Undetermined | NA - PS 146450 | Hypospadias - houston et. al. 2020 |  | 18661284 |
|  |  |  |  | 211400 |  |  |  |
|  |  |  | AD | 177200 | none confirmed via omim | Bronchiectasis with or without elevated sweat chloride 1 |  |
|  |  |  | AD | 620125 | Congenital bilateral absence of vas deferens | Liddle syndrome 1 |  |
| SCNN1B | 16p12.2 | 600760 | AR | NA - PS 277180 | speculated | Pseudohypoaldosteronism, type IB2, autosomal recessive | 30632488; 34673937 |
| SLC9A3 | 5p15.33 | 182307 | AR | 616868  NA - PS277180 | Congenital bilateral absence of vas deferens per Houston Review | Diarrhea 8, secretory sodium, congenital | 30797621; 30956978; 34673937 |
| WTAP | 6q25.3 | 605442 | AD | NA - PS 146450 | Hypospadias - houston et. al. 2020 |  | 14675924 |
