# Supplementary figures and images for: Gene set enrichment analysis of curated monogenic loci highlights key pathways and multisystem involvement in male infertility
Source: Basic Clin Androl. 2026 Jun 10;36:17. doi: 10.1186/s12610-026-00316-2 (PMC13251197; doi:10.1186/s12610-026-00316-2)

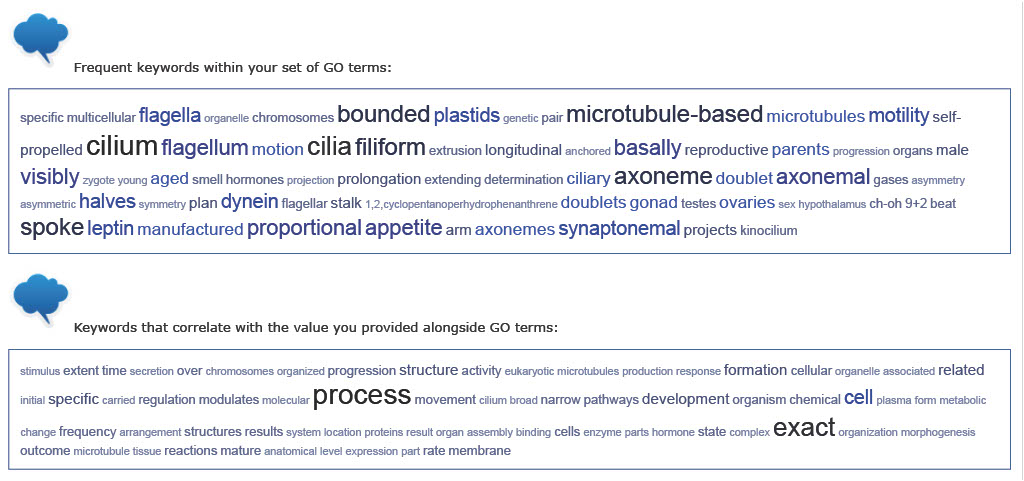

Supplement: Supplementary file 2 — Supplementary Material 2. [file 12610_2026_316_MOESM2_ESM.jpg]
